# An *in vitro* whole-cell electrophysiology dataset of human cortical neurons

**DOI:** 10.1093/gigascience/giac108

**Published:** 2022-11-15

**Authors:** Derek Howard, Homeira Moradi Chameh, Alexandre Guet-McCreight, Huan Allen Hsiao, Maggie Vuong, Young Seok Seo, Prajay Shah, Anukrati Nigam, Yuxiao Chen, Melanie Davie, Etay Hay, Taufik A Valiante, Shreejoy J Tripathy

**Affiliations:** Krembil Centre for Neuroinformatics, Centre for Addiction and Mental Health, Toronto, ON, M5T 1R8, Canada; Krembil Brain Institute, University Health Network, Toronto, ON, M5T 1M8, Canada; Krembil Centre for Neuroinformatics, Centre for Addiction and Mental Health, Toronto, ON, M5T 1R8, Canada; Krembil Centre for Neuroinformatics, Centre for Addiction and Mental Health, Toronto, ON, M5T 1R8, Canada; Krembil Centre for Neuroinformatics, Centre for Addiction and Mental Health, Toronto, ON, M5T 1R8, Canada; Krembil Brain Institute, University Health Network, Toronto, ON, M5T 1M8, Canada; Krembil Brain Institute, University Health Network, Toronto, ON, M5T 1M8, Canada; Krembil Centre for Neuroinformatics, Centre for Addiction and Mental Health, Toronto, ON, M5T 1R8, Canada; Institute of Medical Sciences, Temerty Faculty of Medicine, University of Toronto, Toronto, ON, M5S 1A8, Canada; Krembil Centre for Neuroinformatics, Centre for Addiction and Mental Health, Toronto, ON, M5T 1R8, Canada; Krembil Centre for Neuroinformatics, Centre for Addiction and Mental Health, Toronto, ON, M5T 1R8, Canada; Krembil Centre for Neuroinformatics, Centre for Addiction and Mental Health, Toronto, ON, M5T 1R8, Canada; Institute of Medical Sciences, Temerty Faculty of Medicine, University of Toronto, Toronto, ON, M5S 1A8, Canada; Krembil Brain Institute, University Health Network, Toronto, ON, M5T 1M8, Canada; Institute of Medical Sciences, Temerty Faculty of Medicine, University of Toronto, Toronto, ON, M5S 1A8, Canada; Center for Advancing Neurotechnological Innovation to Application (CRANIA), Toronto, ON, M5S 1A4, Canada; Department of Surgery, Division of Neurosurgery, University of Toronto, Toronto, ON, M5T 1P5, Canada; Institute of Biomedical Engineering, University of Toronto, Toronto, ON, M5S 3G9, Canada; Department of Electrical and Computer Engineering, University of Toronto, Toronto, ON, M5S 3G8, Canada; Max Planck–University of Toronto Center for Neural Science and Technology, Toronto, ON, M5S 1A4, Canada; Center for Advancing Neurotechnological Innovation to Application (CRANIA), Toronto, ON , M5S 1A4, Canada; Krembil Centre for Neuroinformatics, Centre for Addiction and Mental Health, Toronto, ON, M5T 1R8, Canada; Institute of Medical Sciences, Temerty Faculty of Medicine, University of Toronto, Toronto, ON, M5S 1A8, Canada; Department of Surgery, Division of Neurosurgery, University of Toronto, Toronto, ON, M5T 1P5, Canada; Department of Psychiatry, University of Toront, Toronto, ON, M5T 1R8, Canada

## Abstract

**Background:**

Whole-cell patch-clamp electrophysiology is an essential technique for understanding how single neurons translate their diverse inputs into a functional output. The relative inaccessibility of live human cortical neurons for experimental manipulation has made it difficult to determine the unique features of how human cortical neurons differ from their counterparts in other species.

**Findings:**

We present a curated repository of whole-cell patch-clamp recordings from surgically resected human cortical tissue, encompassing 118 neurons from 35 individuals (age range, 21–59 years; 17 male, 18 female). Recorded human cortical neurons derive from layers 2 and 3 (L2&3), deep layer 3 (L3c), or layer 5 (L5) and are annotated with a rich set of subject and experimental metadata. For comparison, we also provide a limited set of comparable recordings from 21-day-old mice (11 cells from 5 mice). All electrophysiological recordings are provided in the Neurodata Without Borders (NWB) format and are available for further analysis via the Distributed Archives for Neurophysiology Data Integration online repository. The associated data conversion code is made publicly available and can help others in converting electrophysiology datasets to the open NWB standard for general reuse.

**Conclusion:**

These data can be used for novel analyses of biophysical characteristics of human cortical neurons, including in cross-species or cross-lab comparisons or in building computational models of individual human neurons.

## Dataset Description

Intracellular electrophysiology, as performed via the whole-cell patch-clamp technique, is a hallmark method for characterizing the biophysical features of neurons. While there have been numerous datasets characterizing these features from cortical neurons in the rodent brain [1–4], comparatively fewer resources provide high-quality whole-cell patch-clamp recordings from human cortical neurons due to the relative inaccessibility of human tissue.

However, collaborations between neurosurgeons and basic neuroscientists have recently made it possible to characterize living cortical neurons in brain slices immediately prepared from biopsies following routine neurosurgery [5–15]. Still, there remain relatively few datasets of human cortical neuron physiology that are openly accessible and free for reuse to complement and compare to the Allen Brain Institute Cell Types Database (Allen Cell Types Database, RRID:SCR_014806) [7].

Here, we describe an openly accessible dataset of electrophysiological recordings from human and mouse cortical neurons. The dataset encompasses 132 whole-cell patch-clamp recordings from surgically resected human tissue (118 cells from 35 individuals) or from 21-day-old mice (11 cells from 5 mice). These datasets are made available in the Neurodata Without Borders (NWB) (RRID:SCR_015242) electrophysiology data format via the Distributed Archives for Neurophysiology Data Integration (DANDI) data archive. We provide morphological reconstructions for *N* = 7 cells, made available at NeuroMorpho.org (RRID:SCR_002145). Each recording is made available with rich subject and experimental protocol metadata, enabling subsequent reuse and comparison with analogous datasets from other species and sources.

## Methods

### Human surgical tissue

Resected human cortical tissues were obtained from Toronto Western Hospital (University Health Network, Canada). All procedures on human tissue were performed in accordance with the Declaration of Helsinki and approved by the University Health Network Research Ethics board [16]. Patients underwent a standardized temporal, parietal, or frontal lobectomy under general anesthesia using volatile anesthetics for seizure or tumor treatment [17, 18]. Tissue was obtained from patients diagnosed with temporal (*n* = 34), frontal (*n* = 1), or parietal lobe (*n* = 1) epilepsy or brain tumors (*n* = 4) in 17 male and 18 female patients, age ranging from 21 to 59 years (mean age ± SD: 40.5 ± 12.0). Written informed consent was obtained from all study participants to use their tissue and to share the acquired data with anonymized demographic information—namely, subject age at time of surgery, sex, years of seizure, seizure frequency, secondarily generalized seizure frequency (using clinical records and epilepsy monitoring unit recordings), antiepileptic drug treatment, and type of seizure.

The resected cortical tissue from the temporal lobe–middle temporal gyrus exhibited no structural or functional abnormalities in preoperative magnetic resonance imaging and was considered “relatively healthy” by ourselves and others as it was located outside of the site of epileptogenesis [6, 9, 12, 13]. Cortical tissue from the frontal cortex from patients with epilepsy was considered “epileptogenic” tissue and confirmed with independent electrocorticography (and annotated as such in our metadata). For tumor cases, cortical tissue blocks were obtained from tissue at a distance from the main site of the tumor (i.e., such cortical tissue was not taken directly from the tumor itself).

### Mouse specimens

All experimental procedures involving mice were reviewed and approved by the animal care committees of the University Health Network in accordance with the guidelines of the Canadian Council on Animal Care. Mixed male and female wild-type C57Bl/6 J, age postnatal 21 days, were used for experiments. Mice were kept on a 12-hour light/dark cycle and had free access to food and water.

### Acute brain slice preparation

Immediately following surgical human cortical resection, the cortical specimens were submerged in an ice-cold (∼4°C) cutting solution that was continuously bubbled with carbogenated (95% O_2_/5% CO_2_) artificial cerebrospinal fluid (aCSF) containing (in mM) the following: sucrose, 248; KCl, 2; MgSO_4_.7H_2_O, 3; CaCl_2_.2H_2_O, 1; NaHCO_3_, 26; NaH_2_PO_4_.H_2_O, 1.25; and D-glucose, 10. The osmolarity was adjusted to 300–305 mOsm. Transverse brain slices (400 μm) were sectioned using a vibratome (Leica 1200 V) Germany in cutting solution. Tissue slicing was performed perpendicular to the pial surface to help ensure that pyramidal cell dendrites were minimally truncated [6, 11, 17]. The cutting solution was the same as used for transport of tissue from the operating room to the laboratory. The time between tissue resection and slice preparation was less than 10 minutes. After sectioning, the slices were incubated for 30 minutes at 34°C in standard aCSF (in mM): NaCl, 123; KCl, 4; CaCl_2_.2H_2_O, 1; MgSO_4_.7H_2_O, 1; NaHCO_3_, 26; NaH_2_PO_4_.H_2_O, 1.2; and D-glucose, 10, pH 7.40. All aCSF and cutting solutions were continuously bubbled with carbogen gas (95% O_2_–5% CO_2_) and had an osmolarity of 300–305 mOsm. Following this incubation, the slices were maintained in standard aCSF at 22–23°C for at least 1 hour, until they were individually transferred to a submerged recording chamber.

Brain slice preparation was done in a similar way for mice and human tissue. Mice were deeply anesthetized by isoflurane 1.5–3.0%. After decapitation, brains were submerged in (∼4°C) cutting solution that was continuously bubbled with 95% O_2_–5% CO_2_ containing (in mM) sucrose, 248; KCl, 2; MgSO_4_.7H_2_O, 3; CaCl_2_.2H_2_O, 1; NaHCO_3_, 26; NaH_2_PO_4_.H_2_O, 1.25; and D-glucose, 10. Mouse somatosensory cortical slices (350 μm) were prepared in the coronal plane similar to human slice preparation as described above.

A subset of cortical slices in both human and mouse was prepared using the N-methyl-D-glucamine (NMDG) protective recovery method [19]. The cortical tissue blocks were transferred and sectioned in 2–4 °C NMDG-HEPES aCSF solution containing (in mM) NMDG, 92; KCl, 2.5; NaH_2_PO_4_, 1.25; NaHCO_3_, 30; HEPES, 20; glucose, 25; thiourea, 2; Na–L-ascorbate, 5; Na-pyruvate, 3; CaCl_2_.4H_2_O, 0.5; and MgSO_4_.7H_2_O 10 (mM). The pH of NMDG-HEPES aCSF solution was adjusted to 7.3–7.4 using hydrochloric acid, and the osmolarity was 300–305 mOsm. The cortical slices were prepared using a vibratome as described above. After slicing, slices were transferred to a recovery chamber filled with 32–34 °C NMDG-HEPES aCSF solution, which continuously bubbled with 95% O_2_–5% CO_2_. After 12 minutes, the slices were transferred to an incubation solution—HEPES aCSF—containing (in mM) NaCl, 92; KCl, 2.5; NaH_2_PO_4_.H_2_O, 1.25; NaHCO_3_, 30; HEPES, 20; glucose, 25; thiourea, 2; Na–L-ascorbate, 5; Na-pyruvate, 3; CaCl_2_.4H_2_O, 2; and MgSO_4_.7H_2_O, 2. After a 1-hour incubation at room temperature, slices were transferred to a recording chamber and continuously perfused with aCSF containing (in mM) NaCl, 126; KCl, 2.5; NaH_2_PO_4_.H_2_O, 1.25; NaHCO_3_, 26; glucose, 12.6; CaCl_2_.2H_2_O, 2; and MgSO_4_.7H_2_O 1 (mM) [6].

### Whole-cell patch-clamp recording from human and mice cortical slices

For electrophysiological recordings, cortical slices were placed in a recording chamber mounted on a fixed-stage upright microscope (Axioskop 2 FS MOT; Carl Zeiss, Germany) Oberkochen, Baden-Württemberg. Slices were continuously perfused with carbogenated (95% O_2_/5% CO_2_) aCSF containing (in mM) NaCl, 123; KCl, 4; CaCl_2_.2H_2_O, 1.5; MgSO_4_.7H_2_O, 1.3; NaHCO_3_, 26; NaH_2_PO_4_.H_2_O, 1.2; and D-glucose, 10, pH 7.40, at 32–34°C. Cortical neurons were visualized using an IR-CCD camera (IR-1000; MTI, USA) Albany, NY with a 40× water immersion objective. Patch pipettes (3–6 MΩ) were pulled from standard borosilicate glass pipettes (thin-wall borosilicate tubes with filaments; World Precision Instruments, Sarasota, FL, USA) using a vertical puller (PC-10; Narishige) Japan. For somatic recording of electrophysiological properties, patch pipettes were filled with intracellular solution containing (in mM) K-gluconate, 135; NaCl, 10; HEPES, 10; MgCl_2_, 1; Na_2_ATP, 2; and GTP 0.3, pH adjusted with KOH to 7.4 (290–309 mOsm). A subset of data was collected with excitatory (APV 50 μM, Sigma [St. Louis, MO, USA]; CNQX 25 μM, Sigma) and inhibitory (Bicuculline 10 μM, Sigma; CGP-35348 10 μM, Sigma) synaptic activity blocked.

Electrical signals were measured with a Multiclamp 700A amplifier, Axopatch 200B amplifier, pClamp 9.2, and pClamp 10.6 data acquisition software (Axon Instruments; Molecular Devices, San Jose, CA, USA). Subsequently, electrical signals were digitized at 20 kHz using a 1320X digitizer or a 1440A digitizer (Axon Instruments; Molecular Devices). The access resistance was monitored throughout the recording (typically between 8 and 20 MΩ), and neurons were discarded if the access resistance was >25 MΩ. Recordings were not corrected for bridge balancing due to the short duration of recording time. We note that stimulus parameters for each recording are not identical across recorded cells, in part due to technical considerations by the experimentalist, for example, to prevent losing the cell recording.

### Axon binary format to NWB file conversion

The x-to-nwb repository was used to convert current clamp recordings in axon binary format (ABF) to NWB format. Separate converters were used for files recorded using pClamp (RRID:SCR_011323) 9.0, which output ABFv1 files, and pClamp >10.0, which output ABFv2 files, to ensure valid conversions while incorporating the essential metadata. The key aspects of our usage of these data conversion computer scripts relate to defining which ABF channels correspond to stimulus and response traces and ensuring that appropriate scale and offset factors are applied properly upon conversion. We incorporated the ndx-dandi-icephys metadata extensions to allow for inclusion of user-defined “Subject” and “Lab” metadata fields to be able to include specific metadata, including “subject_id,” “age,” “species,” “cell_id,” and “tissue_sample_id.”

Relevant metadata were recorded in 2 separate tables: first, patient-level information, including demographics and clinical information, and, second, recording specific information, which relates to aspects of each individual cell's recording, such as channels corresponding to stimulus, response, and resting membrane potential. The patient-level demographics table had fields including “Resection date,” “Resection procedure,” “Sex,” “Age,” “Years of seizure history,” “Diagnosis,” “Seizure type,” “Presence of a tumor,” and “Antiepileptic drugs.” Recording specific metadata included experiment “date,” “Cell number” to differentiate recordings from distinct cells taken on the same day, “Cell layer,” “Gain,” “Offset,” “Response channel,” “Command channel,” and “RMP” to record the resting membrane potential at the initial time of recording. Additional recording metadata were extracted directly from ABF files using custom scripts to extract the stimulus start and end times and the stimulus sampling rate.

### Electrophysiology feature extraction

The Intrinsic Physiology Feature Extractor (IPFX) toolbox was used to extract features from converted NWB files [3, 20]. All experiments consisted of long-square hyperpolarizing and depolarizing current injections, and extracted features included subthreshold features (i.e., input resistance, sag ratio), action potential properties (i.e., action potential half-width, threshold time, and voltage) derived from the rheobase spike as well as multiaction potential spike train features derived from the IPFX-defined “hero” sweep (i.e., adaptation index), as described previously [6]. Our included metadata files contain stimulus start and end times along with an IPFX-compatible stimulus description ontology file for reproducibility and to facilitate the feature extraction process.

### Quality control of contributed neuron recordings

We performed both automated and manual quality control checks of converted recordings to ensure dataset quality and maximize reuse potential. Using features automatically extracted via IPFX, we checked whether the baseline voltage of a sweep (i.e., v_baseline) deviated by more than 10 mV from the initial measure in the first current injection step. Any cell recordings that had any sweep deviate beyond the 10-mV threshold were not included in the final contributed dataset. We also included the measures for maximum drift of baseline Vm in each recording's metadata under the field max_drift_Vm.

Also, individual recordings were manually inspected at 3 injected current steps (the most hyperpolarizing pulse, the rheobase, and the most depolarizing step). In addition, we further manually inspected each neuron recording's frequency/input curve to identify any abnormal responses and also to identify putative recordings from interneurons.

Following this manual inspection process, we note that in some instances, we observed some evidence for spike saturation at higher steps of current injection. We also noted some instances of cells spiking spontaneously (i.e., spiking outside of the window of injected current), but we chose not to reject these sweeps or cells according to our quality control criteria.

### Statistical analyses

To detect statistical differences across experimental groupings, we report results using the 2-sample *t*-test, Wilcoxon rank-sum test, Kruskal–Wallis rank-sum test, or Pearson correlation using the statistical functions in base R.

All statistical tests were performed using R version 4.1.2 [21].

## Results

**Figure 1:**
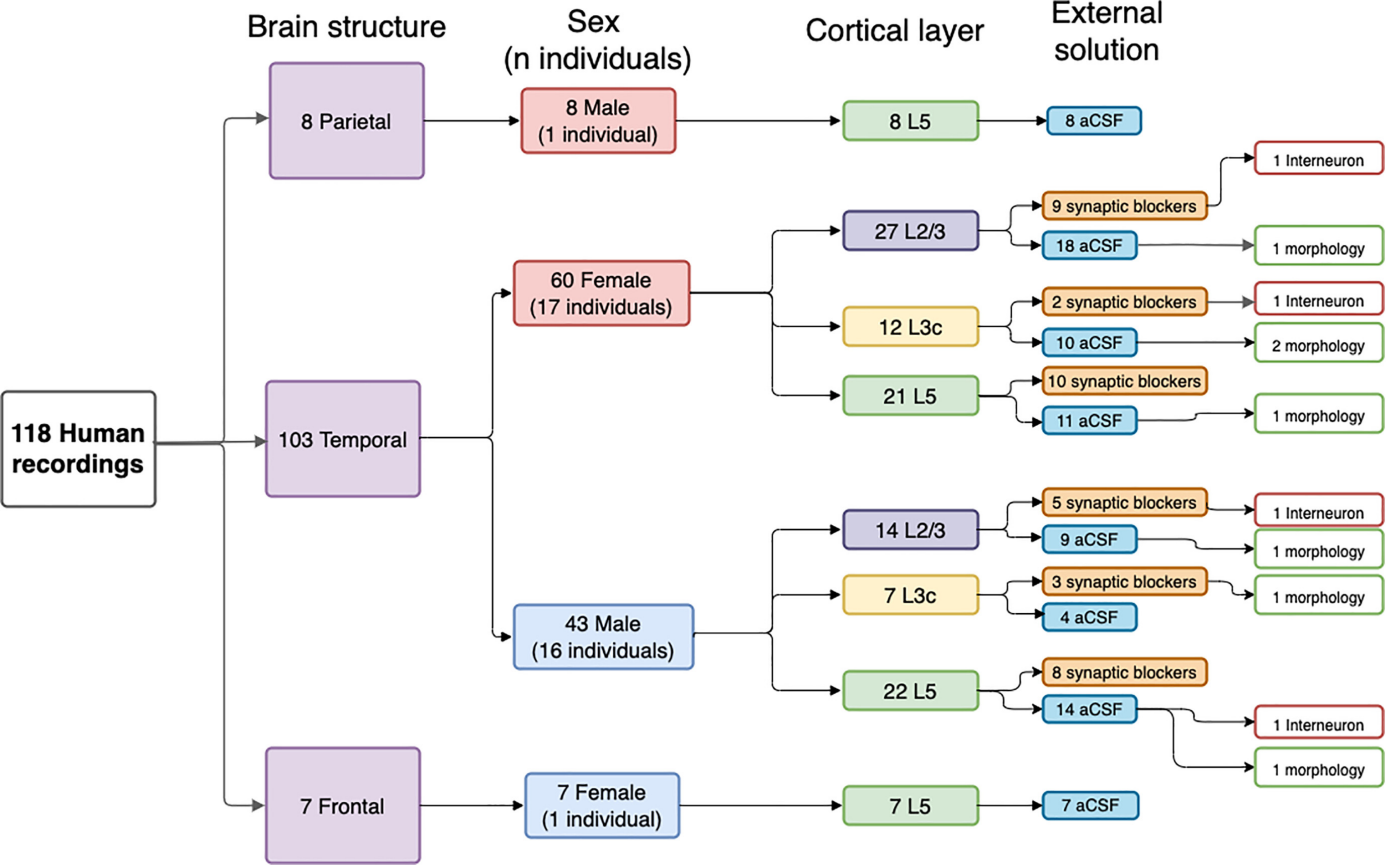
Hierarchical summary of human cell recordings and relevant experimental and technical factors, displaying counts of each recording and associated metadata.

In Table 1, we summarize the 3 main axes differentiating the cells and recordings in this dataset. Namely, recordings differed by species (human versus mouse), by cortical layer of the cell body of the recorded neuron (layer 23, layer 3c, and layer 5), and whether synaptic blockers were used in the external recording solution. Additionally, Fig. 1 illustrates the breakdown of various metadata features associated with the various human electrophysiological recordings. Putative interneurons were identified by their action potential characteristics (large maximal firing rates and typically large spike after-hyperpolarization amplitudes) as described in Chameh et al. [6]. One reason why synaptic blockers were used is to make a subset of recordings more consistent with protocols used in other labs, such as the Allen Institute for Brain Sciences [3, 11].

**Table 1:** Summary of number of electrophysiological recordings across major experimental conditions. Putative interneurons (ints) were identified by manual inspection of their electrophysiological characteristics.

Dataset overall type	# Cells	# Individuals	Number of Pyr cells	Number of ints	L23	L3c	L5
Human (aCSF)	81	19	80	1	27	14	40 (1 int)
Human (aCSF containing synaptic blockers)	37	16	34	3	14 (2 int)	5 (1 int)	18
Mouse (aCSF containing synaptic blockers)	11	5	10	1	0	0	11 (1 int)

**Figure 2:**
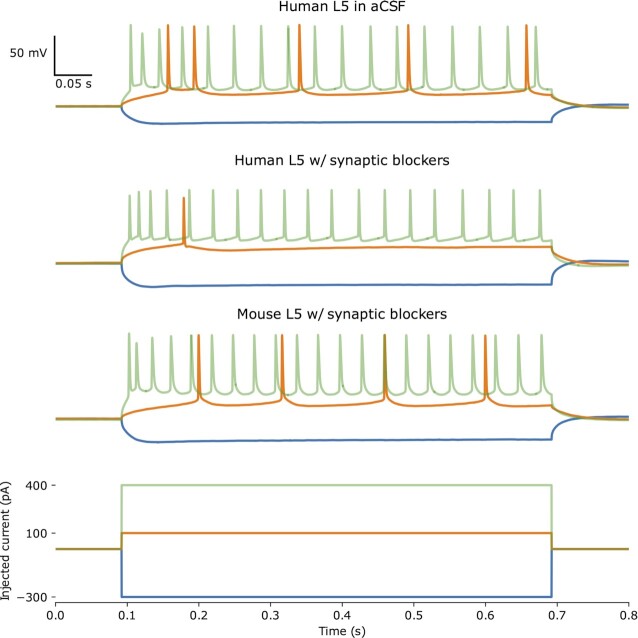
Example voltage traces from 3 separate L5 neurons, a human L5 neuron recorded in standard aCSF (top), a human L5 neuron recorded in aCSF with synaptic blockers (second row), and from a mouse L5 neuron recorded in aCSF with synaptic blockers (third row). Bottom row shows the hyperpolarizing and depolarizing injected step currents that were applied to each neuron and includes the most hyperpolarizing current injection (blue), the rheobase (orange), and most depolarizing current injection (green).

In current clamp mode, hyperpolarizing and depolarizing current injections (600–1,000 ms) were used to characterize biophysical features of cortical neurons, with examples from 3 recorded cells shown in Fig. 2.

**Figure 3:**
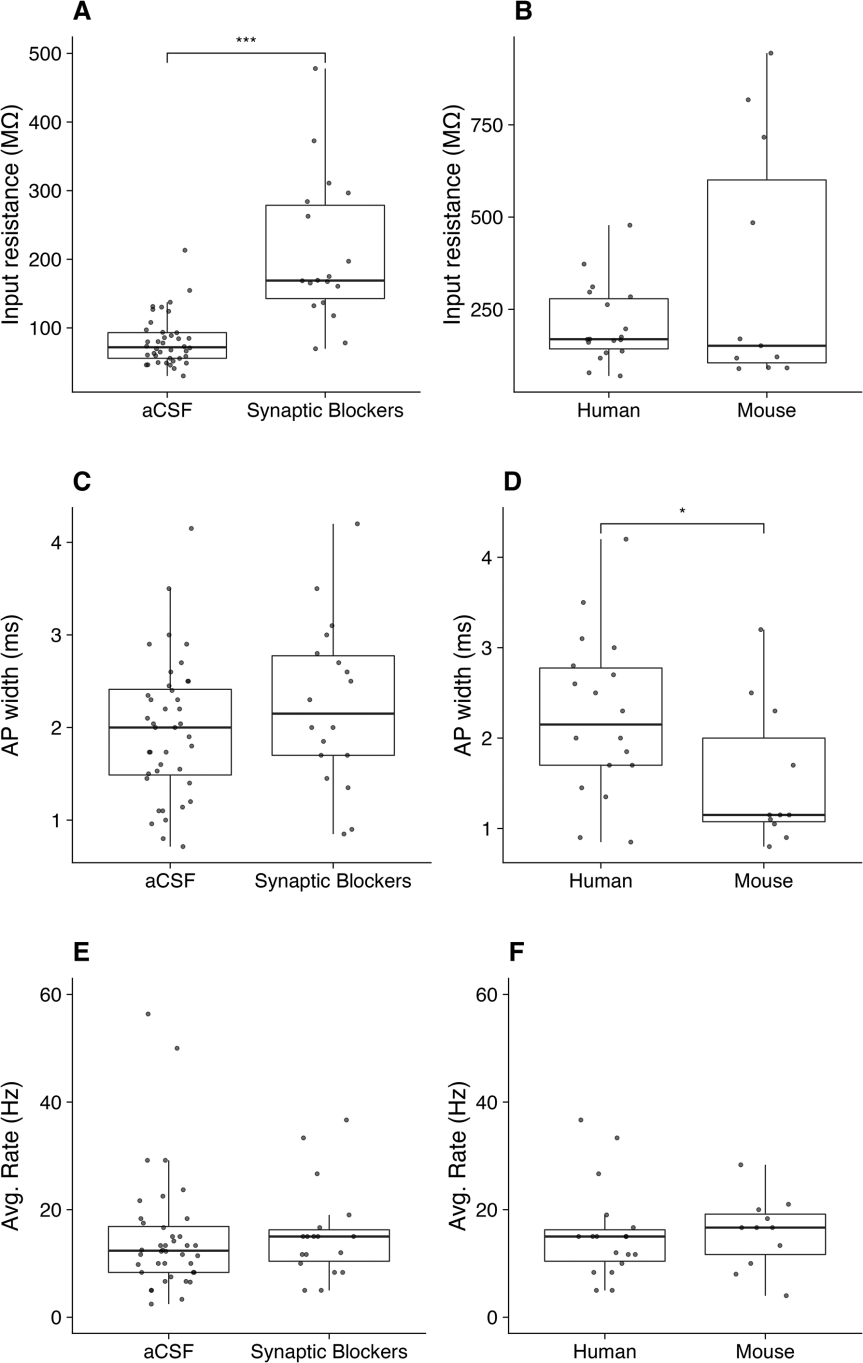
Distributions of input resistance, AP width, and average hero-sweep firing frequency measured in L5 neurons across experimental conditions. Inclusion of synaptic blockers in aCSF has an observable effect on input resistance (A) but not AP width (C) or Avg. Rate (E), as measured in human L5 neurons. Input resistance (B), AP width (D), and Avg. Rate (F) are not significantly different across mouse and human in L5 neurons recorded in the presence of synaptic blockers in the external solution.

In Fig. 3A, we highlight how the use of synaptic blockers in the external solution may affect recorded subthreshold neuronal properties. Specifically, among recorded human L5 neurons, there was a significant difference in the recorded input resistance between neurons recorded following application of synaptic blockers (208 ± 106 MΩ, *n* = 18) and regular aCSF (80.9 ± 36.6 MΩ, *n* = 40); *t*(19) = 4.94, *P* = 9.37e-05. However, in Fig. 3C, there was no significant effect on the action potential width, *t*(28) = 1.14, *P* = 0.265, between neurons recorded following application of synaptics blockers and regular aCSF. Similarly, in Fig. 3E, there was no detectable effect on the average firing rate of the cell at the IPFX-defined “hero” sweep, *t*(40) = 0.259, *P* = 0.797, between the same groups.

To illustrate comparisons across species, in Figs. 3B, D, and F, we show distributions of the input resistance, Action Potential (AP) width, and average firing rate of the “hero” sweep respectively recorded from neurons in both human and mouse cortical L5 neurons (in the presence of synaptic blockers). We did not detect a significant difference in the input resistance, *t*(11) = 1.32, *P* = 0.210, or average firing rate, *t*(25) = 0.0709, *P* = 0.944, observed between the recordings from the 2 species. However, when comparing the width of APs from recordings in human neurons (2.25 ± 0.890 ms, *n* = 18) and mouse neurons (1.55 ± 0.783, *n* = 11), there was a significant difference detected, *t*(23) = 2.23, *P* = 0.0355.

**Figure 4:**
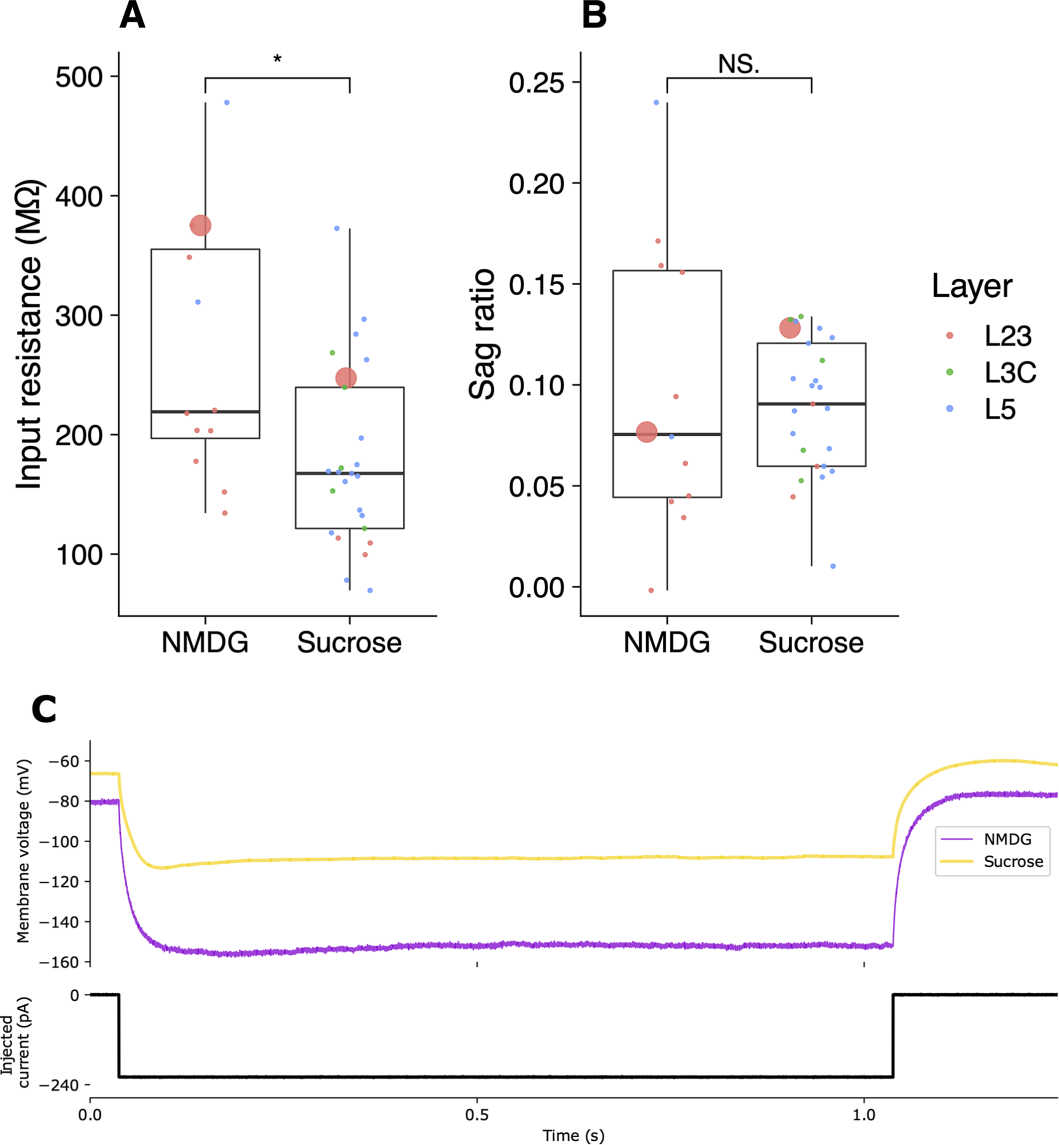
Distributions of input resistance (A) and sag ratio (B) measured in pooled human L2/3, L3C, and L5 neurons derived from different slices, prepared using the NMDG protective solution and standard sucrose solution preparations. All included recordings were performed using synaptic blockers in aCSF recording solution, with the molecular layer of each recording identified in red (L23), green (L3C), and blue (L5). The 2 recordings from L23 highlighted with a large point indicate the recordings used for the example traces of hyperpolarized steps in (C).

To compare the effect of solution used for the brain slice preparation on intrinsic properties, we compared the input resistance and sag ratio recorded following preparation in either solution. In Fig. 4A, we highlight a significant difference, *t*(16) = 2.52, *P* = 0.0224, of higher measured input resistance in the recordings made following preparation in the NMDG (266 ± 108 MΩ, *n* = 12) recovery solution compared to the sucrose solution (179 ± 75.4 MΩ, *n* = 25). In Fig. 4B, we compare the sag ratio across the same conditions and observe no significant difference across the brain slice preparations, *t*(13) = 0.317, *p* = 0.756. The statistical comparisons made in Fig. 4 were made after grouping all recordings from L23, L3C, and L5 using standard aCSF. These comparisons emphasize the potential importance of the conditions used for the experimental preparation (see Discussion).

**Figure 5:**
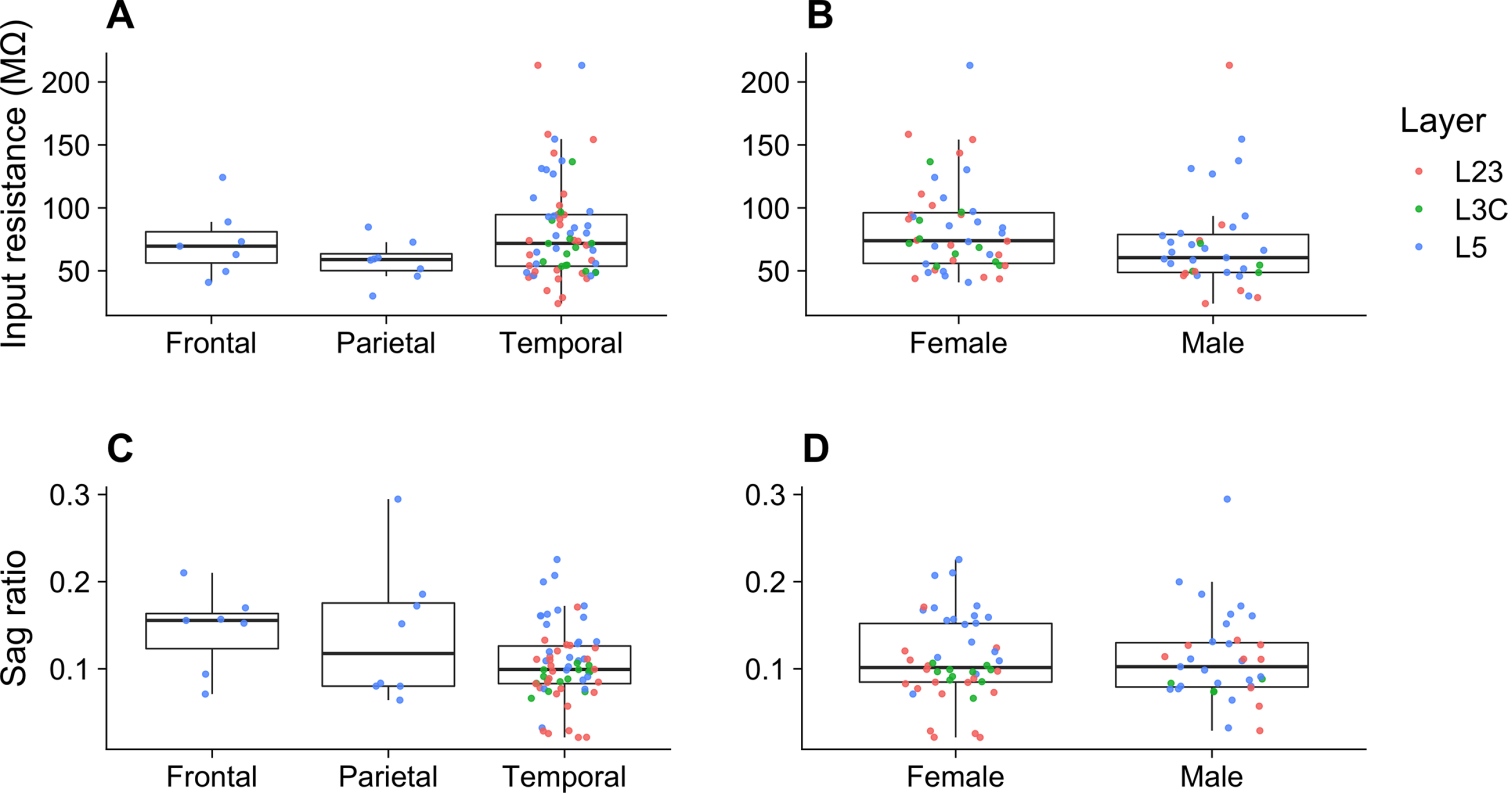
Distributions of input resistance (A, B) and sag ratio (C, D) recorded from pooled human L23, L3C, and L5 neurons with standard aCSF. Measurements are grouped and compared by major brain lobe of resection location (A, C) and by sex (B, D).

To illustrate the rich diversity of the metadata for each of the human recordings, in Fig. 5, we highlight specific comparisons of input resistance and sag ratio measurements recorded in regular aCSF across demographic conditions. Specifically, we focus on the input resistance as a fundamental passive electrophysiological property and the sag ratio as an active property that has previously been used to distinguish between subtypes of human neurons [6, 11]. In Figs. 5A, C, we compare distributions of these electrophysiological features across the 3 different brain lobes from which neuronal tissue was resected. Kruskal–Wallis rank-sum test was used to examine whether brain lobe resection location has a significant effect on input resistance or measured sag ratio. No significant differences in input resistance (χ^2^ = 2.7968, *df* = 2, *P* = 0.247) or sag ratio (χ^2^ = 3.50, *df* = 2, *P* = 0.174) were found across the 3 resected locations. In Figs. 5B, D, we compare the electrophysiological feature distributions measured in male and female patients. We did not detect any differences between recordings from male or female patients in input resistance, *t*(70) = 1.38, *P* = 0.172, or sag ratio, *t*(70) = 0.0644, *P* = 0.949. Note that all cells from frontal and parietal cortices were recorded from tissue resected near the site of the epileptogenic focus, whereas all cells from the temporal cortex were recorded distal from the epileptogenic focus (with the exception of 1 subject).

**Figure 6:**
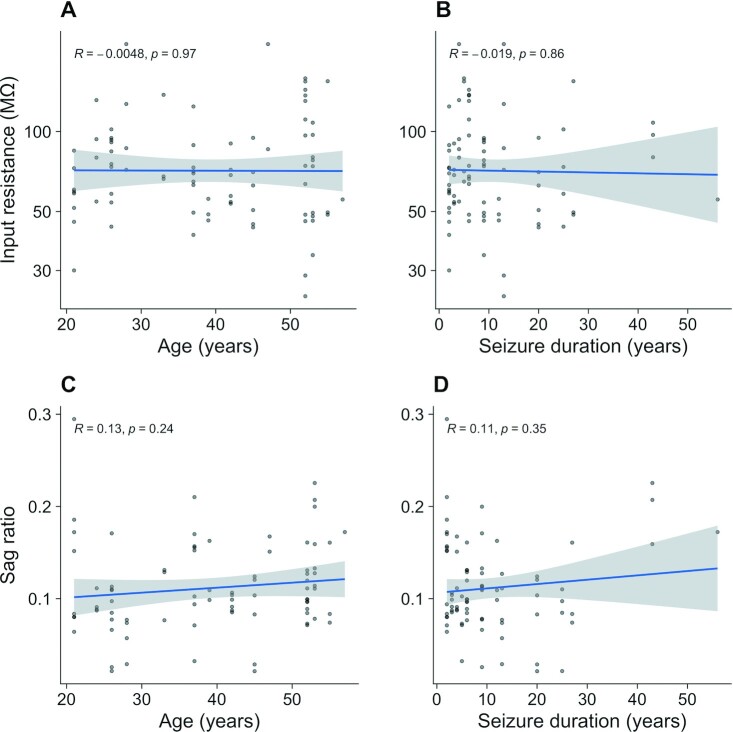
Scatterplots of input resistance and sag ratio versus patient age (A, B) and duration of seizure prior to surgical intervention (C, D) recorded in human L5 neurons with standard aCSF. Pearson's correlation values are reported within each plot.

Additionally, we illustrate the relationship of the input resistance and sag ratio against both patient age at time of surgical resection (Figs. 6A, B) and years of seizure experienced by the patient prior to the surgical intervention (Figs. 6C, D).

### Application scenarios

The recordings in this database permit the quantification of biophysical properties from a diverse set of neurons, including human and mouse neurons with a well-described set of metadata. Independent variables collected include age, sex, seizure history, and cortical layer from which the tissue was resected. Additionally, experiments on the human neurons were performed with the use of synaptic blockers and without, allowing for comparisons and integration with other intrinsic electrophysiological databases comprising patch-clamp recordings, including from the Allen Cell Types Database (RRID:SCR_014806).

These data from current-clamp experiments are particularly beneficial for the development of conductance-based models of human neurons [11, 22, 23]. In particular, we highlight that in some instances, it may be more suitable to constrain biophysical models to human data in the absence of synaptic blockers, that is to say, when background synaptic activity is having a significant effect on input resistance measurements. The voltage responses can be used as a training set to constrain biophysical models when combined and integrated with other publicly available databases that provide relevant morphologies and channel kinetics, such as NeuroMorpho.org, Channelpedia, or ICGenealogy [24–26]. Moreover, fitting biophysical models to data that are grouped based on demographic information can allow for cross-group comparisons using *in silico*approaches. Usage of these models in neuronal or circuit simulations can thus help to further predict and unveil the potential effects of differences in neuronal physiology across demographic groupings [27, 28].

### Discussion and Limitations

The repository provided is focused mainly on cortical neurons derived from human tissue. There are comparatively fewer recordings for analyses of mouse neuron function provided, and all of these were performed using synaptic blockers that were shown to have a baseline effect on input resistance.

The recordings from human specimens derived from tissue during the surgical resection of diseased tissue for patients with intractable epilepsy or brain tumors. Along with having suffered seizures for an extended period of time, the patients may have concurrently taken 1 or a variety of antiepileptic drugs that could have affected baseline neuronal excitability characteristics.

While these data were collected for the purpose of characterizing intrinsic properties of human neocortical neurons, we note that they were collected using different sets of experimental conditions, including those related to different recording solutions as well as cutting solutions. Our analyses suggest such experimental condition differences likely contribute to differences in downstream electrophysiological properties and are consistent with prior analyses by ourselves and others [2, 29]. For example, the observed effect of synaptic blockers on the input resistance may be due to reduction of overall membrane permeability as a consequence of the block of both excitatory and inhibitory conductances [30]. However, we did not observe a concurrent change in excitability characteristics such as the AP width, in agreement with previous findings that did not find a significant effect of synaptic blockers on AP characteristics or neuronal passive properties [31, 32].

In contrast, the comparisons of electrophysiological measures following different cutting solutions highlight potential effects on neuronal excitability. We observe variability in the measured input resistance but consider that these effects may be due to changes in conductivity across the membrane or also experimental biases in selection of healthy neurons for patch-clamp protocol due to differential response to solutions of different osmolarity. Furthermore, there exists contrasting results in the literature on the effects of NMDG on neuronal excitability and synaptic transmission, which shows the context dependence of the many experimental variables [33, 34]. Taken together, such potential differences in electrophysiological characteristics due to experimental conditions are important to consider when reusing these data in downstream analyses.

## Abbreviations

ABF: axon binary format; aCSF: artificial cerebrospinal fluid; DANDI: Distributed Archives for Neurophysiology Data Integration; IPFX: Intrinsic Physiology Feature Extractor; NMDG: N-methyl-D-glucamine; NWB: Neurodata Without Borders.

## Data Availability

Both mouse and human data are available on the DANDI platform [35, 36].

Conversion, analysis scripts, and other associated metadata for recordings are available at GitHub [37]. All other supporting data and materials are available in the *GigaScience* GigaDB database [38]

## Authors’ Contributions

Derek Howard Conceptualization, Project Administration, Formal Analysis, Software, Investigation, Validation, Data Curation, Writing—Original Draft Preparation, Writing—Review & Editing, Visualization Homeira Moradi Chameh Project Administration, Investigation, Data Curation, Writing—Review & Editing Alexandre Guet-McCreight Investigation, Visualization Huan Allen Hsiao Software, Investigation Maggie Vuong Software, Investigation Young Seok Seo Software, Investigation Prajay Shah Investigation Anukrati Nigam Investigation Yuxiao Chen Investigation Melanie Davie Investigation Etay Hay Writing—Review & Editing  Taufik A Valiante Supervision, Resources, Funding Acquisition, Writing—Review & Editing Shreejoy Tripathy Conceptualization, Supervision, Methodology, Validation, Resources, Funding Acquisition, Writing—Original Draft Preparation , Writing—Review & Editing , Visualization.

## Supplementary Material

giac108_GIGA-D-22-00068_Original_Submission

giac108_GIGA-D-22-00068_Revision_1

giac108_GIGA-D-22-00068_Revision_2

giac108_Response_to_Reviewer_Comments_Original_Submission

giac108_Response_to_Reviewer_Comments_Revision_1

giac108_Reviewer_1_Report_Original_Submission Koen Kole -- 5/11/2022 Reviewed

giac108_Reviewer_2_Report_Original_Submission Nathan Gouwens -- 5/13/2022 Reviewed

giac108_Reviewer_2_Report_Revision_1 Koen Kole -- 8/23/2022 Reviewed

## References

[1] Tripathy SJ, Savitskaya J, Burton SD, et al. NeuroElectro: a window to the world's neuron electrophysiology data. Front Neuroinform. 2014;8. 40 24808858 10.3389/fninf.2014.00040 PMC4010726

[2] Tripathy SJ, Burton SD, Geramita M, et al. Brain-wide analysis of electrophysiological diversity yields novel categorization of mammalian neuron types. J Neurophysiol. 2015;113:3474–89. 25810482 10.1152/jn.00237.2015 PMC4455486

[3] Gouwens NW, Sorensen SA, Berg J, et al. Classification of electrophysiological and morphological neuron types in the mouse visual cortex. Nat Neurosci. 2019;22:1182–95. 31209381 10.1038/s41593-019-0417-0 PMC8078853

[4] Markram H, Muller E, Ramaswamy S, et al. Reconstruction and simulation of neocortical microcircuitry. Cell. 2015;163:456–92. 26451489 10.1016/j.cell.2015.09.029

[5] Mohan H, Verhoog MB, Doreswamy KK, et al. Dendritic and axonal architecture of individual pyramidal neurons across layers of adult human neocortex. Cereb Cortex. 2015;25:4839–53. 26318661 10.1093/cercor/bhv188 PMC4635923

[6] Chameh HM, Rich S, Wang L, et al. Diversity amongst human cortical pyramidal neurons revealed via their sag currents and frequency preferences. Nat Commun. 2021;12:1–15. 33941783 10.1038/s41467-021-22741-9 PMC8093195

[7] Berg J, Sorensen SA, Ting JT, et al. Human neocortical expansion involves glutamatergic neuron diversification. Nature. 2021;598:151–8. 34616067 10.1038/s41586-021-03813-8 PMC8494638

[8] Gidon A, Zolnik TA, Fidzinski P, et al. Dendritic action potentials and computation in human layer 2/3 cortical neurons. Science. 2020;367:83–7. 31896716 10.1126/science.aax6239

[9] Beaulieu-Laroche L, Toloza EHS, van der Goes M-S, et al. Enhanced dendritic compartmentalization in human cortical neurons. Cell. 2018:3:643–651. 10.1016/j.cell.2018.08.045 PMC6197488 30340039

[10] Beaulieu-Laroche L, Brown NJ, Hansen M, et al. Allometric rules for mammalian cortical layer 5 neuron biophysics. Nature. 2021:600.7888:274–278. 34759318 10.1038/s41586-021-04072-3 PMC8665137

[11] Kalmbach BE, Buchin A, Long B, et al. h-Channels contribute to divergent intrinsic membrane properties of supragranular pyramidal neurons in human versus mouse cerebral cortex. Neuron. 2018;100:1194–208.. e5. 30392798 10.1016/j.neuron.2018.10.012 PMC6447369

[12] Kalmbach BE, Hodge RD, Jorstad NL, et al. Signature morpho-electric, transcriptomic, and dendritic properties of human layer 5 neocortical pyramidal neurons. Neuron.2021; 109.18:2914–2927. 34534454 10.1016/j.neuron.2021.08.030 PMC8570452

[13] Hodge RD, Bakken TE, Miller JA, et al. Conserved cell types with divergent features in human versus mouse cortex. Nature. 2019;573:61–8. 31435019 10.1038/s41586-019-1506-7 PMC6919571

[14] Deitcher Y, Eyal G, Kanari L, et al. Comprehensive morpho-electrotonic analysis shows 2 distinct classes of L2 and L3 pyramidal neurons in human temporal cortex. Cereb Cortex. 2017;27:5398–414. 28968789 10.1093/cercor/bhx226 PMC5939232

[15] Florez CM, McGinn RJ, Lukankin V, et al. In vitro recordings of human neocortical oscillations. Cereb Cortex. 2015;25:578–97. 24046077 10.1093/cercor/bht235

[16] Mansouri A, Fallah A, Valiante TA. Determining surgical candidacy in temporal lobe epilepsy. Epilepsy Res Treat. 2012;2012:1–16. 10.1155/2012/706917 PMC3420473 22957238

[17] Kostopoulos G, Drapeau C, Avoli M, et al. Endogenous adenosine can reduce epileptiform activity in the human epileptogenic cortex maintained in vitro. Neurosci Lett. 1989;106:119–24. 2586817 10.1016/0304-3940(89)90212-7

[18] Köhling R, Avoli M. Methodological approaches to exploring epileptic disorders in the human brain in vitro. J Neurosci Methods. 2006;155:1–19. 16753220 10.1016/j.jneumeth.2006.04.009

[19] Ting JT, Lee BR, Chong P, et al. Preparation of acute brain slices using an optimized N-methyl-D-glucamine protective recovery method. J Visual Exp. 2018. 132:e53825 10.3791/53825 PMC5931343 29553547

[20] Lee BR, Budzillo A, Hadley K, et al. Scaled, high fidelity electrophysiological, morphological, and transcriptomic cell characterization. Elife. 2021. 10:e65482 34387544 10.7554/eLife.65482 PMC8428855

[21] R Core Team . R: A language and environment for statistical computing. Vienna: R Foundation for Statistical Computing; 2013.

[22] Eyal G, Verhoog MB, Testa-Silva G, et al. Unique membrane properties and enhanced signal processing in human neocortical neurons. Elife. 2016. 5:e16553 27710767 10.7554/eLife.16553 PMC5100995

[23] Yao HK, Guet-McCreight A, Mazza F, et al. Reduced inhibition in depression impairs stimulus processing in human cortical microcircuits. Cell Rep. 2022;38:110232. 35021088 10.1016/j.celrep.2021.110232

[24] Ascoli GA, Donohue DE, Halavi M. Org: a central resource for neuronal morphologies. J Neurosci. 2007;27:9247–51. 17728438 10.1523/JNEUROSCI.2055-07.2007 PMC6673130

[25] Ranjan R, Khazen G, Gambazzi L, et al. Channelpedia: an integrative and interactive database for ion channels. Front Neuroinform. 2011;5. 36 22232598 10.3389/fninf.2011.00036 PMC3248699

[26] Podlaski WF, Seeholzer A, Groschner LN, et al. Mapping the function of neuronal ion channels in model and experiment. eLife.2017; 26:e22152 10.7554/eLife.22152 PMC5340531 28267430

[27] Goriounova NA, Heyer DB, Wilbers R, et al. Large and fast human pyramidal neurons associate with intelligence. Elife. 2018. 7:e41714 30561325 10.7554/eLife.41714 PMC6363383

[28] Guet-McCreight A, Chameh HM, Mahallati S, et al. Age-dependent increased sag current in human pyramidal neurons dampens baseline cortical activity. bioRxiv. 10.1093/cercor/bhac348 36124673

[29] Tebaykin D, Tripathy SJ, Binnion N, et al. Modeling sources of interlaboratory variability in electrophysiological properties of mammalian neurons. J Neurophysiol. 2018;119:1329–39. 29357465 10.1152/jn.00604.2017 PMC5966732

[30] Núñez-Abades PA, Pattillo JM, Hodgson TM, et al. Role of synaptic inputs in determining input resistance of developing brain stem motoneurons. J Neurophysiol. 2000;84:2317–29. 11067975 10.1152/jn.2000.84.5.2317

[31] Ashwood TJ, Wheal HV. The expression of N-methyl-d-aspartate-receptor-mediated component during epileptiform synaptic activity in the hippocampus. Br J Pharmacol.1987; 91:815–822. 2889490 10.1111/j.1476-5381.1987.tb11280.x PMC1853572

[32] Nedergaard S . Regulation of action potential size and excitability in substantia nigra compacta neurons: sensitivity to 4-aminopyridine. J Neurophysiol. 1999;82:2903–13. 10601428 10.1152/jn.1999.82.6.2903

[33] Thuma JB, Hooper SL. Choline and NMDG directly reduce outward currents: reduced outward current when these substances replace Na+ is alone not evidence of Na+-activated K+ currents. J Neurophysiol. 2018;120:3217–33. 30354793 10.1152/jn.00871.2017

[34] Avegno EM, Middleton JW, Gilpin NW. Synaptic GABAergic transmission in the central amygdala (CeA) of rats depends on slice preparation and recording conditions. Physiol Rep. 2019.7:e14245 31587506 10.14814/phy2.14245 PMC6778595

[35] Howard D, Chameh HM, Taufik V, et al. UHN whole-cell patch-clamp excitability recordings from human cortical neurons (Version 0.220708.1652) [Data set]. DANDI Archive. 2022. 10.48324/dandi.000293/0.220708.1652.

[36] Howard D, Chameh HM, Moradi H, et al. UHN whole-cell patch-clamp excitability recordings from mouse cortical neurons (Version 0.220708.1652) [Data set]. DANDI Archive. 2022. 10.48324/dandi.000292/0.220708.1652.

[37] Associated metadata for recordings in GitHub. https://github.com/derekhoward/nwb_conversion/blob/master/data/processed/meta/metadata.csv, Accessed 12 August, 2022.

[38] Howard D, Chameh HM, Guet-McCreight A, et al. Supporting data for “An in vitro whole-cell electrophysiology dataset of human cortical neurons.”. GigaScience Database. 2022. 10.5524/102317. PMC9664072 36377463

