# Supplementary material for: An in vitro whole-cell electrophysiology dataset of human cortical neurons
Source: Gigascience. 2022 Nov 15;11:giac108. doi: 10.1093/gigascience/giac108 (PMC9664072; doi:10.1093/gigascience/giac108)

GigaScience

An in vitro whole-cell electrophysiology dataset of human cortical neurons

--Manuscript Draft--

**Manuscript Number:**	GIGA-D-22-00068
**Full Title:**	An in vitro whole-cell electrophysiology dataset of human cortical neurons
**Article Type:**	Data Note
**Funding Information:**	Centre for Addiction and Mental Health (Discovery Fund) — Dr Shreejoy Tripathy<br>Krembil Brain Institute Fund — Dr Taufik A Valiante<br>National Institute of Health — Dr Taufik A Valiante<br>Kavli Foundation — Dr Shreejoy Tripathy

Abstract:

Background

Whole-cell patch-clamp electrophysiology is an essential technique for understanding how single neurons translate their diverse inputs into a functional output. The relative inaccessibility of live human cortical neurons for experimental manipulation has made it difficult to determine the unique features of how human cortical neurons differ from their counterparts in other species.

Findings

We present a curated repository of whole-cell patch-clamp recordings from surgically resected human cortical tissue, encompassing 129 neurons from 36 individuals (age ranging 21-59 years old; 18 male, 18 female). Recorded human cortical neurons derive from layers 2&3 (L2&3), deep layer 3 (L3c) or layer 5 (L5) of the neocortex and are annotated with a rich set of subject and experimental metadata. For comparison, we also provide a limited set of comparable recordings from 21-day old mice (12 cells from 5 mice). All electrophysiological recordings are provided in the Neurodata Without Borders (NWB) format and are available for further analysis via the Distributed Archives for Neurophysiology Data Integration (DANDI) online archive. The associated data conversion code is made publicly available and can help others in converting electrophysiology datasets to the open NWB standard for general re-use.

Conclusion

These data can be used for novel analyses of biophysical characteristics of human cortical neurons including in cross-species or cross-lab comparisons or in building computational models of individual human neurons.

**Corresponding Author:**	Derek Howard<br>CAMH: Centre for Addiction and Mental Health<br>Toronto, Ontario CANADA
**Corresponding Author Secondary Information:**	
**Corresponding Author's Institution:**	CAMH: Centre for Addiction and Mental Health
**Corresponding Author's Secondary Institution:**	
**First Author:**	Derek Howard
**First Author Secondary Information:**	
**Order of Authors:**	Derek Howard
	Homeira Moradi Chameh
	Alexandre Guet-McCreight

Powered by Editorial Manager® and ProduXion Manager® from Aries Systems Corporation

	Huan Allen Hsiao
	Maggie Vuong
	Young Seok Seo
	Prajay Shah
	Anukrati Nigam
	Yuxia Chen
	Melanie Davie
	Etay Hay
	Taufik A Valiante
	Shreejoy Tripathy
**Order of Authors Secondary Information:**	

**Additional Information:**	
**Question**	**Response**
Are you submitting this manuscript to a special series or article collection?	No
**Experimental design and statistics**<br><br>Full details of the experimental design and statistical methods used should be given in the Methods section, as detailed in our Minimum Standards Reporting Checklist. Information essential to interpreting the data presented should be made available in the figure legends.<br><br>Have you included all the information requested in your manuscript?	Yes
**Resources**<br><br>A description of all resources used, including antibodies, cell lines, animals and software tools, with enough information to allow them to be uniquely identified, should be included in the Methods section. Authors are strongly encouraged to cite Research Resource Identifiers (RRIDs) for antibodies, model organisms and tools, where possible.<br><br>Have you included the information requested as detailed in our Minimum Standards Reporting Checklist?	Yes

Powered by Editorial Manager® and ProduXion Manager® from Aries Systems Corporation

**Availability of data and materials**<br><br>All datasets and code on which the conclusions of the paper rely must be either included in your submission or deposited in publicly available repositories (where available and ethically appropriate), referencing such data using a unique identifier in the references and in the “Availability of Data and Materials” section of your manuscript.<br><br>Have you have met the above requirement as detailed in our Minimum Standards Reporting Checklist?	Yes

Powered by Editorial Manager® and ProduXion Manager® from Aries Systems Corporation

Manuscript Click here to access/download;Manuscript;manuscript.docx

An in vitro whole-cell electrophysiology dataset of human cortical neurons

Abstract

Background

Whole-cell patch-clamp electrophysiology is an essential technique for understanding how single neurons translate their diverse inputs into a functional output. The relative inaccessibility of live human cortical neurons for experimental manipulation has made it difficult to determine the unique features of how human cortical neurons differ from their counterparts in other species.

Findings

We present a curated repository of whole-cell patch-clamp recordings from surgically resected human cortical tissue, encompassing 129 neurons from 36 individuals (age ranging 21-59 years old; 18 male, 18 female). Recorded human cortical neurons derive from layers 2&3 (L2&3), deep layer 3 (L3c) or layer 5 (L5) of the neocortex and are annotated with a rich set of subject and experimental metadata. For comparison, we also provide a limited set of comparable recordings from 21-day old mice (12 cells from 5 mice). All electrophysiological recordings are provided in the Neurodata Without Borders (NWB) format and are available for further analysis via the Distributed Archives for Neurophysiology Data Integration (DANDI) online archive. The

associated data conversion code is made publicly available and can help others in converting electrophysiology datasets to the open NWB standard for general re-use.

Conclusion

These data can be used for novel analyses of biophysical characteristics of human cortical neurons including in cross-species or cross-lab comparisons or in building computational models of individual human neurons.

Authors

Derek Howard[1]*, Homeira Moradi Chameh[2]*, Alexandre Guet-McCreight[1], Huan Allen Hsiao[1], Maggie Vuong[1], Young Seok Seo[2], Prajay Shah[2], Anukrati Nigam[1,5], Yuxiao Chen[1], Melanie Davie[1], Etay Hay[1,3], Taufik A Valiante[2,3,5,6,7,8,9,10]*, Shreejoy J Tripathy[1,3,4,5]*

[1] *Krembil Centre for Neuroinformatics, Centre for Addiction and Mental Health*

[2] *Krembil Brain Institute, University Health Network, Toronto, ON Canada*

[3] *Department of Physiology, University of Toronto*

[4] *Department of Psychiatry, University of Toronto.*

[5] *Institute of Medical Sciences, Temerty Faculty of Medicine, University of Toronto.*

[6] *Department of Surgery, Division of Neurosurgery, University of Toronto*

[7] *Institute of Biomedical Engineering, University of Toronto*

[8] *Department of Electrical and Computer Engineering, University of Toronto*

[9] *Max Planck - University of Toronto Center for Neural Science and Technology, Toronto, Canada*

[10] *Center for Advancing Neurotechnological Innovation to Application (CRANIA), Toronto,Canada*

**Denotes equal contribution*

Dataset Description

Intracellular electrophysiology, as performed via the whole-cell patch-clamp technique, is a hallmark method for characterizing the biophysical features of neurons. While there have been numerous datasets characterizing these features from cortical neurons in the rodent brain [1]–[4], there are comparatively fewer resources that provide high-quality whole-cell patch-clamp recordings from human cortical neurons due to the relative inaccessibility of human tissue.

However, collaborations between neurosurgeons and basic neuroscientists have recently made it possible to characterize living cortical neurons in brain slices immediately prepared from biopsies following routine neurosurgery [5]–[15]. Still, there remain relatively few datasets of human cortical neuron physiology that are openly-accessible and free for reuse to complement and compare to the the Allen Brain Institute Cell Types Database (http://celltypes.brain-map.org/) [7].

Here, we describe an openly-accessible dataset of electrophysiological recordings from human and mouse cortical neurons. The dataset encompasses 141 whole-cell patch-clamp recordings from surgically resected human tissue (129 cells from 36 individuals) or from 21-day old mice (12 cells from 5 mice). These datasets are made available in the Neurodata Without Borders (NWB) electrophysiology data format via the DANDI data archive. We provide morphological reconstructions for N = 7 cells, made available at NeuroMorpho.org. Each recording is made available with rich subject and experimental protocol metadata, enabling subsequent reuse and comparison with analogous datasets from other species and sources.

Figure 1: Hierarchical summary of human cell recordings and relevant experimental and technical factors, displaying counts of each recording and associated metadata

Methods

Ethics, consent and permissions

Mouse specimens

All experimental procedures involving mice were reviewed and approved by the animal care committees of the University Health Network in accordance with the guidelines of the Canadian Council on Animal Care. Mixed male and female wild type C57Bl/6J, age postnatal 21 days old were used for experiments. Mice were kept on a 12-hour light/dark cycle and had free access to food and water.

Human surgical tissue

Resected human cortical tissues were obtained from Toronto Western Hospital (University Health Network, Canada). All procedures on human tissue were performed in accordance with the Declaration of Helsinki and approved by the University Health Network Research Ethics board [19]. Patients underwent a standardized temporal, parietal or frontal lobectomy under general anesthesia using volatile anesthetics for seizure or tumor treatment [20,21]. Tissue was obtained from patients diagnosed with temporal (n=34), frontal (n=1) or parietal lobe (n=1) epilepsy or brain tumors (n=4) in 18 male and 18 female patients, age ranging 21 to 59 years (mean age ± SD: 40.22 ± 12.00).

The resected cortical tissue from the temporal lobe-middle temporal gyrus (MTG) exhibited no structural or functional abnormalities in preoperative MRI, and is considered “relatively healthy” by ourselves and others as it is located outside of the site of epileptogenesis [6,9,12,13]. Cortical tissue from the frontal cortex from patients with epilepsy are considered as “epileptogenic” tissue and are confirmed with independent electrocorticography (ECoG) (and are annotated as such in our metadata). For tumor cases, cortical tissue blocks were obtained from tissue at a distance from the main site of the tumor (i.e., such cortical tissue was not taken directly from the tumor itself).

Consent to publish

Written informed consent were obtained from all study participants to use their tissue and to share the acquired data with anonymized demographic information - namely, subject age at time of surgery, sex, years of seizure, seizure frequency, secondarily generalized seizure frequency (using clinical records and epilepsy monitoring unit recordings), antiepileptic drug treatment, and type of seizure.

Acute brain slice preparation

Immediately following surgical human cortical resection, the cortical specimens were submerged in an ice-cold (~4°C) cutting solution that was continuously bubbled with carbogenated (95% O_2/5% CO_2) aCSF containing (in mM) sucrose 248, KCl 2, $MgSO_4.7H_2O$ 3, $CaCl_2.2H_2O$ 1, $NaHCO_3$ 26, $NaH_2PO_4.H_2O$ 1.25, D-glucose 10. The osmolarity was adjusted to 300-305 mOsm. Transverse brain slices (400 µm) were sectioned using a vibratome (Leica 1200 V) in cutting solution. Tissue slicing was performed perpendicular to the pial surface to help ensure that pyramidal cell dendrites were minimally truncated [6], [11], [17]. The cutting

solution was the same as used for transport of tissue from the operating room to the laboratory. The time between tissue resection and slice preparation was less than 10 min. After sectioning, the slices were incubated for 30 min at 34 °C in standard artificial cerebrospinal fluid (aCSF) (in mM): NaCl 123, KCl 4, $CaCl_2.2H_2O$ 1, $MgSO_4$.7H2O 1, $NaHCO_3$ 26, NaH_2PO_4.H2O 1.2, and D-glucose 10, pH 7.40. All aCSF and cutting solutions were continuously bubbled with carbogen gas (95% O_2-5% CO_2) and had an osmolarity of 300-305 mOsm. Following this incubation, the slices were maintained in standard aCSF at 22–23 °C for at least 1 h, until they were individually transferred to a submerged recording chamber.

Brain slice preparation was done in a similar way for mice and human tissue. Mice were deeply anesthetized by isoflurane 1.5-3.0%. After decapitation, brains submerged in (~4°C) cutting solution that was continuously bubbled with 95% O_2-5% CO_2 containing (in mM) sucrose 248, KCl 2, $MgSO_4.7H_2O$ 3, $CaCl_2.2H_2O$ 1, $NaHCO_3$ 26, $NaH_2PO_4.H_2O$ 1.25, D-glucose 10. Mouse somatosensory cortical slices (350 μm) were prepared in the coronal plane similar to human slice preparation as described above.

A subset of cortical slices in both human and mouse were prepared using N-Methyl-D-glucamine (NMDG) protective recovery method [19]. The cortical tissue blocks were transferred and sectioned in 2–4 °C NMDG-HEPES aCSF solution containing (in mM): NMDG 92, KCl 2.5, NaH_2PO_4 1.25, $NaHCO_3$ 30, HEPES 20, Glucose 25, Thiourea 2, Na L-ascorbate 5, Na-Pyruvate 3, $CaCl_2.4H_2O$ 0.5, and $MgSO_4.7H_2O$. The pH of NMDG-HEPES aCSF solution was adjusted to 7.3–7.4 using hydrochloric acid and the osmolarity was 300–305 mOsm. The cortical slices were prepared using vibratome as described above. After slicing, slices were transferred to a recovery chamber filled with 32–34 °C NMDG-HEPES aCSF solution which continuously bubbled with 95% O_2–5% CO_2. After 12 min, the slices were transferred to an incubation solution-HEPES aCSF-containing (in mM): NaCl 92, KCl 2.5, NaH_2PO4.H_2O 1.25, $NaHCO_3$ 30,

HEPES 20, Glucose 25, Thiourea 2, Na L-ascorbate 5, Na-Pyruvate 3, $CaCl_2.4H_2O$ 2, and $MgSO_4.7H_2O$ 2. After 1-h incubation at room temperature, slices were transferred to a recording chamber and continuously perfused with aCSF containing (in mM): NaCl 126, KCl 2.5, $NaH_2PO_4.H_2O$ 1.25, $NaHCO_3$ 26, Glucose 12.6, $CaCl_2.2H_2O$ 2, and $MgSO_4.7H_20$ [6].

Whole-cell patch-clamp recording from human and mice cortical slices

For electrophysiological recordings, cortical slices were placed in a recording chamber mounted on a fixed-stage upright microscope (Axioskop 2 FS MOT; Carl Zeiss, Germany). Slices were continuously perfused with carbogenated (95% O_2/5% CO_2) aCSF containing of (in mM): NaCl 123, KCl 4, $CaCl_2.2H_2O$ 1.5, $MgSO_4.7H_2O$ 1.3, $NaHCO_3$ 26, $NaH_2PO4.H_2O$ 1.2, and D-glucose 10, pH 7.40 at 32-34 °C. Cortical neurons were visualized using an IR-CCD camera (IR-1000, MTI, USA) with a 40x water immersion objective. Patch pipettes (3-6 MΩ) were pulled from standard borosilicate glass pipettes (thin-wall borosilicate tubes with filaments, World Precision Instruments, Sarasota, FL, USA) using a vertical puller (PC-10, Narishige). For somatic recording of electrophysiological properties, patch pipettes were filled with intracellular solution containing (in mM): K-gluconate 135; NaCl 10; HEPES 10; $MgCl_2$ 1; Na_2ATP 2; GTP 0.3, pH adjusted with KOH to 7.4 (290–309 mOsm). A subset of data were collected with excitatory (APV 50 µM, Sigma; CNQX 25 µM, Sigma) and inhibitory (Bicuculline 10 µM, Sigma; CGP-35348 10 µM, Sigma) synaptic activity blocked.

Electrical signals were measured with Multiclamp 700A amplifier, Axopatch 200B amplifier, pClamp 9.2 and pClamp 10.6 data acquisition software (Axon instruments, Molecular Devices, USA). Subsequently, electrical signals were digitized at 20 kHz using a 1320X digitizer and 1440A digitizer (Axon instruments, Molecular Devices, USA). The access resistance was monitored throughout the recording (typically between 8-20 MΩ), and neurons were discarded if the access resistance was > 25 MΩ.

Axon Binary Format to Neurodata Without Borders file conversion

The x-to-nwb repository was used to convert current clamp recordings in axon binary format (ABF) to Neurodata Without Borders (NWB) format. Separate converters were used for files recorded using pClamp 9.0, which output ABFv1 files, and pClamp >10.0, which output ABFv2 files, to ensure valid conversions while incorporating the essential metadata. The key aspects of our usage of these data conversion computer scripts relate to defining which ABF channels correspond to stimulus and response traces and ensuring that appropriate scale and offset factors are applied properly upon conversion. We incorporated the ndx-dandi-icephys metadata extensions to allow for inclusion of user-defined 'Subject' and 'Lab' metadata fields to be able to include specific metadata including 'subject_id', 'age', 'species', 'cell_id' and 'tissue_sample_id'.

Relevant metadata was recorded in two separate tables: first, patient level information, including demographics and clinical information; and second, recording specific information, which relate to aspects of each individual cell's recording, such as channels corresponding to stimulus, response, and resting membrane potential. The patient level demographics table included fields including 'Resection date', 'Resection procedure', 'Sex', 'Age', 'Years of seizure history', 'Diagnosis', ' Seizure type', 'Presence of a tumor', 'Antiepileptic drugs'. Recording specific metadata includes experiment 'date', 'Cell number' to differentiate recordings from distinct cells taken on the same day, 'Cell layer', 'Gain', 'Offset', 'Response channel', 'Command channel' and 'RMP' to record the resting membrane potential at the initial time of recording. Additional recording metadata was extracted directly from ABF files using custom scripts to extract the stimulus start and end times and the stimulus sampling rate.

Electrophysiology feature extraction

The Intrinsic Physiology Feature Extractor (IPFX) toolbox was used to extract features from converted NWB files [3], [20]. All experiments consisted of long-square hyperpolarizing and depolarizing current injections, and extracted features include subthreshold features (i.e., input resistance, sag ratio), action potential properties (i.e., action potential half-width, threshold time and voltage) derived from the rheobase spike as well as multi-action potential spike train features derived from the IPFX-defined “hero” sweep (i.e., adaptation index), as described previously [6]. Our included metadata files contain stimulus start and end times along with an IPFX-compatible stimulus description ontology file for reproducibility and to facilitate the feature extraction process.

Results

In Table 1, we summarize the three main axes differentiating the cells and recordings in this dataset. Namely, recordings differed by species (human versus mouse), by cortical layer of the cell body of the recorded neuron (Layer 2&3, Layer 3c, and Layer 5), and whether synaptic blockers were used in the external recording solution. Putative interneurons were identified by their action potential characteristics (large maximal firing rates and typically large spike after-hyperpolarization amplitudes) as described in Chameh, et al 2021. One reason why synaptic blockers were used is to make a subset of recordings more consistent with protocols used in other labs, such as the Allen Institute for Brain Sciences [3], [11].

Dataset Overall	# cells	# individuals	Number of Pyr	Number of ints	L2&3	L3c	L5

Type			cells				
Human (aCSF)	86	19	85	1	27	15	44 (1 int)
Human (aCSF containing synaptic blockers)	43	17	40	3	16 (2 int)	5 (1 int)	22
Mouse (aCSF containing synaptic blockers)	12	5	11	1	0	0	12 (1 int)

Table 1: Summary of number of electrophysiological recordings across major experimental conditions

In current clamp mode, hyperpolarizing and depolarizing current injections (600-1000 ms) were used to characterize biophysical features of cortical neurons, with examples from three recorded cells shown in Fig. 2.

Figure 2: Example voltage traces from 3 separate L5 neurons, a human L5 neuron recorded in standard aCSF (top), a human L5 neuron recorded in aCSF with synaptic blockers (2nd row),

and from a mouse L5 neuron recorded in aCSF with synaptic blockers (third row). Bottom row shows the hyperpolarizing and depolarizing injected step currents that were applied to each neuron and includes the most hyperpolarizing current injection (blue), the rheobase (orange) and most depolarizing current injection (green).

In Fig. 3A, we highlight how the use of synaptic blockers in the external solution affects recorded subthreshold neuronal properties. Specifically, among recorded human L5 neurons, applying synaptic blockers significantly increased the recorded input resistance (standard aCSF: M=81.7 MΩ, SD=35.9, n = 44; aCSF containing synaptic blockers: M=214 MΩ, SD=102, n = 22; $p < 4.15e-06$). However, in Fig. 3B we demonstrate that the inclusion of synaptic blockers does not have a noticeable effect on the width of the action potentials elicited in response to supratheshold stimuli. To illustrate comparisons across species, in Fig. 3C and Fig. 3D, we show distributions of the sag ratio and input resistance respectively recorded from neurons in both human and mouse cortical L5 neurons (in the presence of synaptic blockers).

Figure 3: Distributions of input resistance, AP width and sag ratio measured in L5 neurons across experimental conditions. Inclusion of synaptic blockers in aCSF has an observable effect on input resistance (A) but not AP width (B) as measured in human L5 neurons. Input resistance (C) and sag ratio (D) are comparable in L5 neurons across mouse and human when both are recorded in the presence of synaptic blockers in the external solution.

To compare the effect of solution used for the brain slice preparation on intrinsic properties, we compared the input resistance and sag ratio recorded following preparation in either solution. Due to the confounding of cutting or slice preparation solution used and cortical layer resected we highlight recordings using synaptic blockers in aCSF recording solution from L2/3 and L5 of

human cortex. In Fig. 4A, we highlight a consistent trend of higher measured input resistance in the recordings made following preparation in the NMDG recovery solution compared to the sucrose solution and note that the comparison in L5 differed significantly (L2/3 NMDG M=253, SD=95.3, n=10; L2/3 sucrose: M=156, SD=68, n=5; L5 NMDG M=348, SD=101, n=4; L5 sucrose: M=185, SD=76.8, n=18, Mann-Whitney U test, $p < 0.05$). In Fig. 4B, we compare the sag ratio across the same conditions (L2/3 NMDG M=0.0877, SD=0.0546, n=10; L2/3 sucrose: M=0.0781, SD=0.0326, n=5; L5 NMDG M=0.158, SD=0.0770, n=4; L5 sucrose: M=0.0935, SD=0.0349, n=18). These comparisons emphasize the importance of the conditions used for the experimental preparation (see Discussion).

Figure 4: Distributions of input resistance and sag ratio measured in L2/3 and L5 neurons derived from different slice preparations. Use of NMDG in the solution used for preparation of brain slices has an observable effect on input resistance as measured in human L2/3 and L5 neurons (A). Comparison of sag ratio measured in sucrose and NMDG cutting solution across human L2/3 and L5 neurons (B). All illustrated recordings performed using synaptic blockers in aCSF recording solution.

To illustrate the rich diversity of the metadata for each of the human recordings, in Fig. 5 we highlight specific comparisons of input resistance and sag ratio measurements recorded in regular aCSF across demographic conditions. Specifically, we focus on the input resistance as a fundamental passive electrophysiological property and the sag ratio as an active property that has previously been used to distinguish between subtypes of human neurons [6], [11]. In Fig. 5B, 5E we compare distributions of these electrophysiological features measured in male and female patients (Fig. 5B, E); whether the recorded cell was resected from patients with a tumor or not (Fig. 5 C, F); and across the three different brain lobes from which neuronal tissue was resected (Fig. 5A, D). Note that all cells from frontal and parietal cortex were

recorded from tissue resected near the site of the epileptogenic focus, whereas all cells from temporal cortex were recorded distal from the epileptogenic focus (with the exception of 1 subject).

Figure 5: Distributions of input resistance (A, B, C) and sag ratio (D, E, F) recorded in human L5 neurons with standard aCSF. Measurements are grouped and compared by major brain lobe of resection location (A, D); sex (B, E); and by clinical diagnosis or reason for brain surgery (note that in most cases, tissue used for recordings is not at site of the epileptogenesis or tumor) (C, F). Note that most of these patients with tumors also have epilepsy (they are indicated as having 'tumor').

Additionally, we illustrate the relationship of the input resistance and sag ratio against both patient age at time of surgical resection (Fig. 6A, B) and years of seizure experienced by the patient prior to the surgical intervention (Fig. 6C, D).

Figure 6: Scatterplots of input resistance and sag ratio vs patient age (A, B) and duration of seizure prior to surgical intervention (C, D) recorded in human L5 neurons with standard aCSF.

Code Availability

Conversion and analysis scripts are available at:
https://github.com/derekhoward/nwb_conversion

Data Availability

Data is available on the DANDI platform at the following links:

Mouse data: https://gui.dandiarchive.org/#/dandiset/000172

Human data: https://gui.dandiarchive.org/#/dandiset/000211

Other associated metadata for recordings can be found at the following link:

https://github.com/derekhoward/nwb_conversion/blob/master/data/processed/meta/all_meta_cleaned.csv

Application Scenarios

The recordings in this database permit the quantification of biophysical properties from a diverse set of neurons, including human and mouse neurons with a well described set of metadata. Independent variables collected include age, sex, seizure history and cortical layer from which the tissue was resected. Additionally, experiments on the human neurons were performed with the use of synaptic blockers and without, allowing for comparisons and integration with other intrinsic electrophysiological databases comprising patch-clamp recordings, including from the the Allen Cell types database (https://celltypes.brain-map.org/).

These data from current-clamp experiments are particularly beneficial for the development of conductance-based models of human neurons [11], [21], [22]. In particular, we highlight that in some instances, it may be more suitable to constrain biophysical models to human data in the presence of synaptic blockers, that is to say, when background synaptic activity is having a significant effect on input resistance measurements. The voltage responses can be used as a training set to constrain biophysical models when combined and integrated with other publicly available databases that provide relevant morphologies and channel kinetics, such as NeuroMorpho.org, Channelpedia or ICGenealogy [23]–[25]. Moreover, fitting biophysical models to data that is grouped based on demographic information can allow for cross-group

comparisons using in silico approaches. Usage of these models in neuronal or circuit simulations can thus help to further predict and unveil the potential effects of differences in neuronal physiology across demographic groupings [26], [27].

Discussion and Limitations

The repository provided is focused mainly on cortical neurons derived from human tissue. There are comparatively fewer recordings for analyses of mouse neuron function provided and all of these were performed using synaptic blockers that were shown to have a baseline effect on input resistance.

The recordings from human specimens derived from tissue during the surgical resection of diseased tissue for patients with intractable epilepsy or brain tumors. Along with having suffered seizures for an extended period of time, the patients may have concurrently taken one or a variety of anti-epileptic drugs that could have affected baseline neuronal excitability characteristics.

While these data were collected for the purpose of characterizing intrinsic properties of human neocortical neurons, we note that they were collected using different sets of experimental conditions, including those related to different recording solutions as well as cutting solutions. Our analyses suggest such experimental condition differences likely contribute to differences in downstream electrophysiological properties, such as input resistances being higher among cells recorded in the presence of synaptic blockers of excitatory and inhibitory neurotransmission. The observed effect of synaptic blockers on the input resistance is due to reduction of overall membrane permeability as a consequence of the block of both excitatory and inhibitory conductances but does not distinguish whether they have equal effect [28]. In contrast, the

comparisons of electrophysiological measures following different cutting solutions highlight potential effects on neuronal excitability. We observe variability in the measured input resistance but consider that these effects may be due to changes in conductivity across the membrane or also experimental biases in selection of healthy neurons for patch-clamp protocol due to differential response to solutions of different osmolarity. Furthermore, there exists contrasting results in the literature on the effects of NMDG on neuronal excitability and synaptic transmission which shows the context-dependence of the many experimental variables [29], [30]. Taken together, such potential differences in electrophysiological characteristics due to experimental conditions are important to consider when re-using these data in downstream analyses.

Declarations

Acknowledgements

We are grateful to our neurosurgical patients and their families for consenting to the use of their tissue samples for research. We thank Dr. Gelareh Zadeh and Dr. Mark Bernstein for their assistance in obtaining brain tissue samples and Victoria Barkley and Marjan Rafiee for assistance in compiling demographic and chart information. We thank Sara Mahallati and Iliya Weisspapir for assistance in tissue preparation. We thank Wataru Inoue and Michael Feyerabend for their critical comments on the manuscript. We acknowledge generous support from the Centre for Addiction and Mental Health Discovery Fund, Krembil Brain Institute Fund, National Institute of Health, and Kavli Foundations.

Competing interests

The authors declare no competing interests.

References

[1] S. J. Tripathy, J. Savitskaya, S. D. Burton, N. N. Urban, and R. C. Gerkin, “NeuroElectro: a window to the world’s neuron electrophysiology data,” *Front. Neuroinform.*, vol. 8, p. 40, Apr. 2014.

[2] S. J. Tripathy, S. D. Burton, M. Geramita, R. C. Gerkin, and N. N. Urban, “Brain-wide analysis of electrophysiological diversity yields novel categorization of mammalian neuron types,” *J. Neurophysiol.*, vol. 113, no. 10, pp. 3474–3489, Jun. 2015.

[3] N. W. Gouwens *et al.*, “Classification of electrophysiological and morphological neuron types in the mouse visual cortex,” *Nat. Neurosci.*, vol. 22, no. 7, pp. 1182–1195, Jul. 2019.

[4] H. Markram *et al.*, “Reconstruction and Simulation of Neocortical Microcircuitry,” *Cell*, vol. 163, no. 2, pp. 456–492, Oct. 2015.

[5] H. Mohan *et al.*, “Dendritic and Axonal Architecture of Individual Pyramidal Neurons across Layers of Adult Human Neocortex,” *Cereb. Cortex*, vol. 25, no. 12, pp. 4839–4853, Dec. 2015.

[6] H. M. Chameh *et al.*, “Diversity amongst human cortical pyramidal neurons revealed via their sag currents and frequency preferences,” *Nat. Commun.*, vol. 12, no. 1, pp. 1–15, 2021.

[7] J. Berg *et al.*, “Human neocortical expansion involves glutamatergic neuron diversification,” *Nature*, vol. 598, no. 7879, pp. 151–158, Oct. 2021.

[8] A. Gidon *et al.*, “Dendritic action potentials and computation in human layer 2/3 cortical neurons,” *Science*, vol. 367, no. 6473, pp. 83–87, Jan. 2020.

[9] L. Beaulieu-Laroche *et al.*, “Enhanced Dendritic Compartmentalization in Human Cortical

Neurons,” *Cell*, vol. 175, no. 3, pp. 643–651.e14, Oct. 2018.

[10] L. Beaulieu-Laroche *et al.*, “Allometric rules for mammalian cortical layer 5 neuron biophysics,” *Nature*, vol. 600, no. 7888, pp. 274–278, Dec. 2021.

[11] B. E. Kalmbach *et al.*, “h-Channels Contribute to Divergent Intrinsic Membrane Properties of Supragranular Pyramidal Neurons in Human versus Mouse Cerebral Cortex,” *Neuron*, vol. 100, no. 5, pp. 1194–1208.e5, Dec. 2018.

[12] B. E. Kalmbach *et al.*, “Signature morpho-electric, transcriptomic, and dendritic properties of human layer 5 neocortical pyramidal neurons,” *Neuron*, vol. 109, no. 18. pp. 2914–2927.e5, 2021 [Online]. Available: http://dx.doi.org/10.1016/j.neuron.2021.08.030

[13] R. D. Hodge *et al.*, “Conserved cell types with divergent features in human versus mouse cortex,” *Nature*, vol. 573, no. 7772, pp. 61–68, Sep. 2019.

[14] Y. Deitcher *et al.*, “Comprehensive Morpho-Electrotonic Analysis Shows 2 Distinct Classes of L2 and L3 Pyramidal Neurons in Human Temporal Cortex,” *Cereb. Cortex*, vol. 27, no. 11, pp. 5398–5414, Nov. 2017.

[15] C. M. Florez *et al.*, “In vitro recordings of human neocortical oscillations,” *Cereb. Cortex*, vol. 25, no. 3, pp. 578–597, Mar. 2015.

[16] A. Mansouri, A. Fallah, and T. A. Valiante, “Determining surgical candidacy in temporal lobe epilepsy,” *Epilepsy Res. Treat.*, vol. 2012, p. 706917, Feb. 2012.

[17] G. Kostopoulos, C. Drapeau, M. Avoli, A. Olivier, and J. G. Villemeure, “Endogenous adenosine can reduce epileptiform activity in the human epileptogenic cortex maintained in vitro,” *Neurosci. Lett.*, vol. 106, no. 1–2, pp. 119–124, Nov. 1989.

[18] R. Köhling and M. Avoli, “Methodological approaches to exploring epileptic disorders in the human brain in vitro,” *J. Neurosci. Methods*, vol. 155, no. 1, pp. 1–19, Jul. 2006.

[19] J. T. Ting *et al.*, “Preparation of Acute Brain Slices Using an Optimized N-Methyl-D-glucamine Protective Recovery Method,” *J. Vis. Exp.*, no. 132, Feb. 2018, doi: 10.3791/53825. [Online]. Available: http://dx.doi.org/10.3791/53825

[20] B. R. Lee *et al.*, “Scaled, high fidelity electrophysiological, morphological, and transcriptomic cell characterization,” *Elife*, vol. 10, Aug. 2021, doi: 10.7554/eLife.65482. [Online]. Available: http://dx.doi.org/10.7554/eLife.65482

[21] G. Eyal *et al.*, “Unique membrane properties and enhanced signal processing in human neocortical neurons,” *Elife*, vol. 5, Oct. 2016, doi: 10.7554/eLife.16553. [Online]. Available: http://dx.doi.org/10.7554/eLife.16553

[22] H. K. Yao *et al.*, “Reduced inhibition in depression impairs stimulus processing in human cortical microcircuits,” *Cell Rep.*, vol. 38, no. 2, p. 110232, Jan. 2022.

[23] G. A. Ascoli, D. E. Donohue, and M. Halavi, “NeuroMorpho.Org: a central resource for neuronal morphologies,” *J. Neurosci.*, vol. 27, no. 35, pp. 9247–9251, Aug. 2007.

[24] R. Ranjan *et al.*, “Channelpedia: an integrative and interactive database for ion channels,” *Front. Neuroinform.*, vol. 5, p. 36, Dec. 2011.

[25] W. F. Podlaski, A. Seeholzer, L. N. Groschner, G. Miesenböck, R. Ranjan, and T. P. Vogels, “Mapping the function of neuronal ion channels in model and experiment,” *eLife*, vol. 6. 2017 [Online]. Available: http://dx.doi.org/10.7554/elife.22152

[26] N. A. Goriounova *et al.*, “Large and fast human pyramidal neurons associate with intelligence,” *Elife*, vol. 7, Dec. 2018, doi: 10.7554/eLife.41714. [Online]. Available: http://dx.doi.org/10.7554/eLife.41714

[27] A. Guet-McCreight *et al.*, “Age-dependent increased sag current in human pyramidal neurons dampens baseline cortical activity,” *bioRxiv*, p. 2021.11.03.467014, Jan. 12, 2022 [Online]. Available: https://www.biorxiv.org/content/biorxiv/early/2022/01/12/2021.11.03.467014. [Accessed: Mar. 08, 2022]

[28] P. A. Núñez-Abades, J. M. Pattillo, T. M. Hodgson, and W. E. Cameron, “Role of synaptic inputs in determining input resistance of developing brain stem motoneurons,” *J. Neurophysiol.*, vol. 84, no. 5, pp. 2317–2329, Nov. 2000.

[29] J. B. Thuma and S. L. Hooper, “Choline and NMDG directly reduce outward currents: reduced outward current when these substances replace Na+ is alone not evidence of Na+-activated K+ currents,” *J. Neurophysiol.*, vol. 120, no. 6, pp. 3217–3233, Dec. 2018.

[30] E. M. Avegno, J. W. Middleton, and N. W. Gilpin, “Synaptic GABAergic transmission in the central amygdala (CeA) of rats depends on slice preparation and recording conditions,” *Physiol Rep*, vol. 7, no. 19, p. e14245, Oct. 2019.

Figure 1

Click here to access/download;Figure;Figure1.png

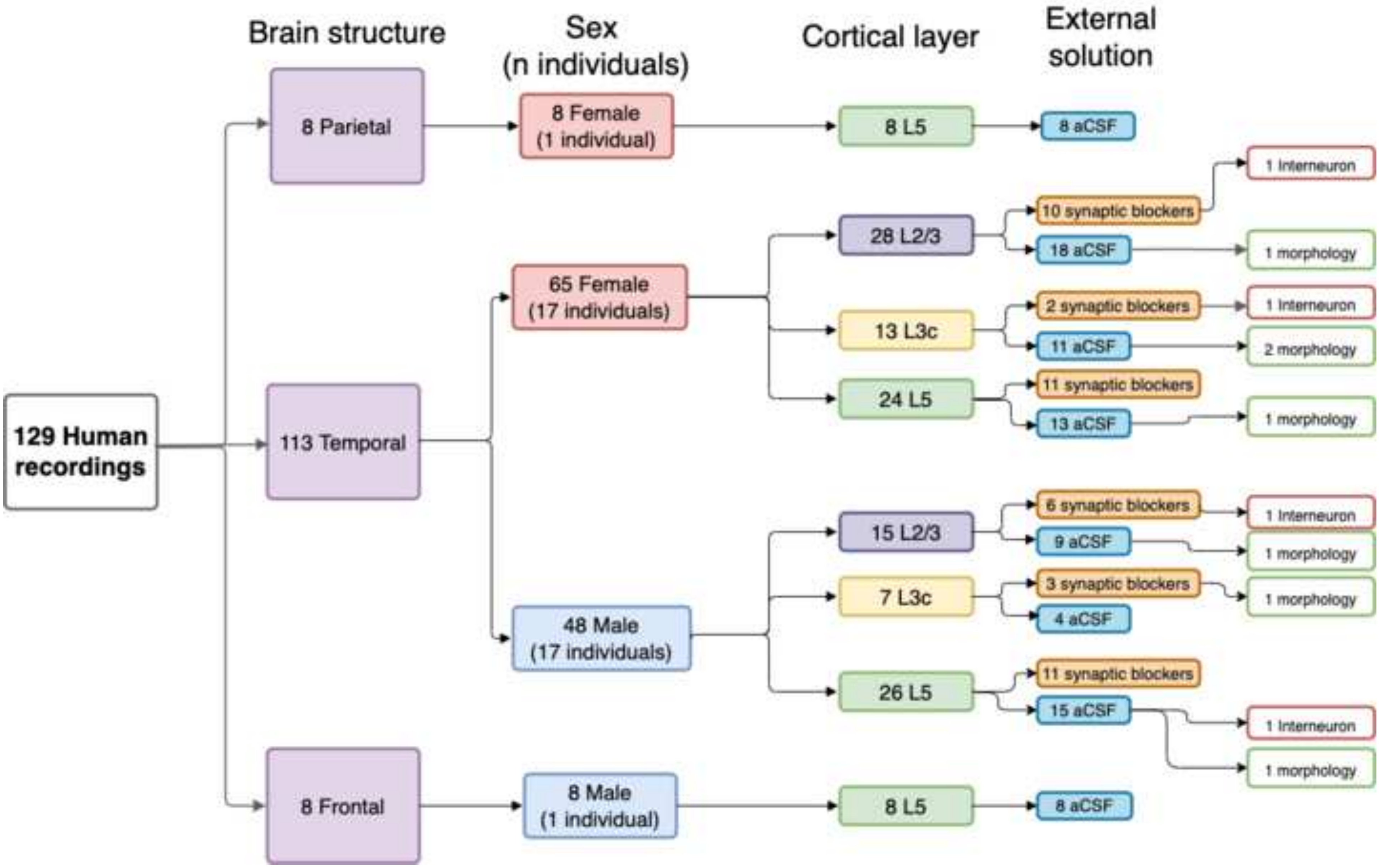

Figure 2 Click here to access/download;Figure;Figure2.png

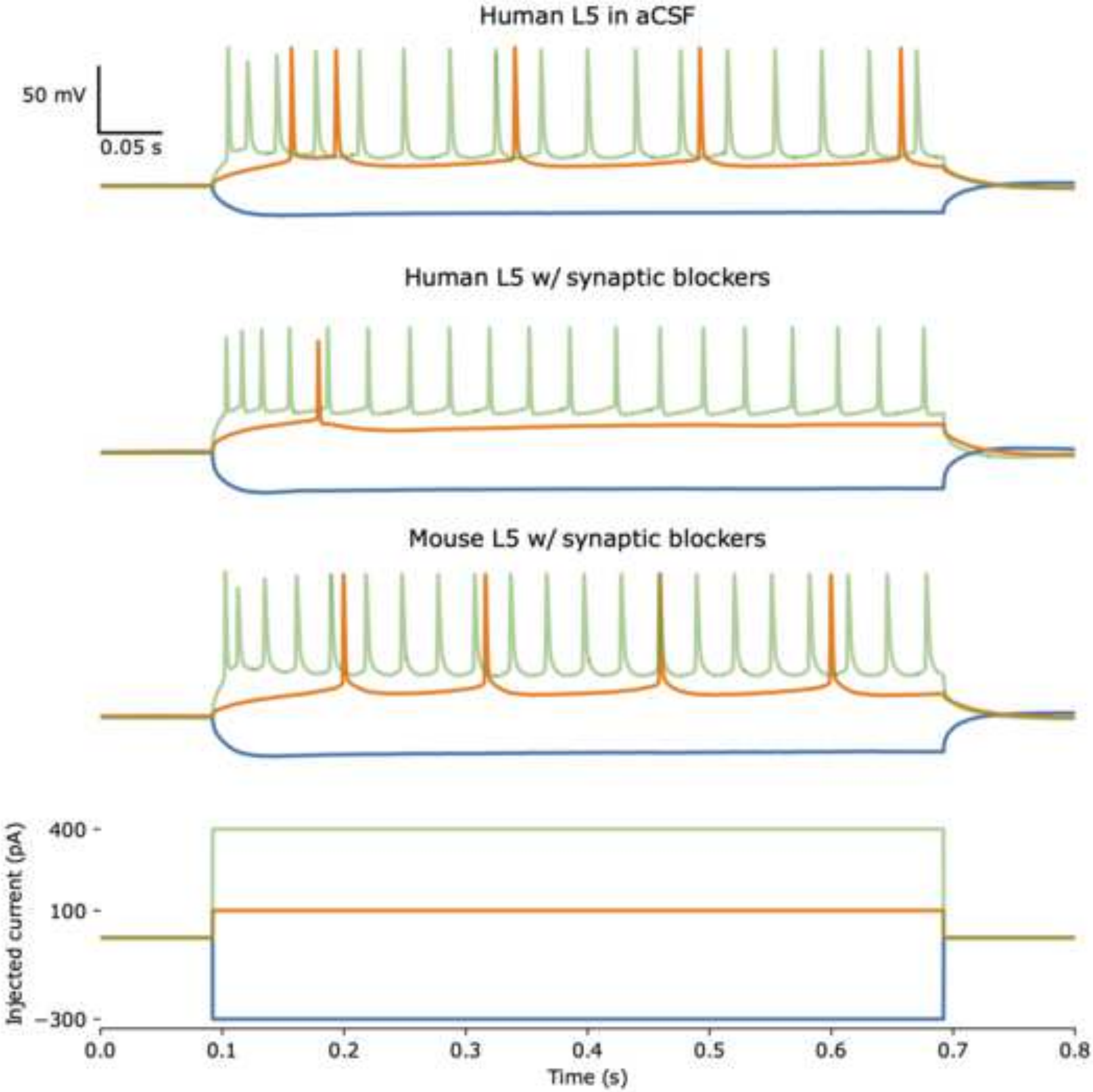

Figure 3

Click here to access/download;Figure;Figure3.png

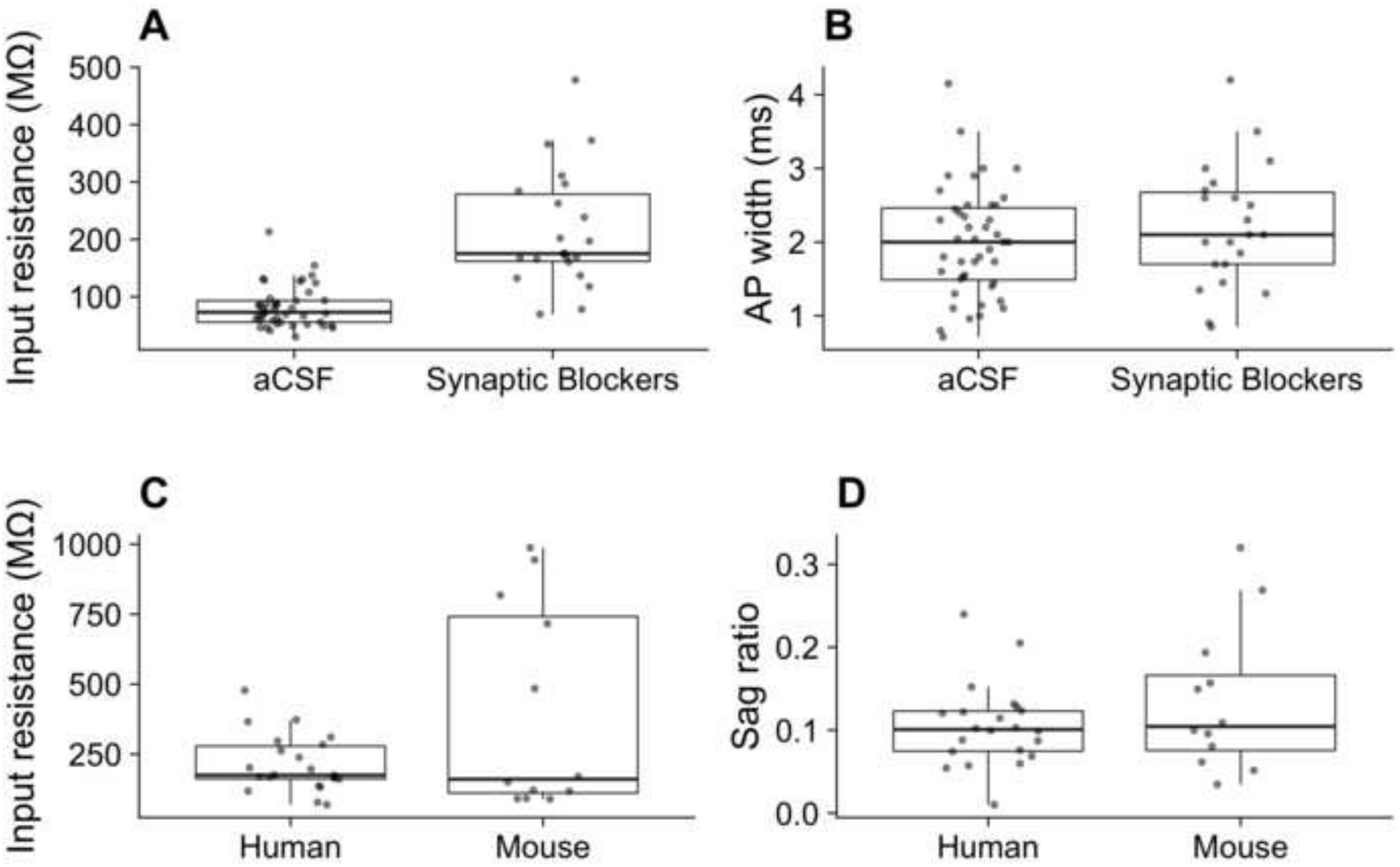

Figure 4

Click here to access/download;Figure;Figure4.png

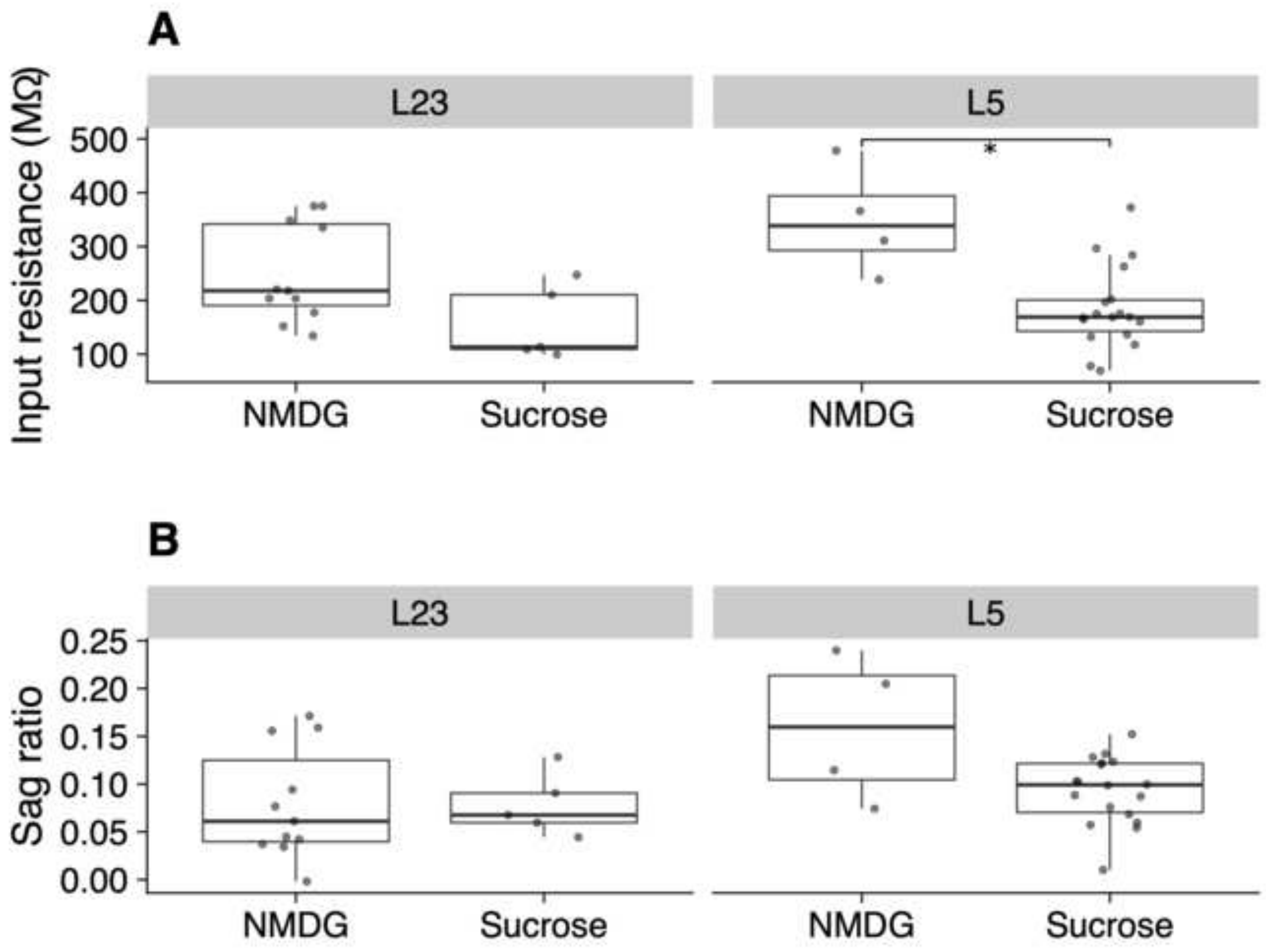

Figure 5

Click here to access/download;Figure;Figure5.png

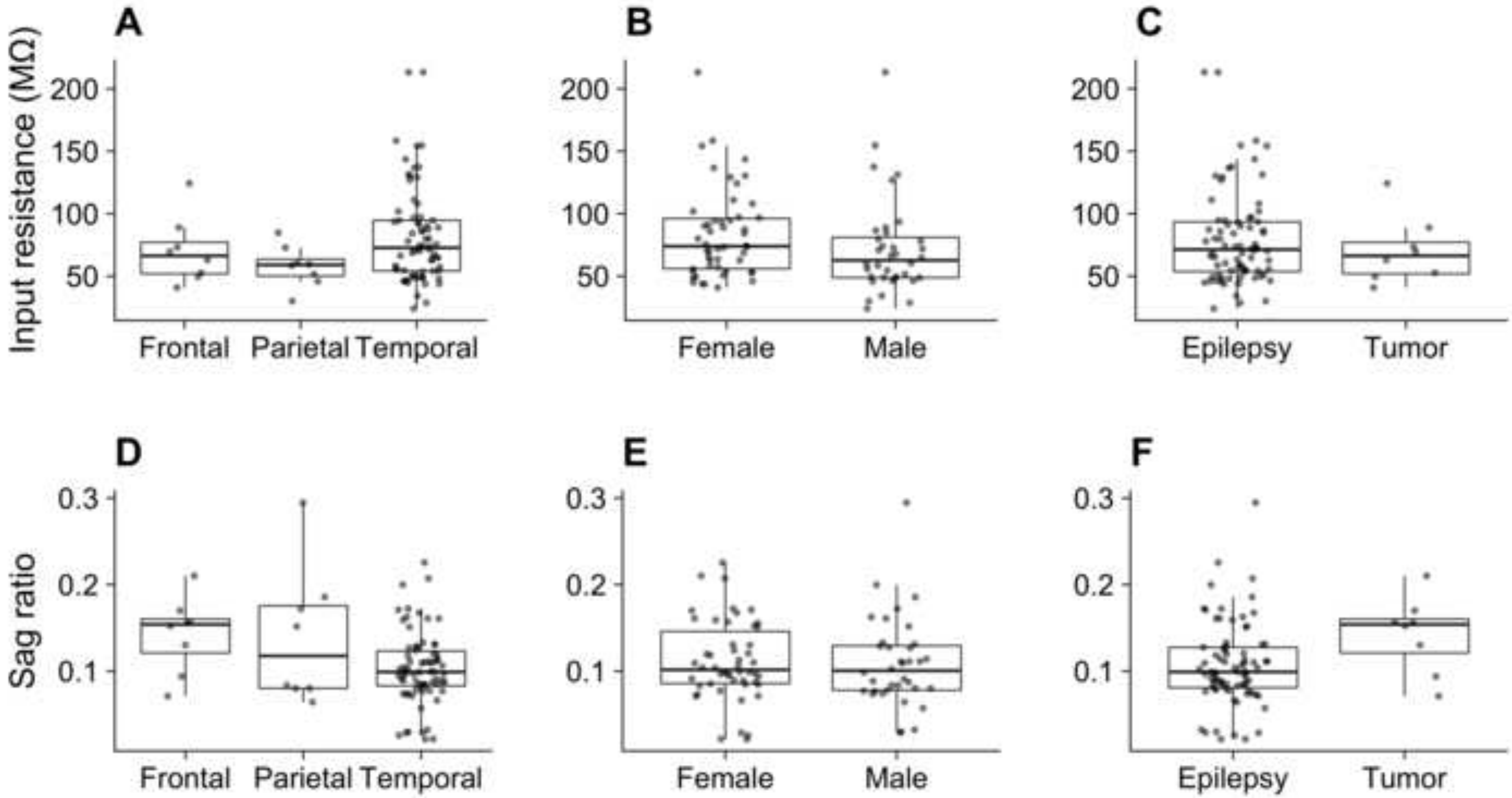

Figure 6

Click here to access/download;Figure;Figure6.png

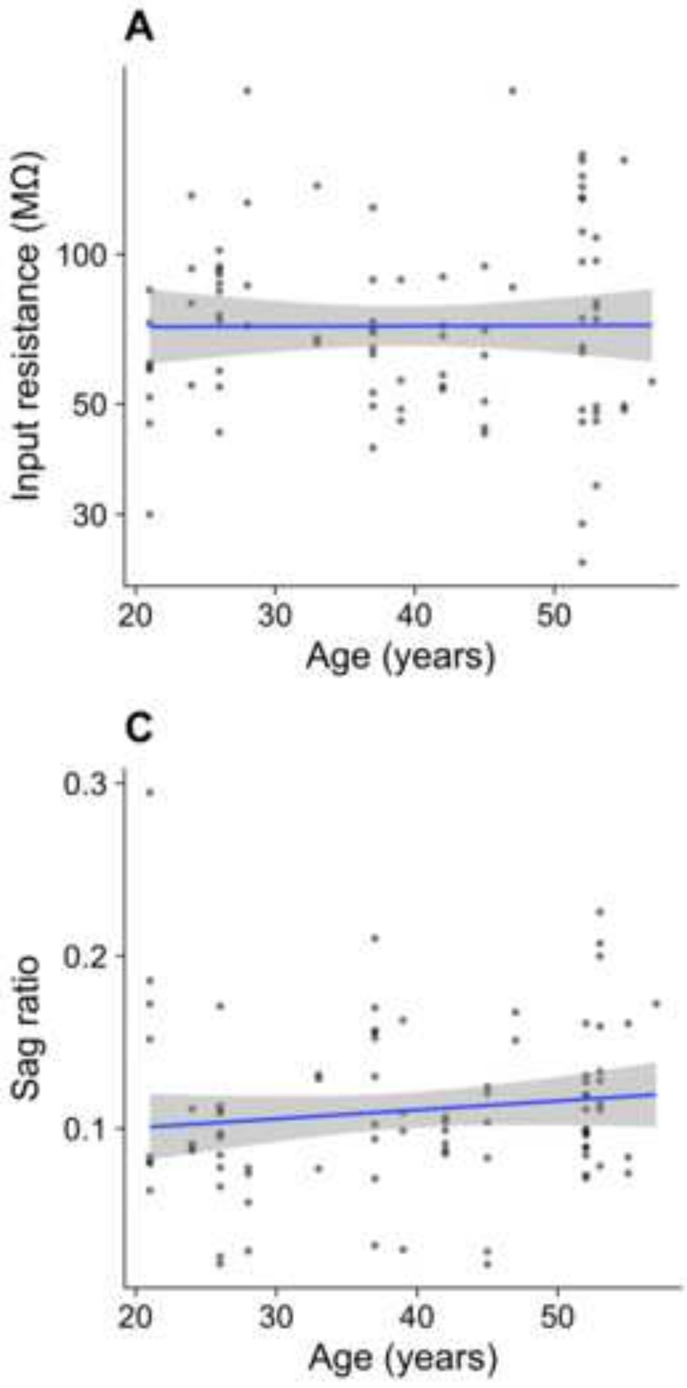

Supplement: giac108_GIGA-D-22-00068_Original_Submission [file giac108_giga-d-22-00068_original_submission.pdf]
