# Supplementary material for: An in vitro whole-cell electrophysiology dataset of human cortical neurons
Source: Gigascience. 2022 Nov 15;11:giac108. doi: 10.1093/gigascience/giac108 (PMC9664072; doi:10.1093/gigascience/giac108)

GigaScience

An in vitro whole-cell electrophysiology dataset of human cortical neurons

--Manuscript Draft--

**Manuscript Number:**	GIGA-D-22-00068R1
**Full Title:**	An in vitro whole-cell electrophysiology dataset of human cortical neurons
**Article Type:**	Data Note
**Funding Information:**	Centre for Addiction and Mental Health (Discovery Fund) — Dr Shreejoy Tripathy<br>Krembil Brain Institute Fund — Dr Taufik A Valiante<br>National Institute of Health — Dr Taufik A Valiante<br>Kavli Foundation — Dr Shreejoy Tripathy

Abstract:

Background

Whole-cell patch-clamp electrophysiology is an essential technique for understanding how single neurons translate their diverse inputs into a functional output. The relative inaccessibility of live human cortical neurons for experimental manipulation has made it difficult to determine the unique features of how human cortical neurons differ from their counterparts in other species.

Findings

We present a curated repository of whole-cell patch-clamp recordings from surgically resected human cortical tissue, encompassing 118 neurons from 35 individuals (age ranging 21-59 years old; 17 male, 18 female). Recorded human cortical neurons derive from layers 2&3 (L2&3), deep layer 3 (L3c) or layer 5 (L5) and are annotated with a rich set of subject and experimental metadata. For comparison, we also provide a limited set of comparable recordings from 21-day old mice (11 cells from 5 mice). All electrophysiological recordings are provided in the Neurodata Without Borders (NWB) format and are available for further analysis via the Distributed Archives for Neurophysiology Data Integration (DANDI) online repository. The associated data conversion code is made publicly available and can help others in converting electrophysiology datasets to the open NWB standard for general re-use.

Conclusion

These data can be used for novel analyses of biophysical characteristics of human cortical neurons including in cross-species or cross-lab comparisons or in building computational models of individual human neurons.

**Corresponding Author:**	Derek Howard<br>CAMH: Centre for Addiction and Mental Health<br>Toronto, Ontario CANADA
**Corresponding Author Secondary Information:**	
**Corresponding Author's Institution:**	CAMH: Centre for Addiction and Mental Health
**Corresponding Author's Secondary Institution:**	
**First Author:**	Derek Howard
**First Author Secondary Information:**	
**Order of Authors:**	Derek Howard
	Homeira Moradi Chameh
	Alexandre Guet-McCreight
	Huan Allen Hsiao

Powered by Editorial Manager® and ProduXion Manager® from Aries Systems Corporation

	Maggie Vuong
	Young Seok Seo
	Prajay Shah
	Anukrati Nigam
	Yuxiao Chen
	Melanie Davie
	Etay Hay
	Taufik A Valiante
	Shreejoy Tripathy
**Order of Authors Secondary Information:**	
**Response to Reviewers:**	Reviewer #1:<br>In this work, Howard et al. present a dataset of electrophysiological recordings from human cortical tissue supplemented with recordings taken from mouse somatosensory cortex. Making such a dataset publicly available is a useful addition to the field and the data structure is clearly presented in Figure 1. The methods are appropriate and the manuscript overall is well-written. I am supportive of accepting this dataset as a Data Note, but also think there is room for improvement.<br><br>Major points:<br>1) I selected a few random recordings and most of the recordings seem to be of reasonable quality but there are some of which I wonder if they need to be included. For example:<br>2016_01_28_0012: shows spontaneous spikes and spike saturation towards later sweeps<br>2018_02_08_0001: the spikes in this recording appear a bit strange in shape and the trace appears noisier than other recordings. Was the access for this cell okay? Also the bridge balancing seems to be a bit off. Was bridge balancing applied for all recordings? Please describe this in the Methods section.<br>2016_02_04_0042: this cell has a resting membrane potential of -45 mV, which in my experience is not a sign of a healthy cell. I'm not familiar with human cells but I assume this is true across species.<br>2016_02_04_0015: There's a sudden jump in the resting membrane potential in this cell (sweep 11 - 12), also indicating a problem with the health of the cell/the recording<br><br>- I understand that human tissue is not trivial to come by and that the authors want to include as many of their recordings as possible, but I think the quality of the dataset could be improved by the application of more stringent selection criteria.<br><br>We thank the reviewer for their overall positive impressions of this work and attention to detail in reviewing the submitted dataset and traces from example cells.<br><br>Following careful consideration of the comments from both reviewers, we have taken steps to update and clarify the selection criteria for the cells and traces that we have made available. Most prominently, we have taken this reviewer's suggestion to use voltage deviation from baseline as a useful metric for excluding unhealthy cells or potentially problematic recordings (see details below). Now, we have excluded XXX cells, with YY human and ZZ mouse cell recordings remaining. In addition, we note that we did not explicitly correct for the bridge balance and have added this detail to the methods section.<br><br>In the updated Methods section, we have now included an additional section describing our Quality Control process that outlines our cell selection criteria (pasted below):<br><br>We performed both automated and manual quality control checks of converted recordings to ensure dataset quality and maximize reuse potential. Using IPFX, we checked whether the “v_baseline” feature of any current sweep in a recording deviated by more than 20% from the initial measure in the first depolarizing step. Any recordings

that had any sweep deviate beyond the 20% threshold were not included in the dataset. Also, individual converted recordings were visualized at 3 injected current steps (the most hyperpolarizing pulse, the rheobase and the most depolarizing step), along with the recording's frequency/input curve to identify any abnormal responses and for identification of interneuron cell types. We note that in some instances, we observed spike saturation at higher steps of current injection as well as spontaneous spikes outside of the window of current step injection.
We performed both automated and manual quality control checks of converted recordings to ensure dataset quality and maximize reuse potential. Using features automatically extracted via IPFX, we checked whether the baseline voltage of a sweep (i.e., v_baseline) deviated by more than 10mV from the initial measure in the first current injection step. Any cell recordings that had any sweep deviate beyond the 10mV threshold were not included in the final contributed dataset. We also included the measures for maximum drift of baseline Vm in each recording's metadata under the field max_drift_Vm.
Also, individual recordings were manually inspected at 3 injected current steps (the most hyperpolarizing pulse, the rheobase and the most depolarizing step). In addition, we further manually inspected each neuron recording's frequency/input curve to identify any abnormal responses, and also, to identify putative recordings from interneurons.
Following this manual inspection process, we note that in some instances, we observed some evidence for spike saturation at higher steps of current injection. We also noted some instances of cell's spiking spontaneously (i.e., spiking outside of the window of injected current), however, we chose not to reject these sweeps or cells according to our quality control criteria.

2) Another parameter that would be useful to report, for instance in Figure 3, is the number or frequency of the action potentials, either as an input-output curve (frequency vs. current amplitude) or e.g. maximum firing frequency or frequency at a given current amplitude (see point 3).

- As requested, in Figure 3, we have now included the slope of the FI curve ("FI fit_slope") and the average firing rate of the IPFX-defined "hero sweep" ("avg_rate") as additional features that capture these specific aspects of neuron physiology.

3) A general remark, related to point 2: it appears that the block pulse amplitude sometimes goes up to e.g. 400 pA while in other recordings it only reaches e.g. 150 or 200 pA. This should be noted in the text and would benefit from an explanation why different maximum amplitudes were used as it could limit comparisons between recordings.

- We agree this information can limit potential comparisons across recordings and have included the following additional paragraph in the methods section to clarify the data collection process.
"The stimulus parameters for each recording were not identical across the recording conditions due to technical considerations by the experimentalist. The maximum stimulus may have been changed by the experimenter depending on the cell's input resistance to prevent overload and losing the cell recording. For example, recordings originating from mouse neurons typically were not stimulated beyond 300 pA due to increased potential to overload and loss of the ongoing recording."

4) The description of the statistical tests is missing from the Methods section, please add. Readability could be improved by writing e.g. "214±102 MΩ" rather than "M=214 MΩ, SD=102). Also please report not only the significantly different outcomes but also the non-significant ones, either in the main text or in the figure legend. Please report in each case the statistical test that was used, which is currently sometimes done but is often missing. Particularly for Figure 4A, the authors report "a consistent trend" without giving the statistical details.

- In the updated manuscript, we have implemented these changes as requested. Specifically, we have now updated the relevant sections for figures 3,4 and 5 for reporting statistical findings. We have updated Fig. 4 to compare recordings from the pooled layers L23, L3C and L5 and highlight the different layers with color. Additionally,

Powered by Editorial Manager® and ProduXion Manager® from Aries Systems Corporation

we have added specific information in the methods regarding the statistical analyses.

5) Figure 5: Were statistical tests done for the data presented here? If yes, please report the outcomes; if not, please explain why not. For the comparison male vs. female: please clarify whether the authors pooled all recordings from all brain regions. To be honest I'm not really seeing the added benefit of the comparison epilepsy vs. tumor especially given that, as the authors state, "most of these patients with tumors also have epilepsy" which makes this a bit of a messy comparison. In addition the number of datapoints is heavily skewed towards the 'epilepsy' group.

- We agree that the tumor vs epilepsy comparison added little benefit and have now removed this comparison from Figure 5. In the updated manuscript, we have pooled the recordings from all regions but now distinguish cells recorded from different layers using color. We have updated the text and associated figure legend to correspond to the updated figure based on your recommendations.

Minor points:
1) Given that the authors report differences in the electrophysiological properties that depend on the slice solution used (NMDG or sucrose-based), it would be useful to highlight in Figure 1 or at least in the data files (see minor point 2) which samples were prepared using which solution.

- We agree that enabling such a comparison is important for reuse of these datasets. As requested, we have now added a column to the metadata sheet, termed ‘dandi_id’, that will more easily facilitate the matching of each recording uploaded to DANDI with its corresponding metadata as provided in our spreadsheet.

2) The authors made the electrophysiological recordings readily available. For the final submission it would be useful to structure (or name) the data in such a way that it is clear how the data files correspond to each subject. Currently it is unclear for me which recording comes from which subject/brain region/cell type etc.

- As requested, we have now added a column to the metadata sheet, termed ‘dandi_id’, that will facilitate the matching of each recording uploaded to DANDI with its corresponding metadata as provided in our spreadsheet.

3) Page 13 "we demonstrate that the inclusion … suprathreshold stimuli"
Is this in line with other research? Also, I suggest to rephrase 'noticeable' to e.g. 'significant' or 'detectable'

- We have added a sentence in the discussion with references to show how our findings are in line with other research:
However, we did not observe a concurrent change in excitability characteristics such as the AP width, in agreement with previous findings that did not find a significant effect of synaptic blockers on AP characteristics or neuronal passive properties [31], [32].

We thank the reviewer for their suggestions to improve the language and have changed any use of “noticeable” to be more clear.

4) Page 13 "Due to the confounding [factor?] … human cortex"
I think what the authors mean is that they want to reduce the variability due to different cortical layers? But I'm not sure, so I think this sentence would benefit from rewriting.

- We agree this section was confusing and have revisited Figure 4. We now pool the recordings across the L23/L3C/L5 layers for statistical comparison and color them to provide the readers more context about the different layer of origin of the neuronal recording.

5) Figure 4 could benefit from some raw traces to exemplify the differences

- We agree and have added example traces to figure 4 which highlight the selected recordings and the effect on input resistance in response to a hyperpolarizing pulse.

6) The data in Figure 6 could benefit from e.g. an R2 value to quantify the

correlation

- We thank the reviewer for the suggestion and have included the values directly in an updated version of the plot.

Reviewer #2:
1) The properties of human cortical neurons are of high interest given their involvement in the particular cognitive abilities of humans; however, they cannot be obtained and studied as readily as in other organisms for obvious reasons. Therefore, publicly sharing the experimental results from human neurosurgical resections provides a considerable benefit to the community. The study of Howard et al. presents recordings from nearly 140 human neocortical neurons recorded from in vitro slices derived from neurosurgical tissues and makes them available via the DANDI archive. The manuscript is straightforward and primarily serves to describe the data set along its various dimensions (subject metadata, recording conditions, etc.), rather than describe novel findings derived from the data set. As such, it seems appropriately submitted here as a Data Note.

The data appear to be of high quality and are also of high utility, as mentioned above. However, the manuscript and data set would benefit from a few areas of improvement.

The box-and-whisker plots are informative, but in most cases they are comparing somewhat heterogenous groups of cells (i.e., cells from different layers, pyramidal cells and interneurons together). It would be helpful to indicate these factors by coloring the individual data points, so that a reader might see if, for example, if there are any trends in the data that follow cortical layer.

- We thank the reviewer for this suggestion and have updated Figures 4 and 5 to more clearly color cells by relevant factors (layers, cell type, etc) to more clearly convey the differences across groups.

2) My main issues actually involved accessing the data rather than the manuscript itself. I am basing my comments on the metadata and feature files linked in the "Data Availability" section.

First, the metadata file contains more rows than there are cells described in the data set. For example, for subject 1914, there are seven rows with that identifier, but only two files in the DANDA archive with that identifier. Among those 7 entries, there are duplicate "Cell #" entries (C1 and C2 appear twice), and five of the entries have a "ZD status" of ZD (the other two have "n.a.") and those five also have empty values for "cutting_solution". Should those rows have been excluded from the spreadsheet? Do the multiple rows with C1 and C2 refer to the same cells? If so, what happened to cells C3, C4, and C5 in the DANDI data set? Also, the use of ZD is not discussed in the manuscript, so that field's presence in the file is a bit confusing as well.

In general, I'm not sure why there are a number of empty values in the metadata spreadsheet. "cutting_solution" is missing for a number of cells (including those mentioned above), and the mouse cells don't have an entry for layer. There are other inconsistencies in how the metadata are encoded; for example, the manuscript says there are four interneurons among the human cells (Table 1). If you filter the metadata file for the "putative_interneuron" field to be TRUE, only three human cells are present. But there is another cell that has an entry of "int" for the "aggregated_cell_layer" column - is this the fourth interneuron? And is its layer not known?

It also appears that the "Cell #" field is not always filled out correctly - for example, for the subject "X2019.11.28" all the cells have a value of "1" in this column. This makes it difficult to associate metadata with DANDI files, as discussed next.

- We appreciate this feedback and have re-organized the provided metadata sheets to be more clear and useful for potential re-use.

Powered by Editorial Manager® and ProduXion Manager® from Aries Systems Corporation

	We now link to an updated metadata sheet that includes only the recordings and fields that are immediately relevant to the dataset published on DANDI (taking care to remove fields like whether cells received ZD - as this is not pertinent to any of the cells in this dataset). We have also fixed the highlighted inconsistency for the putative interneurons and their known layer of origin.<br><br>3) Overall, it is difficult to find the data associated with a particular cell. In DANDI, the data are organized by subject, but the individual files are named in way that seems to have no relation to other cell-specific identifiers. I resorted to downloading all the cells for a particular subject and then examining the `general/subject/description`field in each of the NWB files to find the corresponding file. It would be very helpful to have the DANDI filenames or URLs in the metadata file as well (or in an additional file). I know that the DANDI links are not permanent until the datasets are published (they appear to still be in Draft mode when I examined them), so perhaps the authors are waiting for that before putting that kind of file together. But the addition of that file is critical to easy access of the data.<br><br>- As requested, we have now added a column to the metadata sheet, termed 'dandi_id', that will more easily facilitate the matching of each recording uploaded to DANDI with its corresponding metadata as provided in our spreadsheet.<br><br>4) Finally, the authors mention that there are seven morphologies associated with the cells in the data set in the NeuroMorpho database, but again there are no direct links to these cells in the manuscript or metadata files.<br><br>- We have added a column for the neuromorpho_ID to link the recordings with the uploaded morphologies on Neuromorpho.org.<br><br>5) In a more general comment, I note the stimulus protocols presented to the cells in this data set are limited only to step current injections of ~0.5 s to 1 s. While this cannot be changed now, if the authors intend to add more cells to the data set, they might consider expanding the stimulus set to characterize the properties of their cells in other ways, which could enable more complex modeling efforts or characterizations of human neuron types.<br><br>- We appreciate this comment and will take care to more systematically probe cells for stimuli of varied types in future studies.<br><br>Minor point:<br>In Application Scenarios, paragraph 2: The sentence "In particular, we highlight that in some instances, it may be more suitable to constrain biophysical models to human data in the presence of synaptic blockers, that is to say, when background synaptic activity is having a significant effect on input resistance measurements." This seems self-contradictory; did the authors perhaps mean "absence" instead of "presence"?<br><br>- Yes, we have corrected this sentence. Thank you for pointing out this error.
**Additional Information:**	
**Question**	**Response**
Are you submitting this manuscript to a special series or article collection?	No
**Experimental design and statistics**<br><br>Full details of the experimental design and statistical methods used should be given in the Methods section, as detailed in our Minimum Standards Reporting Checklist. Information essential to interpreting the data presented should be made available	Yes

in the figure legends.<br><br>Have you included all the information requested in your manuscript?	
**Resources**<br><br>A description of all resources used, including antibodies, cell lines, animals and software tools, with enough information to allow them to be uniquely identified, should be included in the Methods section. Authors are strongly encouraged to cite Research Resource Identifiers (RRIDs) for antibodies, model organisms and tools, where possible.<br><br>Have you included the information requested as detailed in our Minimum Standards Reporting Checklist?	Yes
**Availability of data and materials**<br><br>All datasets and code on which the conclusions of the paper rely must be either included in your submission or deposited in publicly available repositories (where available and ethically appropriate), referencing such data using a unique identifier in the references and in the "Availability of Data and Materials" section of your manuscript.<br><br>Have you have met the above requirement as detailed in our Minimum Standards Reporting Checklist?	Yes

Manuscript

Click here to access/download;Manuscript;Revised_manuscript.docx

An in vitro whole-cell electrophysiology dataset of human cortical neurons

Abstract

Background

Whole-cell patch-clamp electrophysiology is an essential technique for understanding how single neurons translate their diverse inputs into a functional output. The relative inaccessibility of live human cortical neurons for experimental manipulation has made it difficult to determine the unique features of how human cortical neurons differ from their counterparts in other species.

Findings

We present a curated repository of whole-cell patch-clamp recordings from surgically resected human cortical tissue, encompassing 118 neurons from 35 individuals (age ranging 21-59 years old; 17 male, 18 female). Recorded human cortical neurons derive from layers 2&3 (L2&3), deep layer 3 (L3c) or layer 5 (L5) and are annotated with a rich set of subject and experimental metadata. For comparison, we also provide a limited set of comparable recordings from 21-day old mice (11 cells from 5 mice). All electrophysiological recordings are provided in the Neurodata Without Borders (NWB) format and are available for further analysis via the Distributed Archives for Neurophysiology Data Integration (DANDI) online repository. The

associated data conversion code is made publicly available and can help others in converting electrophysiology datasets to the open NWB standard for general re-use.

Conclusion

These data can be used for novel analyses of biophysical characteristics of human cortical neurons including in cross-species or cross-lab comparisons or in building computational models of individual human neurons.

Authors

Derek Howard[1]*, Homeira Moradi Chameh[2]*, Alexandre Guet-McCreight[1], Huan Allen Hsiao[1], Maggie Vuong[1], Young Seok Seo[2], Prajay Shah[2], Anukrati Nigam[1,5], Yuxiao Chen[1], Melanie Davie[1], Etay Hay[1,3], Taufik A Valiante[2,3,5,6,7,8,9,10]*, Shreejoy J Tripathy[1,3,4,5]*

[1] Krembil Centre for Neuroinformatics, Centre for Addiction and Mental Health

[2] Krembil Brain Institute, University Health Network, Toronto, ON Canada

[3] Department of Physiology, University of Toronto

[4] Department of Psychiatry, University of Toronto.

[5] Institute of Medical Sciences, Temerty Faculty of Medicine, University of Toronto.

[6] Department of Surgery, Division of Neurosurgery, University of Toronto

[7] Institute of Biomedical Engineering, University of Toronto

[8] Department of Electrical and Computer Engineering, University of Toronto

[9] Max Planck - University of Toronto Center for Neural Science and Technology, Toronto, Canada

[10] Center for Advancing Neurotechnological Innovation to Application (CRANIA), Toronto,Canada

*Denotes equal contribution

Dataset Description

Intracellular electrophysiology, as performed via the whole-cell patch-clamp technique, is a hallmark method for characterizing the biophysical features of neurons. While there have been numerous datasets characterizing these features from cortical neurons in the rodent brain [1]–[4], there are comparatively fewer resources that provide high-quality whole-cell patch-clamp recordings from human cortical neurons due to the relative inaccessibility of human tissue.

However, collaborations between neurosurgeons and basic neuroscientists have recently made it possible to characterize living cortical neurons in brain slices immediately prepared from biopsies following routine neurosurgery [5]–[15]. Still, there remain relatively few datasets of human cortical neuron physiology that are openly-accessible and free for reuse to complement and compare to the the Allen Brain Institute Cell Types Database (http://celltypes.brain-map.org/) [7].

Here, we describe an openly-accessible dataset of electrophysiological recordings from human and mouse cortical neurons. The dataset encompasses 132 whole-cell patch-clamp recordings from surgically resected human tissue (118 cells from 35 individuals) or from 21-day old mice (11 cells from 5 mice). These datasets are made available in the Neurodata Without Borders (NWB) electrophysiology data format via the DANDI data archive. We provide morphological reconstructions for N = 7 cells, made available at NeuroMorpho.org. Each recording is made available with rich subject and experimental protocol metadata, enabling subsequent reuse and comparison with analogous datasets from other species and sources.

Figure 1: Hierarchical summary of human cell recordings and relevant experimental and technical factors, displaying counts of each recording and associated metadata

Methods

Human surgical tissue

Resected human cortical tissues were obtained from Toronto Western Hospital (University Health Network, Canada). All procedures on human tissue were performed in accordance with the Declaration of Helsinki and approved by the University Health Network Research Ethics board [16]. Patients underwent a standardized temporal, parietal or frontal lobectomy under general anesthesia using volatile anesthetics for seizure or tumor treatment [17], [18]. Tissue was obtained from patients diagnosed with temporal (n=34), frontal (n=1) or parietal lobe (n=1) epilepsy or brain tumors (n=4) in 17 male and 18 female patients, age ranging 21 to 59 years (mean age ± SD: 40.5 ± 12.0). Written informed consent were obtained from all study participants to use their tissue and to share the acquired data with anonymized demographic information - namely, subject age at time of surgery, sex, years of seizure, seizure frequency, secondarily generalized seizure frequency (using clinical records and epilepsy monitoring unit recordings), antiepileptic drug treatment, and type of seizure.

The resected cortical tissue from the temporal lobe-middle temporal gyrus (MTG) exhibited no structural or functional abnormalities in preoperative MRI, and is considered “relatively healthy” by ourselves and others as it is located outside of the site of epileptogenesis [6], [9], [12], [13]. Cortical tissue from the frontal cortex from patients with epilepsy are considered as “epileptogenic” tissue and are confirmed with independent electrocorticography (ECoG) (and are annotated as such in our metadata). For tumor cases, cortical tissue blocks were obtained

from tissue at a distance from the main site of the tumor (i.e., such cortical tissue was not taken directly from the tumor itself).

Mouse specimens

All experimental procedures involving mice were reviewed and approved by the animal care committees of the University Health Network in accordance with the guidelines of the Canadian Council on Animal Care. Mixed male and female wild type C57Bl/6J, age postnatal 21 days old were used for experiments. Mice were kept on a 12-hour light/dark cycle and had free access to food and water.

Acute brain slice preparation

Immediately following surgical human cortical resection, the cortical specimens were submerged in an ice-cold (~4°C) cutting solution that was continuously bubbled with carbogenated (95% O_2/5% CO_2) aCSF containing (in mM) sucrose 248, KCl 2, $MgSO_4.7H_2O$ 3, $CaCl_2.2H_2O$ 1, $NaHCO_3$ 26, $NaH_2PO_4.H_2O$ 1.25, D-glucose 10. The osmolarity was adjusted to 300-305 mOsm. Transverse brain slices (400 µm) were sectioned using a vibratome (Leica 1200 V) in cutting solution. Tissue slicing was performed perpendicular to the pial surface to help ensure that pyramidal cell dendrites were minimally truncated [6], [11], [17]. The cutting solution was the same as used for transport of tissue from the operating room to the laboratory. The time between tissue resection and slice preparation was less than 10 min. After sectioning, the slices were incubated for 30 min at 34 °C in standard artificial cerebrospinal fluid (aCSF) (in mM): NaCl 123, KCl 4, $CaCl_2.2H_2O$ 1, $MgSO_4$.7H2O 1, $NaHCO_3$ 26, NaH_2PO_4.H2O 1.2, and D-glucose 10, pH 7.40. All aCSF and cutting solutions were continuously bubbled with carbogen gas (95% O_2-5% CO_2) and had an osmolarity of 300-305 mOsm. Following this incubation, the

slices were maintained in standard aCSF at 22–23 °C for at least 1 h, until they were individually transferred to a submerged recording chamber.

Brain slice preparation was done in a similar way for mice and human tissue. Mice were deeply anesthetized by isoflurane 1.5-3.0%. After decapitation, brains submerged in (~4°C) cutting solution that was continuously bubbled with 95% O_2-5% CO_2 containing (in mM) sucrose 248, KCl 2, $MgSO_4.7H_2O$ 3, $CaCl_2.2H_2O$ 1, $NaHCO_3$ 26, $NaH_2PO_4.H_2O$ 1.25, D-glucose 10. Mouse somatosensory cortical slices (350 μm) were prepared in the coronal plane similar to human slice preparation as described above.

A subset of cortical slices in both human and mouse were prepared using N-Methyl-D-glucamine (NMDG) protective recovery method [19]. The cortical tissue blocks were transferred and sectioned in 2–4 °C NMDG-HEPES aCSF solution containing (in mM): NMDG 92, KCl 2.5, NaH_2PO_4 1.25, $NaHCO_3$ 30, HEPES 20, Glucose 25, Thiourea 2, Na L-ascorbate 5, Na-Pyruvate 3, $CaCl_2.4H_2O$ 0.5, and $MgSO_4.7H_2O$. The pH of NMDG-HEPES aCSF solution was adjusted to 7.3–7.4 using hydrochloric acid and the osmolarity was 300–305 mOsm. The cortical slices were prepared using vibratome as described above. After slicing, slices were transferred to a recovery chamber filled with 32–34 °C NMDG-HEPES aCSF solution which continuously bubbled with 95% O_2–5% CO_2. After 12 min, the slices were transferred to an incubation solution-HEPES aCSF-containing (in mM): NaCl 92, KCl 2.5, $NaH_2PO4.H_2O$ 1.25, $NaHCO_3$ 30, HEPES 20, Glucose 25, Thiourea 2, Na L-ascorbate 5, Na-Pyruvate 3, $CaCl_2.4H_2O$ 2, and $MgSO_4.7H_2O$ 2. After 1-h incubation at room temperature, slices were transferred to a recording chamber and continuously perfused with aCSF containing (in mM): NaCl 126, KCl 2.5, $NaH_2PO_4.H_2O$ 1.25, $NaHCO_3$ 26, Glucose 12.6, $CaCl_2.2H_2O$ 2, and $MgSO_4.7H_20$ [6].

Whole-cell patch-clamp recording from human and mice cortical slices

For electrophysiological recordings, cortical slices were placed in a recording chamber mounted on a fixed-stage upright microscope (Axioskop 2 FS MOT; Carl Zeiss, Germany). Slices were continuously perfused with carbogenated (95% O_2/5% CO_2) aCSF containing of (in mM): NaCl 123, KCl 4, $CaCl_2.2H_2O$ 1.5, $MgSO_4.7H_2O$ 1.3, $NaHCO_3$ 26, $NaH_2PO4.H_2O$ 1.2, and D-glucose 10, pH 7.40 at 32-34 °C. Cortical neurons were visualized using an IR-CCD camera (IR-1000, MTI, USA) with a 40x water immersion objective. Patch pipettes (3-6 MΩ) were pulled from standard borosilicate glass pipettes (thin-wall borosilicate tubes with filaments, World Precision Instruments, Sarasota, FL, USA) using a vertical puller (PC-10, Narishige). For somatic recording of electrophysiological properties, patch pipettes were filled with intracellular solution containing (in mM): K-gluconate 135; NaCl 10; HEPES 10; $MgCl_2$ 1; Na_2ATP 2; GTP 0.3, pH adjusted with KOH to 7.4 (290–309 mOsm). A subset of data were collected with excitatory (APV 50 μM, Sigma; CNQX 25 μM, Sigma) and inhibitory (Bicuculline 10 μM, Sigma; CGP-35348 10 μM, Sigma) synaptic activity blocked.

Electrical signals were measured with Multiclamp 700A amplifier, Axopatch 200B amplifier, pClamp 9.2 and pClamp 10.6 data acquisition software (Axon instruments, Molecular Devices, USA). Subsequently, electrical signals were digitized at 20 kHz using a 1320X digitizer or a 1440A digitizer (Axon instruments, Molecular Devices, USA). The access resistance was monitored throughout the recording (typically between 8-20 MΩ), and neurons were discarded if the access resistance was > 25 MΩ. Recordings were not corrected for bridge balancing due to the short duration of recording time.We note that stimulus parameters for each recording are not identical across recorded cells, in part due to technical considerations by the experimentalist, for example, to prevent losing the cell recording.

Axon Binary Format to Neurodata Without Borders file conversion

The x-to-nwb repository was used to convert current clamp recordings in axon binary format (ABF) to Neurodata Without Borders (NWB) format. Separate converters were used for files recorded using pClamp 9.0, which output ABFv1 files, and pClamp >10.0, which output ABFv2 files, to ensure valid conversions while incorporating the essential metadata. The key aspects of our usage of these data conversion computer scripts relate to defining which ABF channels correspond to stimulus and response traces and ensuring that appropriate scale and offset factors are applied properly upon conversion. We incorporated the ndx-dandi-icephys metadata extensions to allow for inclusion of user-defined 'Subject' and 'Lab' metadata fields to be able to include specific metadata including 'subject_id', 'age', 'species', 'cell_id' and 'tissue_sample_id'.

Relevant metadata was recorded in two separate tables: first, patient level information, including demographics and clinical information; and second, recording specific information, which relate to aspects of each individual cell's recording, such as channels corresponding to stimulus, response, and resting membrane potential. The patient level demographics table included fields including 'Resection date', 'Resection procedure', 'Sex', 'Age', 'Years of seizure history', 'Diagnosis', ' Seizure type', 'Presence of a tumor', 'Antiepileptic drugs'. Recording specific metadata includes experiment 'date', 'Cell number' to differentiate recordings from distinct cells taken on the same day, 'Cell layer', 'Gain', 'Offset', 'Response channel', 'Command channel' and 'RMP' to record the resting membrane potential at the initial time of recording. Additional recording metadata was extracted directly from ABF files using custom scripts to extract the stimulus start and end times and the stimulus sampling rate.

Electrophysiology feature extraction

The Intrinsic Physiology Feature Extractor (IPFX) toolbox was used to extract features from converted NWB files [3], [20]. All experiments consisted of long-square hyperpolarizing and depolarizing current injections, and extracted features include subthreshold features (i.e., input resistance, sag ratio), action potential properties (i.e., action potential half-width, threshold time and voltage) derived from the rheobase spike as well as multi-action potential spike train features derived from the IPFX-defined “hero” sweep (i.e., adaptation index), as described previously [6]. Our included metadata files contain stimulus start and end times along with an IPFX-compatible stimulus description ontology file for reproducibility and to facilitate the feature extraction process.

Quality control of contributed neuron recordings

We performed both automated and manual quality control checks of converted recordings to ensure dataset quality and maximize reuse potential. Using features automatically extracted via IPFX, we checked whether the baseline voltage of a sweep (i.e., v_baseline) deviated by more than 10mV from the initial measure in the first current injection step. Any cell recordings that had any sweep deviate beyond the 10mV threshold were not included in the final contributed dataset. We also included the measures for maximum drift of baseline Vm in each recording’s metadata under the field max_drift_Vm.

Also, individual recordings were manually inspected at 3 injected current steps (the most hyperpolarizing pulse, the rheobase and the most depolarizing step). In addition, we further manually inspected each neuron recording’s frequency/input curve to identify any abnormal responses, and also, to identify putative recordings from interneurons.

Following this manual inspection process, we note that in some instances, we observed some evidence for spike saturation at higher steps of current injection. We also noted some instances

of cell’s spiking spontaneously (i.e., spiking outside of the window of injected current), however, we chose not to reject these sweeps or cells according to our quality control criteria.

Statistical analyses

To detect statistical differences across experimental groupings, we report results using the t.test, wilcox.test, ANOVA or Pearson correlation using the statistical functions in base R. All statistical tests were performed using R version 4.1.2. [21]

Results

In Table 1, we summarize the three main axes differentiating the cells and recordings in this dataset. Namely, recordings differed by species (human versus mouse), by cortical layer of the cell body of the recorded neuron (Layer 23, Layer 3c, and Layer 5), and whether synaptic blockers were used in the external recording solution. Putative interneurons were identified by their action potential characteristics (large maximal firing rates and typically large spike after-hyperpolarization amplitudes) as described in Chameh, et al 2021. One reason why synaptic blockers were used is to make a subset of recordings more consistent with protocols used in other labs, such as the Allen Institute for Brain Sciences [3], [11].

Dataset Overall Type	# cells	# individuals	Number of Pyr cells	Number of ints	L23	L3c	L5
Human (aCSF)	81	19	80	1	27	14	40 (1 int)
Human (aCSF containing synaptic blockers)	37	16	34	3	14 (2 int)	5 (1 int)	18

Mouse (aCSF containing synaptic blockers)	11	5	10	1	0	0	11 (1 int)

Table 1: Summary of number of electrophysiological recordings across major experimental conditions. Putative interneurons (ints) were identified by manual inspection of their electrophysiological characteristics.

In current clamp mode, hyperpolarizing and depolarizing current injections (600-1000 ms) were used to characterize biophysical features of cortical neurons, with examples from three recorded cells shown in Fig. 2.

Figure 2: Example voltage traces from 3 separate L5 neurons, a human L5 neuron recorded in standard aCSF (top), a human L5 neuron recorded in aCSF with synaptic blockers (2nd row), and from a mouse L5 neuron recorded in aCSF with synaptic blockers (third row). Bottom row shows the hyperpolarizing and depolarizing injected step currents that were applied to each neuron and includes the most hyperpolarizing current injection (blue), the rheobase (orange) and most depolarizing current injection (green).

In Fig. 3A, we highlight how the use of synaptic blockers in the external solution may affect recorded subthreshold neuronal properties. Specifically, among recorded human L5 neurons, applying synaptic blockers was associated with a significant increase in the recorded input resistance (standard aCSF: 80.9±36.6 MΩ, n = 40; aCSF containing synaptic blockers: 208±106 MΩ, n = 19; t.test p < 9.37e-05). In Fig. 3C and Fig. 3E we demonstrate that the inclusion of synaptic blockers does not have a detectable effect on either the width of the action potentials elicited in response to suprathreshold stimuli or the average firing rate of the cell at the IPFX-defined “hero” sweep. To illustrate comparisons across species, in Fig. 3B, 3D

and 3F, we show distributions of the input resistance, AP width and average firing rate of the "hero" sweep respectively recorded from neurons in both human and mouse cortical L5 neurons (in the presence of synaptic blockers). We performed statistical comparisons for each of these groups yet note that no significant differences were observed at the $p < 0.05$ threshold.

Figure 3: Distributions of input resistance, AP width and average hero-sweep firing frequency measured in L5 neurons across experimental conditions. Inclusion of synaptic blockers in aCSF has an observable effect on input resistance (A) but not AP width (C) or Avg Rate (E) as measured in human L5 neurons. Input resistance (B), AP width (D), and Avg Rate (F) are not significantly different across mouse and human in L5 neurons recorded in the presence of synaptic blockers in the external solution.

To compare the effect of solution used for the brain slice preparation on intrinsic properties, we compared the input resistance and sag ratio recorded following preparation in either solution. In Fig. 4A, we highlight a significant difference of higher measured input resistance in the recordings made following preparation in the NMDG recovery solution compared to the sucrose solution (NMDG: 266±108 MΩ, n=12; sucrose: 179±75.4 MΩ, n=25; t.test, $p < 0.05$). In Fig. 4B, we compare the sag ratio across the same conditions and observe no significant differences across the brain slice preparations. (NMDG: 0.0960±0.0706, n=12; sucrose: 0.0892±0.0333, n=25, p=0.756). The statistical comparisons made in Fig. 4 were made after grouping all recordings from L23, L3C and L5 using standard aCSF. These comparisons emphasize the potential importance of the conditions used for the experimental preparation (see Discussion).

Figure 4: Distributions of input resistance (A) and sag ratio (B) measured in pooled human L2/3, L3C and L5 neurons derived from different slices, prepared using the NMDG protective solution and a standard sucrose solution preparations. All included recordings were performed using synaptic blockers in aCSF recording solution, with the molecular layer of each recording identified in red (L23), green (L3C) and blue (L5). The 2 recordings from L23 highlighted with a large point indicate the recordings used for the example traces of hyperpolarized steps in (C).

To illustrate the rich diversity of the metadata for each of the human recordings, in Fig. 5 we highlight specific comparisons of input resistance and sag ratio measurements recorded in regular aCSF across demographic conditions. Specifically, we focus on the input resistance as a fundamental passive electrophysiological property and the sag ratio as an active property that has previously been used to distinguish between subtypes of human neurons [6], [11]. In Fig. 5A, 5C we compare distributions of these electrophysiological features across the three different brain lobes from which neuronal tissue was resected. In Fig. 5B, 5D we compare the electrophysiological feature distributions measured in male and female patients (Fig. 5B, D). Note that all cells from frontal and parietal cortex were recorded from tissue resected near the site of the epileptogenic focus, whereas all cells from temporal cortex were recorded distal from the epileptogenic focus (with the exception of 1 subject).

Figure 5: Distributions of input resistance (A, B) and sag ratio (C, D) recorded from pooled human L23, L3C and L5 neurons with standard aCSF. Measurements are grouped and compared by major brain lobe of resection location (A, C) and by sex (B, D).

Additionally, we illustrate the relationship of the input resistance and sag ratio against both patient age at time of surgical resection (Fig. 6A, B) and years of seizure experienced by the patient prior to the surgical intervention (Fig. 6C, D).

Figure 6: Scatterplots of input resistance and sag ratio vs patient age (A, B) and duration of seizure prior to surgical intervention (C, D) recorded in human L5 neurons with standard aCSF. Pearson's correlation values are reported within each plot.

Code Availability

Conversion and analysis scripts are available at:

https://github.com/derekhoward/nwb_conversion

Data Availability

Data is available on the DANDI platform at the following links:

Mouse data: https://dandiarchive.org/dandiset/000292/0.220708.1652

Human data: https://dandiarchive.org/dandiset/000293/0.220708.1652

Other associated metadata for recordings can be found at the following link:

https://github.com/derekhoward/nwb_conversion/blob/master/data/processed/meta/metadata.csv

Application Scenarios

The recordings in this database permit the quantification of biophysical properties from a diverse set of neurons, including human and mouse neurons with a well described set of metadata. Independent variables collected include age, sex, seizure history and cortical layer from which

the tissue was resected. Additionally, experiments on the human neurons were performed with the use of synaptic blockers and without, allowing for comparisons and integration with other intrinsic electrophysiological databases comprising patch-clamp recordings, including from the the Allen Cell types database (https://celltypes.brain-map.org/).

These data from current-clamp experiments are particularly beneficial for the development of conductance-based models of human neurons [11], [22], [23]. In particular, we highlight that in some instances, it may be more suitable to constrain biophysical models to human data in the absence of synaptic blockers, that is to say, when background synaptic activity is having a significant effect on input resistance measurements. The voltage responses can be used as a training set to constrain biophysical models when combined and integrated with other publicly available databases that provide relevant morphologies and channel kinetics, such as NeuroMorpho.org, Channelpedia or ICGenealogy [24]–[26]. Moreover, fitting biophysical models to data that is grouped based on demographic information can allow for cross-group comparisons using in silico approaches. Usage of these models in neuronal or circuit simulations can thus help to further predict and unveil the potential effects of differences in neuronal physiology across demographic groupings [27], [28].

Discussion and Limitations

The repository provided is focused mainly on cortical neurons derived from human tissue. There are comparatively fewer recordings for analyses of mouse neuron function provided and all of these were performed using synaptic blockers that were shown to have a baseline effect on input resistance.

The recordings from human specimens derived from tissue during the surgical resection of diseased tissue for patients with intractable epilepsy or brain tumors. Along with having suffered seizures for an extended period of time, the patients may have concurrently taken one or a variety of anti-epileptic drugs that could have affected baseline neuronal excitability characteristics.

While these data were collected for the purpose of characterizing intrinsic properties of human neocortical neurons, we note that they were collected using different sets of experimental conditions, including those related to different recording solutions as well as cutting solutions. Our analyses suggest such experimental condition differences likely contribute to differences in downstream electrophysiological properties, and are consistent with prior analyses by ourselves and others [2], [29]. For example, the observed effect of synaptic blockers on the input resistance may be due to reduction of overall membrane permeability as a consequence of the block of both excitatory and inhibitory conductances [30]. However, we did not observe a concurrent change in excitability characteristics such as the AP width, in agreement with previous findings that did not find a significant effect of synaptic blockers on AP characteristics or neuronal passive properties [31], [32].

In contrast, the comparisons of electrophysiological measures following different cutting solutions highlight potential effects on neuronal excitability. We observe variability in the measured input resistance but consider that these effects may be due to changes in conductivity across the membrane or also experimental biases in selection of healthy neurons for patch-clamp protocol due to differential response to solutions of different osmolarity. Furthermore, there exists contrasting results in the literature on the effects of NMDG on neuronal excitability and synaptic transmission which shows the context-dependence of the many experimental variables [33], [34]. Taken together, such potential differences in

electrophysiological characteristics due to experimental conditions are important to consider when re-using these data in downstream analyses.

Acknowledgements

We are grateful to our neurosurgical patients and their families for consenting to the use of their tissue samples for research. We thank Dr. Gelareh Zadeh and Dr. Mark Bernstein for their assistance in obtaining brain tissue samples and Victoria Barkley and Marjan Rafiee for assistance in compiling demographic and chart information. We thank Sara Mahallati and Iliya Weisspapir for assistance in tissue preparation. We thank Wataru Inoue and Michael Feyerabend for their critical comments on the manuscript. We acknowledge generous support from the Centre for Addiction and Mental Health Discovery Fund, Krembil Brain Institute Fund, National Institute of Health, and Kavli Foundations.

References

[1] S. J. Tripathy, J. Savitskaya, S. D. Burton, N. N. Urban, and R. C. Gerkin, “NeuroElectro: a window to the world’s neuron electrophysiology data,” *Front. Neuroinform.*, vol. 8, p. 40, Apr. 2014, doi: 10.3389/fninf.2014.00040.

[2] S. J. Tripathy, S. D. Burton, M. Geramita, R. C. Gerkin, and N. N. Urban, “Brain-wide analysis of electrophysiological diversity yields novel categorization of mammalian neuron types,” *J. Neurophysiol.*, vol. 113, no. 10, pp. 3474–3489, Jun. 2015, doi: 10.1152/jn.00237.2015.

[3] N. W. Gouwens *et al.*, “Classification of electrophysiological and morphological neuron types in the mouse visual cortex,” *Nat. Neurosci.*, vol. 22, no. 7, pp. 1182–1195, Jul. 2019, doi: 10.1038/s41593-019-0417-0.

[4] H. Markram *et al.*, “Reconstruction and Simulation of Neocortical Microcircuitry,” *Cell*, vol. 163, no. 2, pp. 456–492, Oct. 2015, doi: 10.1016/j.cell.2015.09.029.

[5] H. Mohan *et al.*, “Dendritic and Axonal Architecture of Individual Pyramidal Neurons across Layers of Adult Human Neocortex,” *Cereb. Cortex*, vol. 25, no. 12, pp. 4839–4853, Dec. 2015, doi: 10.1093/cercor/bhv188.

[6] H. M. Chameh *et al.*, “Diversity amongst human cortical pyramidal neurons revealed via

their sag currents and frequency preferences,” *Nat. Commun.*, vol. 12, no. 1, pp. 1–15, 2021, [Online]. Available: https://www.nature.com/articles/s41467-021-22741-9
[7] J. Berg *et al.*, “Human neocortical expansion involves glutamatergic neuron diversification,” *Nature*, vol. 598, no. 7879, pp. 151–158, Oct. 2021, doi: 10.1038/s41586-021-03813-8.
[8] A. Gidon *et al.*, “Dendritic action potentials and computation in human layer 2/3 cortical neurons,” *Science*, vol. 367, no. 6473, pp. 83–87, Jan. 2020, doi: 10.1126/science.aax6239.
[9] L. Beaulieu-Laroche *et al.*, “Enhanced Dendritic Compartmentalization in Human Cortical Neurons,” *Cell*, vol. 175, no. 3, pp. 643–651.e14, Oct. 2018, doi: 10.1016/j.cell.2018.08.045.
[10] L. Beaulieu-Laroche *et al.*, “Allometric rules for mammalian cortical layer 5 neuron biophysics,” *Nature*, vol. 600, no. 7888, pp. 274–278, Dec. 2021, doi: 10.1038/s41586-021-04072-3.
[11] B. E. Kalmbach *et al.*, “h-Channels Contribute to Divergent Intrinsic Membrane Properties of Supragranular Pyramidal Neurons in Human versus Mouse Cerebral Cortex,” *Neuron*, vol. 100, no. 5, pp. 1194–1208.e5, Dec. 2018, doi: 10.1016/j.neuron.2018.10.012.
[12] B. E. Kalmbach *et al.*, “Signature morpho-electric, transcriptomic, and dendritic properties of human layer 5 neocortical pyramidal neurons,” *Neuron*, vol. 109, no. 18. pp. 2914–2927.e5, 2021. doi: 10.1016/j.neuron.2021.08.030.
[13] R. D. Hodge *et al.*, “Conserved cell types with divergent features in human versus mouse cortex,” *Nature*, vol. 573, no. 7772, pp. 61–68, Sep. 2019, doi: 10.1038/s41586-019-1506-7.
[14] Y. Deitcher *et al.*, “Comprehensive Morpho-Electrotonic Analysis Shows 2 Distinct Classes of L2 and L3 Pyramidal Neurons in Human Temporal Cortex,” *Cereb. Cortex*, vol. 27, no. 11, pp. 5398–5414, Nov. 2017, doi: 10.1093/cercor/bhx226.
[15] C. M. Florez *et al.*, “In vitro recordings of human neocortical oscillations,” *Cereb. Cortex*, vol. 25, no. 3, pp. 578–597, Mar. 2015, doi: 10.1093/cercor/bht235.
[16] A. Mansouri, A. Fallah, and T. A. Valiante, “Determining surgical candidacy in temporal lobe epilepsy,” *Epilepsy Res. Treat.*, vol. 2012, p. 706917, Feb. 2012, doi: 10.1155/2012/706917.
[17] G. Kostopoulos, C. Drapeau, M. Avoli, A. Olivier, and J. G. Villemeure, “Endogenous adenosine can reduce epileptiform activity in the human epileptogenic cortex maintained in vitro,” *Neurosci. Lett.*, vol. 106, no. 1–2, pp. 119–124, Nov. 1989, doi: 10.1016/0304-3940(89)90212-7.
[18] R. Köhling and M. Avoli, “Methodological approaches to exploring epileptic disorders in the human brain in vitro,” *J. Neurosci. Methods*, vol. 155, no. 1, pp. 1–19, Jul. 2006, doi: 10.1016/j.jneumeth.2006.04.009.
[19] J. T. Ting *et al.*, “Preparation of Acute Brain Slices Using an Optimized N-Methyl-D-glucamine Protective Recovery Method,” *J. Vis. Exp.*, no. 132, Feb. 2018, doi: 10.3791/53825.
[20] B. R. Lee *et al.*, “Scaled, high fidelity electrophysiological, morphological, and transcriptomic cell characterization,” *Elife*, vol. 10, Aug. 2021, doi: 10.7554/eLife.65482.
[21] R. Computing and Others, “R: A language and environment for statistical computing,” *Vienna: R Core Team*, 2013, [Online]. Available: https://www.yumpu.com/en/document/view/6853895/r-a-language-and-environment-for-statistical-computing
[22] G. Eyal *et al.*, “Unique membrane properties and enhanced signal processing in human neocortical neurons,” *Elife*, vol. 5, Oct. 2016, doi: 10.7554/eLife.16553.
[23] H. K. Yao *et al.*, “Reduced inhibition in depression impairs stimulus processing in human cortical microcircuits,” *Cell Rep.*, vol. 38, no. 2, p. 110232, Jan. 2022, doi: 10.1016/j.celrep.2021.110232.

[24] G. A. Ascoli, D. E. Donohue, and M. Halavi, “NeuroMorpho.Org: a central resource for neuronal morphologies,” *J. Neurosci.*, vol. 27, no. 35, pp. 9247–9251, Aug. 2007, doi: 10.1523/JNEUROSCI.2055-07.2007.

[25] R. Ranjan *et al.*, “Channelpedia: an integrative and interactive database for ion channels,” *Front. Neuroinform.*, vol. 5, p. 36, Dec. 2011, doi: 10.3389/fninf.2011.00036.

[26] W. F. Podlaski, A. Seeholzer, L. N. Groschner, G. Miesenböck, R. Ranjan, and T. P. Vogels, “Mapping the function of neuronal ion channels in model and experiment,” *eLife*, vol. 6. 2017. doi: 10.7554/elife.22152.

[27] N. A. Goriounova *et al.*, “Large and fast human pyramidal neurons associate with intelligence,” *Elife*, vol. 7, Dec. 2018, doi: 10.7554/eLife.41714.

[28] A. Guet-McCreight *et al.*, “Age-dependent increased sag current in human pyramidal neurons dampens baseline cortical activity,” *bioRxiv*, p. 2021.11.03.467014, Jan. 12, 2022. doi: 10.1101/2021.11.03.467014.

[29] D. Tebaykin, S. J. Tripathy, N. Binnion, B. Li, R. C. Gerkin, and P. Pavlidis, “Modeling sources of interlaboratory variability in electrophysiological properties of mammalian neurons,” *J. Neurophysiol.*, vol. 119, no. 4, pp. 1329–1339, Apr. 2018, doi: 10.1152/jn.00604.2017.

[30] P. A. Núñez-Abades, J. M. Pattillo, T. M. Hodgson, and W. E. Cameron, “Role of synaptic inputs in determining input resistance of developing brain stem motoneurons,” *J. Neurophysiol.*, vol. 84, no. 5, pp. 2317–2329, Nov. 2000, doi: 10.1152/jn.2000.84.5.2317.

[31] T. J. Ashwood and H. V. Wheal, “The expression of N-methyl-d-aspartate-receptor-mediated component during epileptiform synaptic activity in the hippocampus,” *British Journal of Pharmacology*, vol. 91, no. 4. pp. 815–822, 1987. doi: 10.1111/j.1476-5381.1987.tb11280.x.

[32] S. Nedergaard, “Regulation of action potential size and excitability in substantia nigra compacta neurons: sensitivity to 4-aminopyridine,” *J. Neurophysiol.*, vol. 82, no. 6, pp. 2903–2913, Dec. 1999, doi: 10.1152/jn.1999.82.6.2903.

[33] J. B. Thuma and S. L. Hooper, “Choline and NMDG directly reduce outward currents: reduced outward current when these substances replace Na+ is alone not evidence of Na+-activated K+ currents,” *J. Neurophysiol.*, vol. 120, no. 6, pp. 3217–3233, Dec. 2018, doi: 10.1152/jn.00871.2017.

[34] E. M. Avegno, J. W. Middleton, and N. W. Gilpin, “Synaptic GABAergic transmission in the central amygdala (CeA) of rats depends on slice preparation and recording conditions,” *Physiol Rep*, vol. 7, no. 19, p. e14245, Oct. 2019, doi: 10.14814/phy2.14245.

Figure 1

Click here to access/download;Figure;Figure1.png

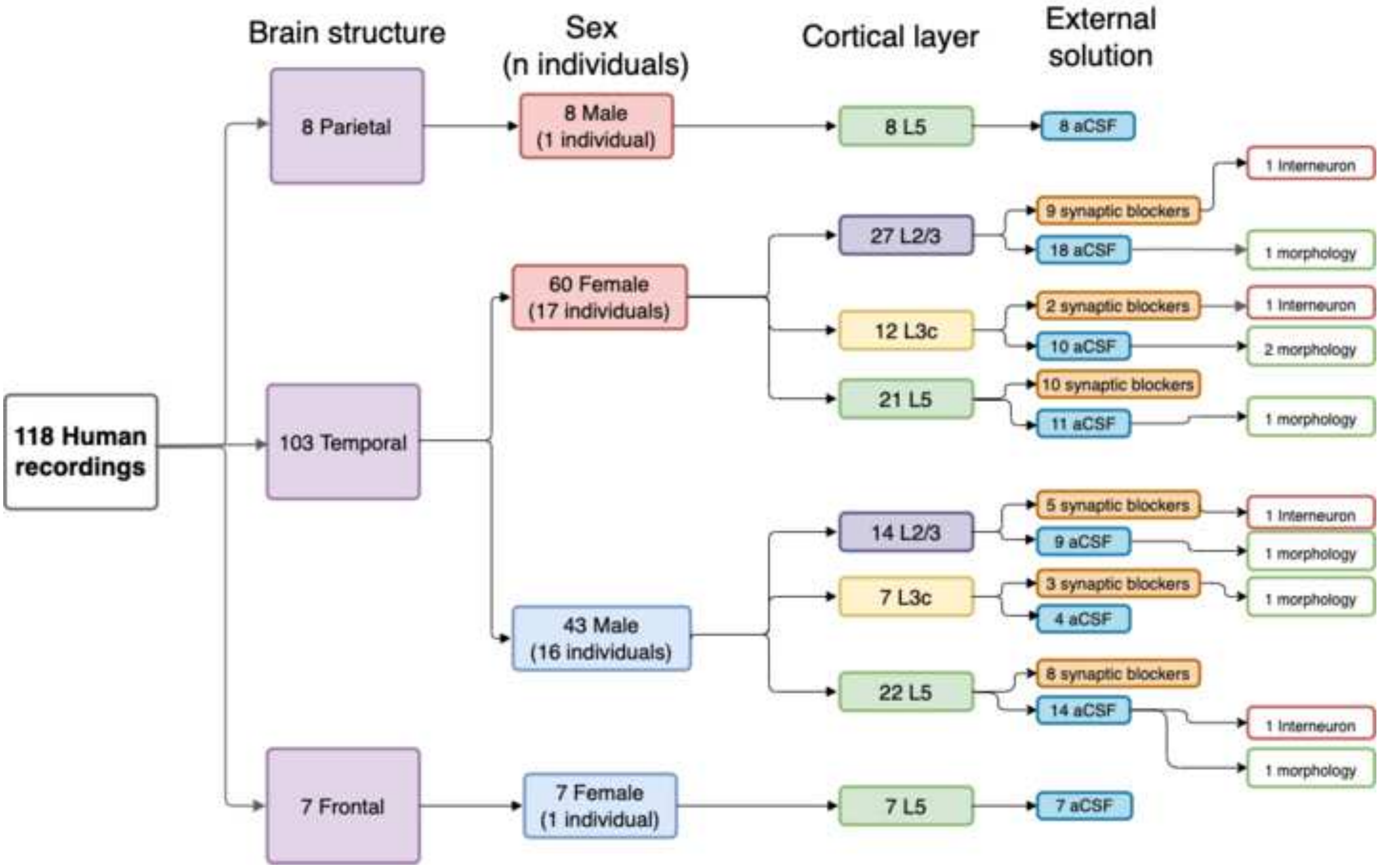

Figure 2

Click here to access/download;Figure;Figure2.png

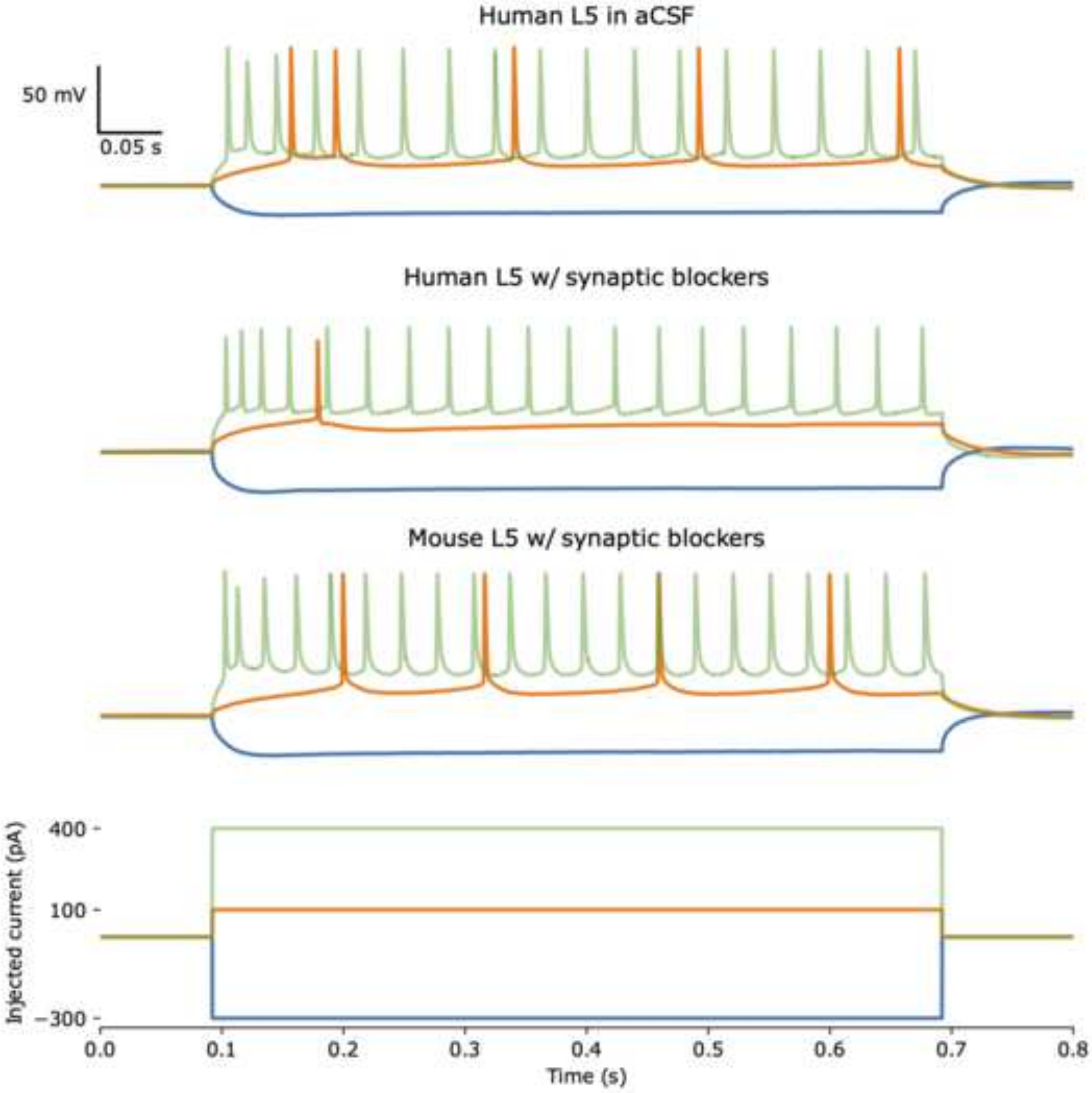

Figure 3

Click here to access/download;Figure;Figure3.png

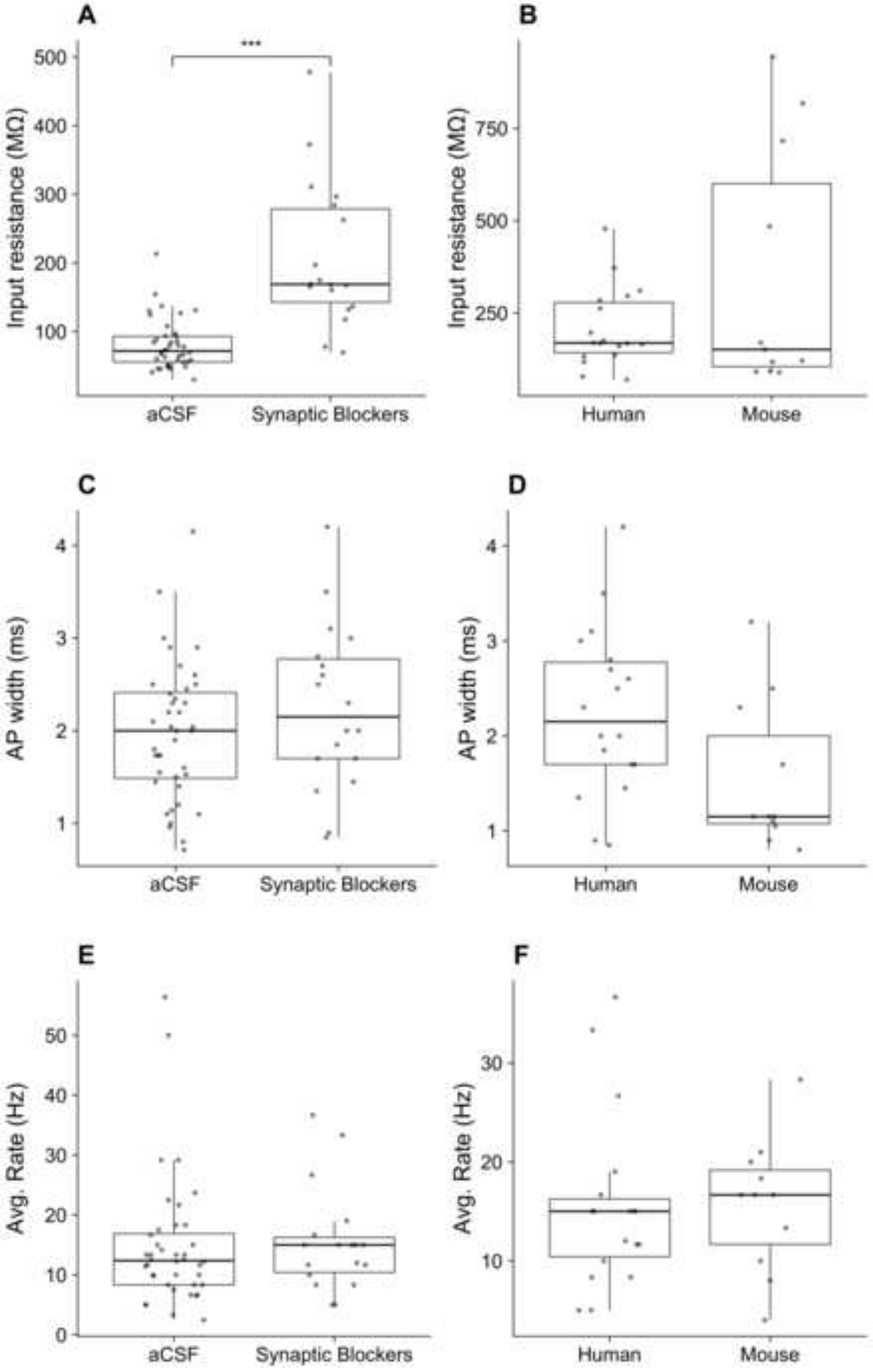

Figure 4

Click here to access/download;Figure;Figure4.png

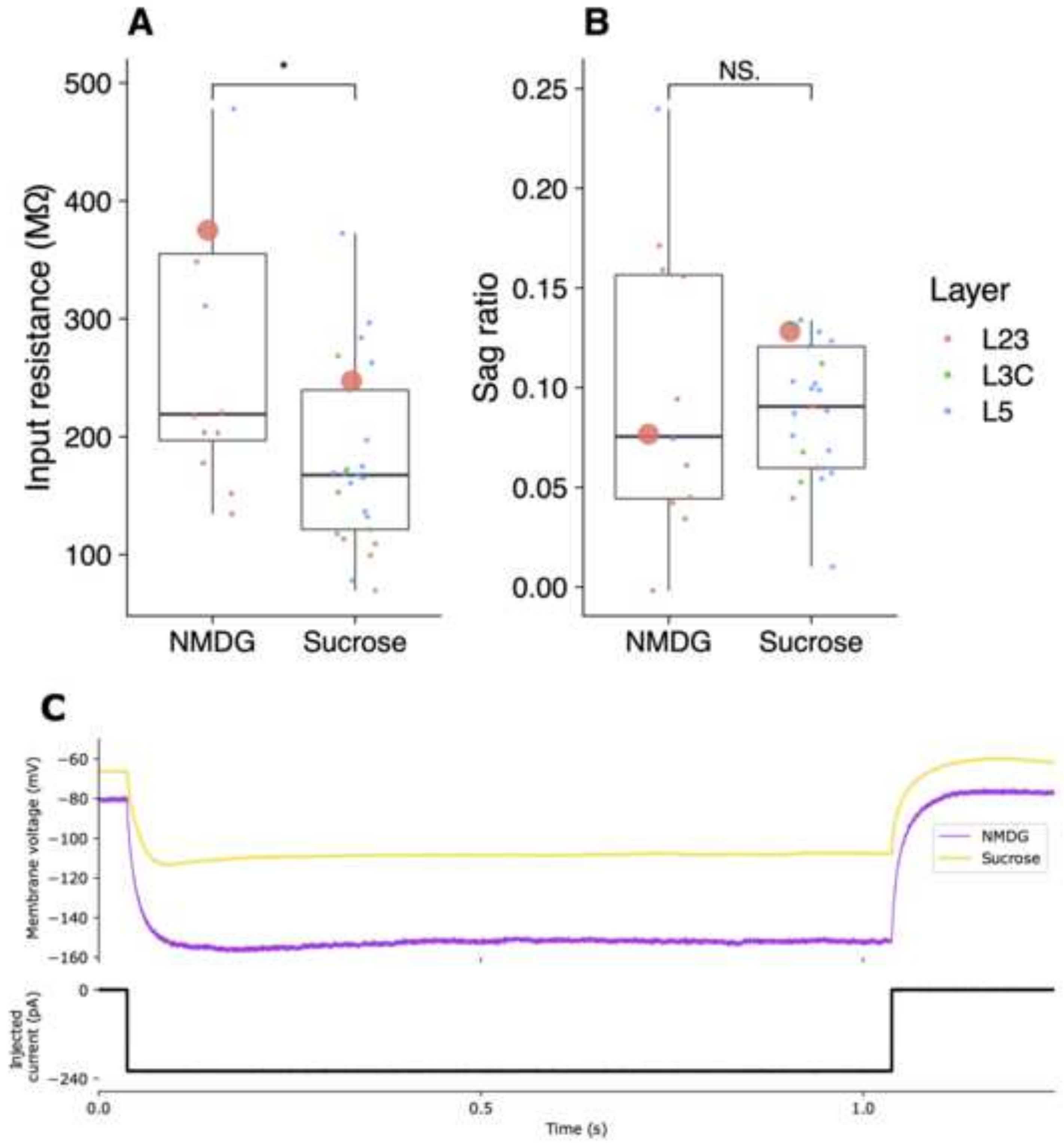

Figure 5

Click here to access/download;Figure;Figure5.png

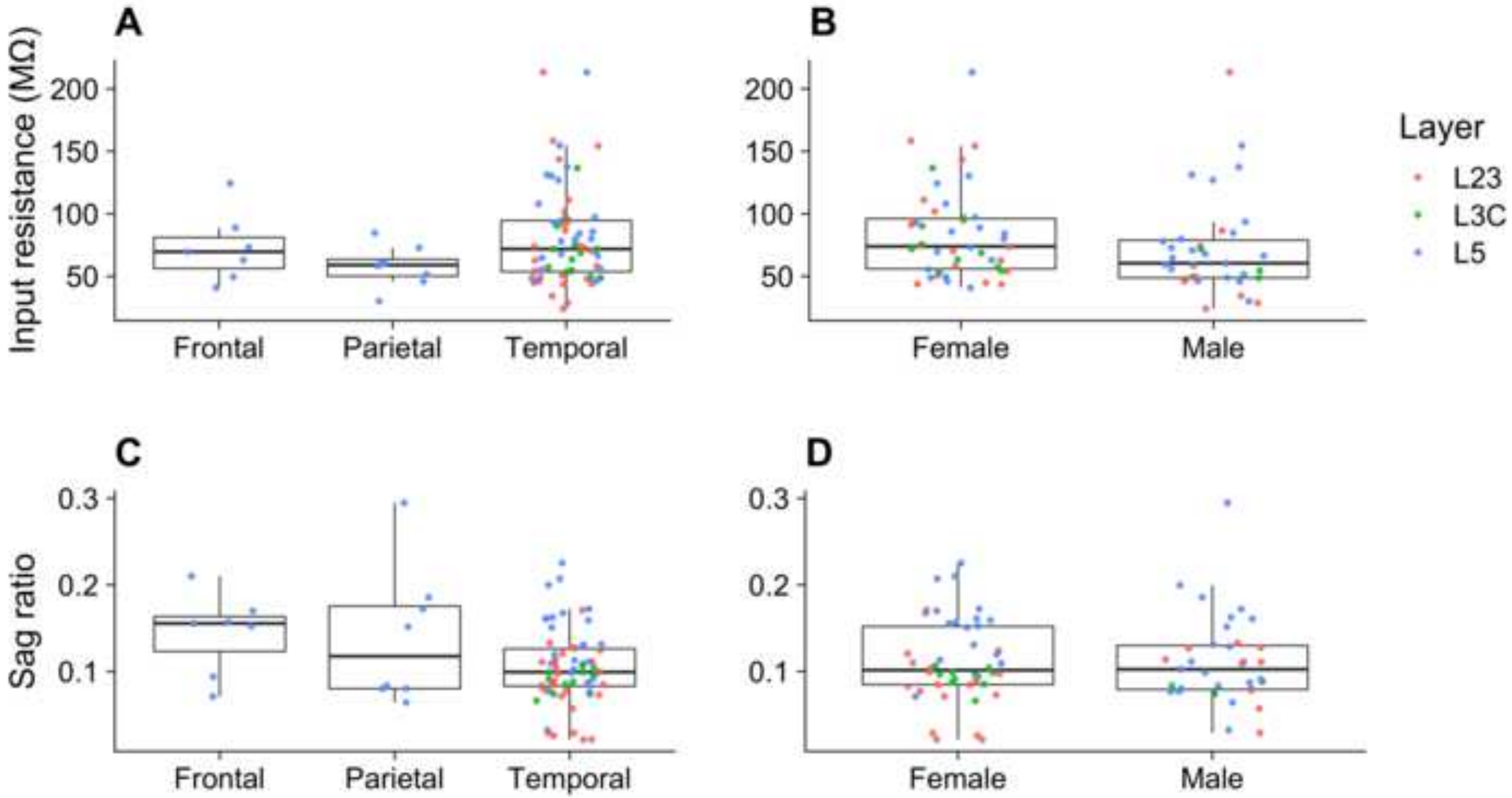

Figure6

Click here to access/download;Figure;Figure6.png

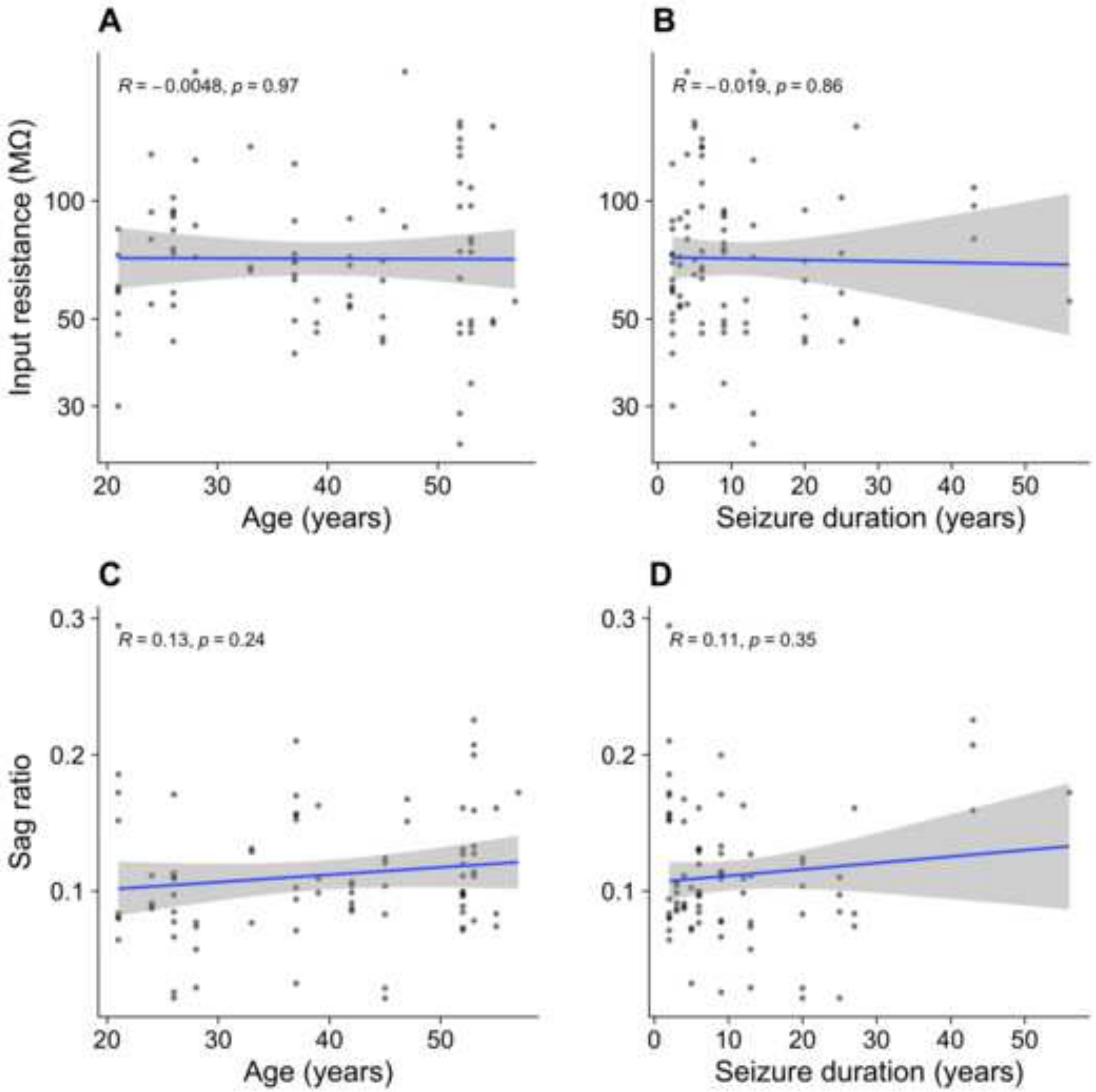

Supplement: giac108_GIGA-D-22-00068_Revision_1 [file giac108_giga-d-22-00068_revision_1.pdf]
