# Supplementary material for: An in vitro whole-cell electrophysiology dataset of human cortical neurons
Source: Gigascience. 2022 Nov 15;11:giac108. doi: 10.1093/gigascience/giac108 (PMC9664072; doi:10.1093/gigascience/giac108)

GigaScience

An in vitro whole-cell electrophysiology dataset of human cortical neurons

--Manuscript Draft--

**Manuscript Number:**	GIGA-D-22-00068R2
**Full Title:**	An in vitro whole-cell electrophysiology dataset of human cortical neurons
**Article Type:**	Data Note
**Funding Information:**	Centre for Addiction and Mental Health (Discovery Fund) — Dr Shreejoy Tripathy<br>Krembil Brain Institute Fund — Dr Taufik A Valiante<br>National Institute of Health — Dr Taufik A Valiante<br>Kavli Foundation — Dr Shreejoy Tripathy

Abstract:

Background

Whole-cell patch-clamp electrophysiology is an essential technique for understanding how single neurons translate their diverse inputs into a functional output. The relative inaccessibility of live human cortical neurons for experimental manipulation has made it difficult to determine the unique features of how human cortical neurons differ from their counterparts in other species.

Findings

We present a curated repository of whole-cell patch-clamp recordings from surgically resected human cortical tissue, encompassing 118 neurons from 35 individuals (age ranging 21-59 years old; 17 male, 18 female). Recorded human cortical neurons derive from layers 2&3 (L2&3), deep layer 3 (L3c) or layer 5 (L5) and are annotated with a rich set of subject and experimental metadata. For comparison, we also provide a limited set of comparable recordings from 21-day old mice (11 cells from 5 mice). All electrophysiological recordings are provided in the Neurodata Without Borders (NWB) format and are available for further analysis via the Distributed Archives for Neurophysiology Data Integration (DANDI) online repository. The associated data conversion code is made publicly available and can help others in converting electrophysiology datasets to the open NWB standard for general re-use.

Conclusion

These data can be used for novel analyses of biophysical characteristics of human cortical neurons including in cross-species or cross-lab comparisons or in building computational models of individual human neurons.

**Corresponding Author:**	Derek Howard<br>CAMH: Centre for Addiction and Mental Health<br>Toronto, Ontario CANADA
**Corresponding Author Secondary Information:**	
**Corresponding Author's Institution:**	CAMH: Centre for Addiction and Mental Health
**Corresponding Author's Secondary Institution:**	
**First Author:**	Derek Howard
**First Author Secondary Information:**	
**Order of Authors:**	Derek Howard
	Homeira Moradi Chameh
	Alexandre Guet-McCreight
	Huan Allen Hsiao

Powered by Editorial Manager® and ProduXion Manager® from Aries Systems Corporation

	Maggie Vuong
	Young Seok Seo
	Prajay Shah
	Anukrati Nigam
	Yuxiao Chen
	Melanie Davie
	Etay Hay
	Taufik A Valiante
	Shreejoy Tripathy
**Order of Authors Secondary Information:**	
**Response to Reviewers:**	We thank the reviewer for their helpful suggestions in improving the clarity of the manuscript. We have addressed the concerns with remarks below:<br><br>1) "As requested, in Figure 3, we have now included the slope of the FI curve ("FI fit_slope") and the average firing rate of the IPFX-defined "hero sweep" ("avg_rate") as additional features that capture these specific aspects of neuron physiology."<br>Thank you for incorporating this parameter. In Figure 3E and 3F I do see "Avg. Rate (Hz)" but note the slope. Perhaps a mislabeled axis?<br><br>- We mistakenly mentioned the FI fit_slope in the last response while including only the 'avg_rate' as a parameter to capture excitability characteristics. 3E and 3F now share a y-axis to better facilitate the comparisons across experimental conditions in human neurons (3E) and across species (3F).<br><br>2) "Additionally, we have added specific information in the methods regarding the statistical analyses."<br>More information on the statistics has indeed been added which has improved the manuscript. However either in the main text or legend I would still suggest that non-significant findings also have a p-value that should be reported which is not always done. Currently the text states e.g. "We performed statistical comparisons for each of these groups yet note that no significant differences were observed at the p < 0.05 threshold."<br><br>Also there are still some inconsistencies in the reporting. For example:<br>"(NMDG: 266±108 MΩ, n=12; sucrose: 179±75.4 MΩ, n=25; t.test, p < 0.05)" Here (and where it is appropriate) I would suggest to report the p-value more precisely (e.g. is it 0.013 or 0.049?)<br>"(NMDG: 0.0960±0.0706, n=12; sucrose: 0.0892±0.0333, n=25, p=0.756)" Here the p-value is reported precisely but the test is not reported. I assume this was also tested using a t-test? Please report throughout the manuscript<br><br>- We have revisited this section to more precisely report the statistics in the text and have also updated later sections (for Fig 4 and 5) to more clearly communicate statistical findings in the text. Pasted section for Fig3 below:<br><br>"In Fig. 3A, we highlight how the use of synaptic blockers in the external solution may affect recorded subthreshold neuronal properties. Specifically, among recorded human L5 neurons, there was a significant difference in the recorded input resistance between neurons recorded following application of synaptic blockers (208±106 MΩ, n=18) and regular aCSF (80.9±36.6 MΩ, n=40); t(19)=4.94, p=9.37e-05. However, in Fig. 3C there was no significant effect on the action potential width, t(28)=1.14, p=0.265, between neurons recorded following application of synaptics blockers and regular aCSF. Similarly, in Fig. 3E there was no detectable effect on the average firing rate of the cell at the IPFX-defined "hero" sweep, t(40)=0.259, p=0.797, between the same groups. To illustrate comparisons across species, in Fig. 3B, 3D and 3F, we show distributions of the input resistance, AP width and average firing rate of the "hero" sweep respectively recorded from neurons in both human and mouse cortical L5 neurons (in the presence of synaptic blockers). We did not detect a significant

Powered by Editorial Manager® and ProduXion Manager® from Aries Systems Corporation

	difference in the input resistance, t(11)=1.32, p=0.210, or average firing rate, t(25)=0.0709, p=0.944, observed between the recordings from the two species. However, when comparing the width of APs from recordings in human neurons (2.25±0.890 ms, n=18) and mouse neurons (1.55±0.783, n=11), there was a significant difference detected, t(23)=2.23, p=0.0355.“<br><br>3) For Figure 5 statistical comparisons are still missing. Please report the outcomes. Or, if it is not useful to make statistical comparisons please explain in the main text.<br><br>- We now mention the statistical comparisons directly in the text. Pasted below:<br>“In Fig. 5A, 5C we compare distributions of these electrophysiological features across the three different brain lobes from which neuronal tissue was resected. Kruskal-Wallis rank sum test was used to examine whether brain lobe resection location on input resistance or measured sag ratio. No significant differences in input resistance (chi-squared = 2.7968, df = 2, p-value = 0.247), or sag ratio (chi-squared = 3.50, df = 2, p-value = 0.174), were found across the 3 resected locations. In Fig. 5B, 5D we compare the electrophysiological feature distributions measured in male and female patients. We did not detect any differences between recordings from male or female patients in input resistance, t(70)=1.38, p=0.172, or sag ratio, t(70)=0.0644, p=0.949.”
**Additional Information:**	
**Question**	**Response**
Are you submitting this manuscript to a special series or article collection?	No
**Experimental design and statistics**<br><br>Full details of the experimental design and statistical methods used should be given in the Methods section, as detailed in our Minimum Standards Reporting Checklist. Information essential to interpreting the data presented should be made available in the figure legends.<br><br>Have you included all the information requested in your manuscript?	Yes
**Resources**<br><br>A description of all resources used, including antibodies, cell lines, animals and software tools, with enough information to allow them to be uniquely identified, should be included in the Methods section. Authors are strongly encouraged to cite Research Resource Identifiers (RRIDs) for antibodies, model organisms and tools, where possible.<br><br>Have you included the information	Yes

requested as detailed in our Minimum Standards Reporting Checklist?	
**Availability of data and materials**<br><br>All datasets and code on which the conclusions of the paper rely must be either included in your submission or deposited in publicly available repositories (where available and ethically appropriate), referencing such data using a unique identifier in the references and in the “Availability of Data and Materials” section of your manuscript.<br><br>Have you have met the above requirement as detailed in our Minimum Standards Reporting Checklist?	Yes

Manuscript
Click here to access/download;Manuscript;Revised_manuscript-v2-Oct 13.docx

An in vitro whole-cell electrophysiology dataset of human cortical neurons

Authors

Derek Howard[1]*, Homeira Moradi Chameh[2]*, Alexandre Guet-McCreight[1], Huan Allen Hsiao[1], Maggie Vuong[1], Young Seok Seo[2], Prajay Shah[2], Anukrati Nigam[1,5], Yuxiao Chen[1], Melanie Davie[1], Etay Hay[1,3], Taufik A Valiante[2,3,5,6,7,8,9,10]*, Shreejoy J Tripathy[1,3,4,5]*

[1] *Krembil Centre for Neuroinformatics, Centre for Addiction and Mental Health*

[2] *Krembil Brain Institute, University Health Network, Toronto, ON Canada*

[3] *Department of Physiology, University of Toronto*

[4] *Department of Psychiatry, University of Toronto.*

[5] *Institute of Medical Sciences, Temerty Faculty of Medicine, University of Toronto.*

[6] *Department of Surgery, Division of Neurosurgery, University of Toronto*

[7] *Institute of Biomedical Engineering, University of Toronto*

[8] *Department of Electrical and Computer Engineering, University of Toronto*

[9] *Max Planck - University of Toronto Center for Neural Science and Technology, Toronto, Canada*

[10] *Center for Advancing Neurotechnological Innovation to Application (CRANIA), Toronto,Canada*

**Denotes equal contribution*

Corresponding Author E-Mail: derekhoward@gmail.com

Derek Howard [0000-0002-6621-9473];
Homeira Moradi Chameh [0000-0002-8009-147X];
Alexandre Guet-McCreight [0000-0002-2377-0337];
Yuxiao Chen [0000-0001-9493-7918];
Melanie Davie [0000-0001-8183-8064];
Taufik A Valiante [0000-0002-3443-3790];
Shreejoy Tripathy [0000-0002-1007-9061]

Abstract

Background

Whole-cell patch-clamp electrophysiology is an essential technique for understanding how single neurons translate their diverse inputs into a functional output. The relative inaccessibility of live human cortical neurons for experimental manipulation has made it difficult to determine the unique features of how human cortical neurons differ from their counterparts in other species.

Findings

We present a curated repository of whole-cell patch-clamp recordings from surgically resected human cortical tissue, encompassing 118 neurons from 35 individuals (age ranging 21-59 years old; 17 male, 18 female). Recorded human cortical neurons derive from layers 2&3 (L2&3), deep layer 3 (L3c) or layer 5 (L5) and are annotated with a rich set of subject and experimental metadata. For comparison, we also provide a limited set of comparable recordings from 21-day old mice (11 cells from 5 mice). All electrophysiological recordings are provided in the Neurodata Without Borders (NWB) format and are available for further analysis via the Distributed Archives for Neurophysiology Data Integration (DANDI) online repository. The associated data conversion code is made publicly available and can help others in converting electrophysiology datasets to the open NWB standard for general re-use.

Conclusion

These data can be used for novel analyses of biophysical characteristics of human cortical neurons including in cross-species or cross-lab comparisons or in building computational models of individual human neurons.

Dataset Description

Intracellular electrophysiology, as performed via the whole-cell patch-clamp technique, is a hallmark method for characterizing the biophysical features of neurons. While there have been numerous datasets characterizing these features from cortical neurons in the rodent brain [1–4], there are comparatively fewer resources that provide high-quality whole-cell patch-clamp recordings from human cortical neurons due to the relative inaccessibility of human tissue.

However, collaborations between neurosurgeons and basic neuroscientists have recently made it possible to characterize living cortical neurons in brain slices immediately prepared from biopsies following routine neurosurgery [5–15]. Still, there remain relatively few datasets of human cortical neuron physiology that are openly-accessible and free for reuse to complement and compare to the Allen Brain Institute Cell Types Database (Allen Cell Types Database, RRID:SCR_014806) [7].

Here, we describe an openly-accessible dataset of electrophysiological recordings from human and mouse cortical neurons. The dataset encompasses 132 whole-cell patch-clamp recordings from surgically resected human tissue (118 cells from 35 individuals) or from 21-day old mice (11 cells from 5 mice). These datasets are made available in the Neurodata Without Borders (NWB) (Neurodata Without Borders, RRID:SCR_015242) electrophysiology data format via the

DANDI data archive. We provide morphological reconstructions for N = 7 cells, made available at NeuroMorpho.Org (NeuroMorpho.Org, RRID:SCR_002145). Each recording is made available with rich subject and experimental protocol metadata, enabling subsequent reuse and comparison with analogous datasets from other species and sources.

Figure 1: Hierarchical summary of human cell recordings and relevant experimental and technical factors, displaying counts of each recording and associated metadata

Methods

Human surgical tissue

Resected human cortical tissues were obtained from Toronto Western Hospital (University Health Network, Canada). All procedures on human tissue were performed in accordance with the Declaration of Helsinki and approved by the University Health Network Research Ethics board [16]. Patients underwent a standardized temporal, parietal or frontal lobectomy under general anesthesia using volatile anesthetics for seizure or tumor treatment [17,18]. Tissue was obtained from patients diagnosed with temporal (n=34), frontal (n=1) or parietal lobe (n=1) epilepsy or brain tumors (n=4) in 17 male and 18 female patients, age ranging 21 to 59 years (mean age ± SD: 40.5 ± 12.0). Written informed consent were obtained from all study participants to use their tissue and to share the acquired data with anonymized demographic information - namely, subject age at time of surgery, sex, years of seizure, seizure frequency, secondarily generalized seizure frequency (using clinical records and epilepsy monitoring unit recordings), antiepileptic drug treatment, and type of seizure.

The resected cortical tissue from the temporal lobe-middle temporal gyrus (MTG) exhibited no structural or functional abnormalities in preoperative MRI, and is considered “relatively healthy” by ourselves and others as it is located outside of the site of epileptogenesis [6,9,12,13]. Cortical tissue from the frontal cortex from patients with epilepsy are considered as “epileptogenic” tissue and are confirmed with independent electrocorticography (ECoG) (and are annotated as such in our metadata). For tumor cases, cortical tissue blocks were obtained from tissue at a distance from the main site of the tumor (i.e., such cortical tissue was not taken directly from the tumor itself).

Mouse specimens

All experimental procedures involving mice were reviewed and approved by the animal care committees of the University Health Network in accordance with the guidelines of the Canadian Council on Animal Care. Mixed male and female wild type C57Bl/6J, age postnatal 21 days old were used for experiments. Mice were kept on a 12-hour light/dark cycle and had free access to food and water.

Acute brain slice preparation

Immediately following surgical human cortical resection, the cortical specimens were submerged in an ice-cold (~4°C) cutting solution that was continuously bubbled with carbogenated (95% O_2/5% CO_2) aCSF containing (in mM) sucrose 248, KCl 2, $MgSO_4.7H_2O$ 3, $CaCl_2.2H_2O$ 1, $NaHCO_3$ 26, $NaH_2PO_4.H_2O$ 1.25, D-glucose 10. The osmolarity was adjusted to 300-305 mOsm. Transverse brain slices (400 μm) were sectioned using a vibratome (Leica 1200 V) in cutting solution. Tissue slicing was performed perpendicular to the pial surface to help ensure that pyramidal cell dendrites were minimally truncated [6,11,17]. The cutting solution was the same as used for transport of tissue from the operating room to the laboratory.

The time between tissue resection and slice preparation was less than 10 min. After sectioning, the slices were incubated for 30 min at 34 °C in standard artificial cerebrospinal fluid (aCSF) (in mM): NaCl 123, KCl 4, $CaCl_2.2H_2O$ 1, $MgSO_4$.7H2O 1, $NaHCO_3$ 26, NaH_2PO_4.H2O 1.2, and D-glucose 10, pH 7.40. All aCSF and cutting solutions were continuously bubbled with carbogen gas (95% O_2-5% CO_2) and had an osmolarity of 300-305 mOsm. Following this incubation, the slices were maintained in standard aCSF at 22–23 °C for at least 1 h, until they were individually transferred to a submerged recording chamber.

Brain slice preparation was done in a similar way for mice and human tissue. Mice were deeply anesthetized by isoflurane 1.5-3.0%. After decapitation, brains submerged in (~4°C) cutting solution that was continuously bubbled with 95% O_2-5% CO_2 containing (in mM) sucrose 248, KCl 2, $MgSO_4.7H_2O$ 3, $CaCl_2.2H_2O$ 1, $NaHCO_3$ 26, $NaH_2PO_4.H_2O$ 1.25, D-glucose 10. Mouse somatosensory cortical slices (350 μm) were prepared in the coronal plane similar to human slice preparation as described above.

A subset of cortical slices in both human and mouse were prepared using N-Methyl-D-glucamine (NMDG) protective recovery method [19]. The cortical tissue blocks were transferred and sectioned in 2–4 °C NMDG-HEPES aCSF solution containing (in mM): NMDG 92, KCl 2.5, NaH_2PO_4 1.25, $NaHCO_3$ 30, HEPES 20, Glucose 25, Thiourea 2, Na L-ascorbate 5, Na-Pyruvate 3, $CaCl_2.4H_2O$ 0.5, and $MgSO_4.7H_2O$. The pH of NMDG-HEPES aCSF solution was adjusted to 7.3–7.4 using hydrochloric acid and the osmolarity was 300–305 mOsm. The cortical slices were prepared using vibratome as described above. After slicing, slices were transferred to a recovery chamber filled with 32–34 °C NMDG-HEPES aCSF solution which continuously bubbled with 95% O_2–5% CO_2. After 12 min, the slices were transferred to an incubation solution-HEPES aCSF-containing (in mM): NaCl 92, KCl 2.5, NaH_2PO4.H_2O 1.25, $NaHCO_3$ 30, HEPES 20, Glucose 25, Thiourea 2, Na L-ascorbate 5, Na-Pyruvate 3, $CaCl_2.4H_2O$ 2, and

$MgSO_4.7H_2O$ 2. After 1-h incubation at room temperature, slices were transferred to a recording chamber and continuously perfused with aCSF containing (in mM): NaCl 126, KCl 2.5, $NaH_2PO_4.H_2O$ 1.25, $NaHCO_3$ 26, Glucose 12.6, $CaCl_2.2H_2O$ 2, and $MgSO_4.7H_20$ [6].

Whole-cell patch-clamp recording from human and mice cortical slices

For electrophysiological recordings, cortical slices were placed in a recording chamber mounted on a fixed-stage upright microscope (Axioskop 2 FS MOT; Carl Zeiss, Germany). Slices were continuously perfused with carbogenated (95% O_2/5% CO_2) aCSF containing of (in mM): NaCl 123, KCl 4, $CaCl_2.2H_2O$ 1.5, $MgSO_4.7H_2O$ 1.3, $NaHCO_3$ 26, $NaH_2PO4.H_2O$ 1.2, and D-glucose 10, pH 7.40 at 32-34 °C. Cortical neurons were visualized using an IR-CCD camera (IR-1000, MTI, USA) with a 40x water immersion objective. Patch pipettes (3-6 MΩ) were pulled from standard borosilicate glass pipettes (thin-wall borosilicate tubes with filaments, World Precision Instruments, Sarasota, FL, USA) using a vertical puller (PC-10, Narishige). For somatic recording of electrophysiological properties, patch pipettes were filled with intracellular solution containing (in mM): K-gluconate 135; NaCl 10; HEPES 10; $MgCl_2$ 1; Na_2ATP 2; GTP 0.3, pH adjusted with KOH to 7.4 (290–309 mOsm). A subset of data was collected with excitatory (APV 50 µM, Sigma; CNQX 25 µM, Sigma) and inhibitory (Bicuculline 10 µM, Sigma; CGP-35348 10 µM, Sigma) synaptic activity blocked.

Electrical signals were measured with Multiclamp 700A amplifier, Axopatch 200B amplifier, pClamp 9.2 and pClamp 10.6 data acquisition software (Axon instruments, Molecular Devices, USA). Subsequently, electrical signals were digitized at 20 kHz using a 1320X digitizer or a 1440A digitizer (Axon instruments, Molecular Devices, USA). The access resistance was monitored throughout the recording (typically between 8-20 MΩ), and neurons were discarded if the access resistance was > 25 MΩ. Recordings were not corrected for bridge balancing due to the short duration of recording time.We note that stimulus parameters for each recording are not

identical across recorded cells, in part due to technical considerations by the experimentalist, for example, to prevent losing the cell recording.

Axon Binary Format to Neurodata Without Borders file conversion

The x-to-nwb repository was used to convert current clamp recordings in axon binary format (ABF) to Neurodata Without Borders (NWB) format. Separate converters were used for files recorded using pClamp (pClamp, RRID:SCR_011323) 9.0, which output ABFv1 files, and pClamp >10.0, which output ABFv2 files, to ensure valid conversions while incorporating the essential metadata. The key aspects of our usage of these data conversion computer scripts relate to defining which ABF channels correspond to stimulus and response traces and ensuring that appropriate scale and offset factors are applied properly upon conversion. We incorporated the ndx-dandi-icephys metadata extensions to allow for inclusion of user-defined 'Subject' and 'Lab' metadata fields to be able to include specific metadata including 'subject_id', 'age', 'species', 'cell_id' and 'tissue_sample_id'.

Relevant metadata was recorded in two separate tables: first, patient level information, including demographics and clinical information; and second, recording specific information, which relate to aspects of each individual cell's recording, such as channels corresponding to stimulus, response, and resting membrane potential. The patient level demographics table included fields including 'Resection date', 'Resection procedure', 'Sex', 'Age', 'Years of seizure history', 'Diagnosis', 'Seizure type', 'Presence of a tumor', 'Antiepileptic drugs'. Recording specific metadata includes experiment 'date', 'Cell number' to differentiate recordings from distinct cells taken on the same day, 'Cell layer', 'Gain', 'Offset', 'Response channel', 'Command channel' and 'RMP' to record the resting membrane potential at the initial time of recording. Additional recording metadata was extracted directly from ABF files using custom scripts to extract the stimulus start and end times and the stimulus sampling rate.

Electrophysiology feature extraction

The Intrinsic Physiology Feature Extractor (IPFX) toolbox was used to extract features from converted NWB files [3,20]. All experiments consisted of long-square hyperpolarizing and depolarizing current injections, and extracted features include subthreshold features (i.e., input resistance, sag ratio), action potential properties (i.e., action potential half-width, threshold time and voltage) derived from the rheobase spike as well as multi-action potential spike train features derived from the IPFX-defined “hero” sweep (i.e., adaptation index), as described previously [6]. Our included metadata files contain stimulus start and end times along with an IPFX-compatible stimulus description ontology file for reproducibility and to facilitate the feature extraction process.

Quality control of contributed neuron recordings

We performed both automated and manual quality control checks of converted recordings to ensure dataset quality and maximize reuse potential. Using features automatically extracted via IPFX, we checked whether the baseline voltage of a sweep (i.e., v_baseline) deviated by more than 10mV from the initial measure in the first current injection step. Any cell recordings that had any sweep deviate beyond the 10mV threshold were not included in the final contributed dataset. We also included the measures for maximum drift of baseline Vm in each recording’s metadata under the field max_drift_Vm.

Also, individual recordings were manually inspected at 3 injected current steps (the most hyperpolarizing pulse, the rheobase and the most depolarizing step). In addition, we further manually inspected each neuron recording’s frequency/input curve to identify any abnormal responses, and also, to identify putative recordings from interneurons.

Following this manual inspection process, we note that in some instances, we observed some evidence for spike saturation at higher steps of current injection. We also noted some instances

of cell’s spiking spontaneously (i.e., spiking outside of the window of injected current), however, we chose not to reject these sweeps or cells according to our quality control criteria.

Statistical analyses

To detect statistical differences across experimental groupings, we report results using two sample t-test, Wilcoxon rank-sum test, Kruskal-Wallis rank sum test or Pearson correlation using the statistical functions in base R.
All statistical tests were performed using R version 4.1.2. [21]

Results

In Table 1, we summarize the three main axes differentiating the cells and recordings in this dataset. Namely, recordings differed by species (human versus mouse), by cortical layer of the cell body of the recorded neuron (Layer 23, Layer 3c, and Layer 5), and whether synaptic blockers were used in the external recording solution. Putative interneurons were identified by their action potential characteristics (large maximal firing rates and typically large spike after-hyperpolarization amplitudes) as described in Chameh, et al 2021. One reason why synaptic blockers were used is to make a subset of recordings more consistent with protocols used in other labs, such as the Allen Institute for Brain Sciences [3,11].

Dataset Overall Type	# cells	# individuals	Number of Pyr cells	Number of ints	L23	L3c	L5
Human (aCSF)	81	19	80	1	27	14	40 (1 int)
Human (aCSF containing synaptic	37	16	34	3	14 (2 int)	5 (1 int)	18

blockers)							
Mouse (aCSF containing synaptic blockers)	11	5	10	1	0	0	11 (1 int)

Table 1: Summary of number of electrophysiological recordings across major experimental conditions. Putative interneurons (ints) were identified by manual inspection of their electrophysiological characteristics.

In current clamp mode, hyperpolarizing and depolarizing current injections (600-1000 ms) were used to characterize biophysical features of cortical neurons, with examples from three recorded cells shown in Fig. 2.

Figure 2: Example voltage traces from 3 separate L5 neurons, a human L5 neuron recorded in standard aCSF (top), a human L5 neuron recorded in aCSF with synaptic blockers (2nd row), and from a mouse L5 neuron recorded in aCSF with synaptic blockers (third row). Bottom row shows the hyperpolarizing and depolarizing injected step currents that were applied to each neuron and includes the most hyperpolarizing current injection (blue), the rheobase (orange) and most depolarizing current injection (green).

In Fig. 3A, we highlight how the use of synaptic blockers in the external solution may affect recorded subthreshold neuronal properties. Specifically, among recorded human L5 neurons, there was a significant difference in the recorded input resistance between neurons recorded following application of synaptic blockers (208±106 MΩ, n=18) and regular aCSF (80.9±36.6 MΩ, n=40); t(19)=4.94, p=9.37e-05. However, in Fig. 3C there was no significant effect on the action potential width, t(28)=1.14, p=0.265, between neurons recorded following application of synaptics blockers and regular aCSF. Similarly, in Fig. 3E there was no detectable effect on

the average firing rate of the cell at the IPFX-defined “hero” sweep, t(40)=0.259, p=0.797, between the same groups.

To illustrate comparisons across species, in Fig. 3B, 3D and 3F, we show distributions of the input resistance, AP width and average firing rate of the “hero” sweep respectively recorded from neurons in both human and mouse cortical L5 neurons (in the presence of synaptic blockers). We did not detect a significant difference in the input resistance, t(11)=1.32, p=0.210, or average firing rate, t(25)=0.0709, p=0.944, observed between the recordings from the two species. However, when comparing the width of APs from recordings in human neurons (2.25±0.890 ms, n=18) and mouse neurons (1.55±0.783, n=11), there was a significant difference detected, t(23)=2.23, p=0.0355.

Figure 3: Distributions of input resistance, AP width and average hero-sweep firing frequency measured in L5 neurons across experimental conditions. Inclusion of synaptic blockers in aCSF has an observable effect on input resistance (A) but not AP width (C) or Avg Rate (E) as measured in human L5 neurons. Input resistance (B), AP width (D), and Avg Rate (F) are not significantly different across mouse and human in L5 neurons recorded in the presence of synaptic blockers in the external solution.

To compare the effect of solution used for the brain slice preparation on intrinsic properties, we compared the input resistance and sag ratio recorded following preparation in either solution. In Fig. 4A, we highlight a significant difference, t(16)=2.52, p = 0.0224, of higher measured input resistance in the recordings made following preparation in the NMDG (266±108 MΩ, n=12) recovery solution compared to the sucrose solution (179±75.4 MΩ, n=25). In Fig. 4B, we compare the sag ratio across the same conditions and observe no significant difference across

the brain slice preparations, t(13)=0.317, p=0.756. The statistical comparisons made in Fig. 4 were made after grouping all recordings from L23, L3C and L5 using standard aCSF. These comparisons emphasize the potential importance of the conditions used for the experimental preparation (see Discussion).

Figure 4: Distributions of input resistance (A) and sag ratio (B) measured in pooled human L2/3, L3C and L5 neurons derived from different slices, prepared using the NMDG protective solution and a standard sucrose solution preparations. All included recordings were performed using synaptic blockers in aCSF recording solution, with the molecular layer of each recording identified in red (L23), green (L3C) and blue (L5). The 2 recordings from L23 highlighted with a large point indicate the recordings used for the example traces of hyperpolarized steps in (C).

To illustrate the rich diversity of the metadata for each of the human recordings, in Fig. 5 we highlight specific comparisons of input resistance and sag ratio measurements recorded in regular aCSF across demographic conditions. Specifically, we focus on the input resistance as a fundamental passive electrophysiological property and the sag ratio as an active property that has previously been used to distinguish between subtypes of human neurons [6,11]. In Fig. 5A, 5C we compare distributions of these electrophysiological features across the three different brain lobes from which neuronal tissue was resected. Kruskal-Wallis rank sum test was used to examine whether brain lobe resection location on input resistance or measured sag ratio. No significant differences in input resistance (chi-squared = 2.7968, df = 2, p-value = 0.247), or sag ratio (chi-squared = 3.50, df = 2, p-value = 0.174), were found across the 3 resected locations. In Fig. 5B, 5D we compare the electrophysiological feature distributions measured in male and female patients. We did not detect any differences between recordings from male or female patients in input resistance, t(70)=1.38, p=0.172, or sag ratio, t(70)=0.0644, p=0.949. Note that all cells from frontal and parietal cortex were recorded from

tissue resected near the site of the epileptogenic focus, whereas all cells from temporal cortex were recorded distal from the epileptogenic focus (with the exception of 1 subject).

Figure 5: Distributions of input resistance (A, B) and sag ratio (C, D) recorded from pooled human L23, L3C and L5 neurons with standard aCSF. Measurements are grouped and compared by major brain lobe of resection location (A, C) and by sex (B, D).

Additionally, we illustrate the relationship of the input resistance and sag ratio against both patient age at time of surgical resection (Fig. 6A, B) and years of seizure experienced by the patient prior to the surgical intervention (Fig. 6C, D).

Figure 6: Scatterplots of input resistance and sag ratio vs patient age (A, B) and duration of seizure prior to surgical intervention (C, D) recorded in human L5 neurons with standard aCSF. Pearson's correlation values are reported within each plot.

Application Scenarios

The recordings in this database permit the quantification of biophysical properties from a diverse set of neurons, including human and mouse neurons with a well described set of metadata. Independent variables collected include age, sex, seizure history and cortical layer from which the tissue was resected. Additionally, experiments on the human neurons were performed with the use of synaptic blockers and without, allowing for comparisons and integration with other intrinsic electrophysiological databases comprising patch-clamp recordings, including from the Allen Cell types database (Allen Cell Types Database, RRID:SCR_014806) .

These data from current-clamp experiments are particularly beneficial for the development of conductance-based models of human neurons [11,22,23]. In particular, we highlight that in some instances, it may be more suitable to constrain biophysical models to human data in the absence of synaptic blockers, that is to say, when background synaptic activity is having a significant effect on input resistance measurements. The voltage responses can be used as a training set to constrain biophysical models when combined and integrated with other publicly available databases that provide relevant morphologies and channel kinetics, such as NeuroMorpho.org, Channelpedia or ICGenealogy [24–26]. Moreover, fitting biophysical models to data that is grouped based on demographic information can allow for cross-group comparisons using in silico approaches. Usage of these models in neuronal or circuit simulations can thus help to further predict and unveil the potential effects of differences in neuronal physiology across demographic groupings [27,28].

Discussion and Limitations

The repository provided is focused mainly on cortical neurons derived from human tissue. There are comparatively fewer recordings for analyses of mouse neuron function provided and all of these were performed using synaptic blockers that were shown to have a baseline effect on input resistance.

The recordings from human specimens derived from tissue during the surgical resection of diseased tissue for patients with intractable epilepsy or brain tumors. Along with having suffered seizures for an extended period of time, the patients may have concurrently taken one or a variety of anti-epileptic drugs that could have affected baseline neuronal excitability characteristics.

While these data were collected for the purpose of characterizing intrinsic properties of human neocortical neurons, we note that they were collected using different sets of experimental conditions, including those related to different recording solutions as well as cutting solutions. Our analyses suggest such experimental condition differences likely contribute to differences in downstream electrophysiological properties, and are consistent with prior analyses by ourselves and others [2,29]. For example, the observed effect of synaptic blockers on the input resistance may be due to reduction of overall membrane permeability as a consequence of the block of both excitatory and inhibitory conductances [30]. However, we did not observe a concurrent change in excitability characteristics such as the AP width, in agreement with previous findings that did not find a significant effect of synaptic blockers on AP characteristics or neuronal passive properties [31,32].

In contrast, the comparisons of electrophysiological measures following different cutting solutions highlight potential effects on neuronal excitability. We observe variability in the measured input resistance but consider that these effects may be due to changes in conductivity across the membrane or also experimental biases in selection of healthy neurons for patch-clamp protocol due to differential response to solutions of different osmolarity. Furthermore, there exists contrasting results in the literature on the effects of NMDG on neuronal excitability and synaptic transmission which shows the context-dependence of the many experimental variables [33,34]. Taken together, such potential differences in electrophysiological characteristics due to experimental conditions are important to consider when re-using these data in downstream analyses.

Data Availability

Both mouse and human data is available on the DANDI platform [35,36].

Conversion, analysis scripts and other associated metadata for recordings are available at GitHub [37]. All other supporting data and materials are available in the *GigaScience* GigaDB database [38].

Acknowledgements

We are grateful to our neurosurgical patients and their families for consenting to the use of their tissue samples for research. We thank Dr. Gelareh Zadeh and Dr. Mark Bernstein for their assistance in obtaining brain tissue samples and Victoria Barkley and Marjan Rafiee for assistance in compiling demographic and chart information. We thank Sara Mahallati and Iliya Weisspapir for assistance in tissue preparation. We thank Wataru Inoue and Michael Feyerabend for their critical comments on the manuscript. We acknowledge generous support from the Centre for Addiction and Mental Health Discovery Fund, Krembil Brain Institute Fund, National Institute of Health, and Kavli Foundations.

References

1. Tripathy SJ, Savitskaya J, Burton SD, Urban NN, Gerkin RC. NeuroElectro: a window to the world's neuron electrophysiology data. *Front Neuroinform*. 2014; doi: 10.3389/fninf.2014.00040.

2. Tripathy SJ, Burton SD, Geramita M, Gerkin RC, Urban NN. Brain-wide analysis of electrophysiological diversity yields novel categorization of mammalian neuron types. *J Neurophysiol*. 2015; doi: 10.1152/jn.00237.2015.

3. Gouwens NW, Sorensen SA, Berg J, Lee C, Jarsky T, Ting J, et al. Classification of electrophysiological and morphological neuron types in the mouse visual cortex. *Nat Neurosci*. 2019; doi: 10.1038/s41593-019-0417-0.

4. Markram H, Muller E, Ramaswamy S, Reimann MW, Abdellah M, Sanchez CA, et al. Reconstruction and Simulation of Neocortical Microcircuitry. *Cell*. 2015; doi:

10.1016/j.cell.2015.09.029.

5. Mohan H, Verhoog MB, Doreswamy KK, Eyal G, Aardse R, Lodder BN, et al. Dendritic and Axonal Architecture of Individual Pyramidal Neurons across Layers of Adult Human Neocortex. *Cereb Cortex*. 2015; doi: 10.1093/cercor/bhv188.

6. Chameh HM, Rich S, Wang L, Chen F-D, Zhang L, Carlen PL, et al. Diversity amongst human cortical pyramidal neurons revealed via their sag currents and frequency preferences. *Nat Commun*. Nature Publishing Group; 12:1–152021;

7. Berg J, Sorensen SA, Ting JT, Miller JA, Chartrand T, Buchin A, et al. Human neocortical expansion involves glutamatergic neuron diversification. *Nature*. 2021; doi: 10.1038/s41586-021-03813-8.

8. Gidon A, Zolnik TA, Fidzinski P, Bolduan F, Papoutsi A, Poirazi P, et al. Dendritic action potentials and computation in human layer 2/3 cortical neurons. *Science*. 2020; doi: 10.1126/science.aax6239.

9. Beaulieu-Laroche L, Toloza EHS, van der Goes M-S, Lafourcade M, Barnagian D, Williams ZM, et al. Enhanced Dendritic Compartmentalization in Human Cortical Neurons. *Cell*. 2018; doi: 10.1016/j.cell.2018.08.045.

10. Beaulieu-Laroche L, Brown NJ, Hansen M, Toloza EHS, Sharma J, Williams ZM, et al. Allometric rules for mammalian cortical layer 5 neuron biophysics. *Nature*. 2021; doi: 10.1038/s41586-021-04072-3.

11. Kalmbach BE, Buchin A, Long B, Close J, Nandi A, Miller JA, et al. h-Channels Contribute to Divergent Intrinsic Membrane Properties of Supragranular Pyramidal Neurons in Human versus Mouse Cerebral Cortex. *Neuron*. 2018; doi: 10.1016/j.neuron.2018.10.012.

12. Kalmbach BE, Hodge RD, Jorstad NL, Owen S, de Frates R, Yanny AM, et al. Signature morpho-electric, transcriptomic, and dendritic properties of human layer 5 neocortical pyramidal neurons. Neuron.

13. Hodge RD, Bakken TE, Miller JA, Smith KA, Barkan ER, Graybuck LT, et al. Conserved cell types with divergent features in human versus mouse cortex. *Nature*. 2019; doi: 10.1038/s41586-019-1506-7.

14. Deitcher Y, Eyal G, Kanari L, Verhoog MB, Atenekeng Kahou GA, Mansvelder HD, et al. Comprehensive Morpho-Electrotonic Analysis Shows 2 Distinct Classes of L2 and L3 Pyramidal Neurons in Human Temporal Cortex. *Cereb Cortex*. 2017; doi: 10.1093/cercor/bhx226.

15. Florez CM, McGinn RJ, Lukankin V, Marwa I, Sugumar S, Dian J, et al. In vitro recordings of human neocortical oscillations. *Cereb Cortex*. 2015; doi: 10.1093/cercor/bht235.

16. Mansouri A, Fallah A, Valiante TA. Determining surgical candidacy in temporal lobe epilepsy. *Epilepsy Res Treat*. 2012; doi: 10.1155/2012/706917.

17. Kostopoulos G, Drapeau C, Avoli M, Olivier A, Villemeure JG. Endogenous adenosine can reduce epileptiform activity in the human epileptogenic cortex maintained in vitro. *Neurosci Lett*. 1989; doi: 10.1016/0304-3940(89)90212-7.

18. Köhling R, Avoli M. Methodological approaches to exploring epileptic disorders in the human

brain in vitro. *J Neurosci Methods*. 2006; doi: 10.1016/j.jneumeth.2006.04.009.

19. Ting JT, Lee BR, Chong P, Soler-Llavina G, Cobbs C, Koch C, et al. Preparation of Acute Brain Slices Using an Optimized N-Methyl-D-glucamine Protective Recovery Method. *J Vis Exp*. 2018; doi: 10.3791/53825.

20. Lee BR, Budzillo A, Hadley K, Miller JA, Jarsky T, Baker K, et al. Scaled, high fidelity electrophysiological, morphological, and transcriptomic cell characterization. *Elife*. 2021; doi: 10.7554/eLife.65482.

21. Computing R, Others. R: A language and environment for statistical computing. *Vienna: R Core Team*. 2013;

22. Eyal G, Verhoog MB, Testa-Silva G, Deitcher Y, Lodder JC, Benavides-Piccione R, et al. Unique membrane properties and enhanced signal processing in human neocortical neurons. *Elife*. 2016; doi: 10.7554/eLife.16553.

23. Yao HK, Guet-McCreight A, Mazza F, Moradi Chameh H, Prevot TD, Griffiths JD, et al. Reduced inhibition in depression impairs stimulus processing in human cortical microcircuits. *Cell Rep*. 2022; doi: 10.1016/j.celrep.2021.110232.

24. Ascoli GA, Donohue DE, Halavi M. NeuroMorpho.Org: a central resource for neuronal morphologies. *J Neurosci*. 2007; doi: 10.1523/JNEUROSCI.2055-07.2007.

25. Ranjan R, Khazen G, Gambazzi L, Ramaswamy S, Hill SL, Schürmann F, et al. Channelpedia: an integrative and interactive database for ion channels. *Front Neuroinform*. 2011; doi: 10.3389/fninf.2011.00036.

26. Podlaski WF, Seeholzer A, Groschner LN, Miesenböck G, Ranjan R, Vogels TP. Mapping the function of neuronal ion channels in model and experiment. eLife.

27. Goriounova NA, Heyer DB, Wilbers R, Verhoog MB, Giugliano M, Verbist C, et al. Large and fast human pyramidal neurons associate with intelligence. *Elife*. 2018; doi: 10.7554/eLife.41714.

28. Guet-McCreight A, Chameh HM, Mahallati S, Wishart M, Tripathy SJ, Valiante TA, et al. Age-dependent increased sag current in human pyramidal neurons dampens baseline cortical activity. bioRxiv.

29. Tebaykin D, Tripathy SJ, Binnion N, Li B, Gerkin RC, Pavlidis P. Modeling sources of interlaboratory variability in electrophysiological properties of mammalian neurons. *J Neurophysiol*. 2018; doi: 10.1152/jn.00604.2017.

30. Núñez-Abades PA, Pattillo JM, Hodgson TM, Cameron WE. Role of synaptic inputs in determining input resistance of developing brain stem motoneurons. *J Neurophysiol*. 2000; doi: 10.1152/jn.2000.84.5.2317.

31. Ashwood TJ, Wheal HV. The expression of N-methyl-d-aspartate-receptor-mediated component during epileptiform synaptic activity in the hippocampus. British Journal of Pharmacology.

32. Nedergaard S. Regulation of action potential size and excitability in substantia nigra compacta neurons: sensitivity to 4-aminopyridine. *J Neurophysiol*. 1999; doi: 10.1152/jn.1999.82.6.2903.

33. Thuma JB, Hooper SL. Choline and NMDG directly reduce outward currents: reduced outward current when these substances replace Na+ is alone not evidence of Na+-activated K+ currents. *J Neurophysiol*. 2018; doi: 10.1152/jn.00871.2017.

34. Avegno EM, Middleton JW, Gilpin NW. Synaptic GABAergic transmission in the central amygdala (CeA) of rats depends on slice preparation and recording conditions. *Physiol Rep*. 2019; doi: 10.14814/phy2.14245.

35. Howard D, Chameh HM, Taufik V, Shreejoy T. UHN whole-cell patch-clamp excitability recordings from human cortical neurons (Version 0.220708.1652) [Data set]. DANDI archive. 2022. https://doi.org/10.48324/dandi.000293/0.220708.1652

36. Howard D, Chameh HM, Moradi H, Taufik V, Shreejoy T. UHN whole-cell patch-clamp excitability recordings from mouse cortical neurons (Version 0.220708.1652) [Data set]. DANDI archive. 2022. https://doi.org/10.48324/dandi.000292/0.220708.1652

37. : Associated metadata for recordings in GitHub. https://github.com/derekhoward/nwb_conversion/blob/master/data/processed/meta/metadata.csv

38. Howard D, Chameh HM, Guet-McCreight A, Hsiao HA, Vuong M, Seo YS, et al. Supporting data for “An in vitro whole-cell electrophysiology dataset of human cortical neurons.” GigaScience Database. 2022. http://doi.org/10.5524/102317.

Figure 1

Click here to access/download;Figure;Figure1.png

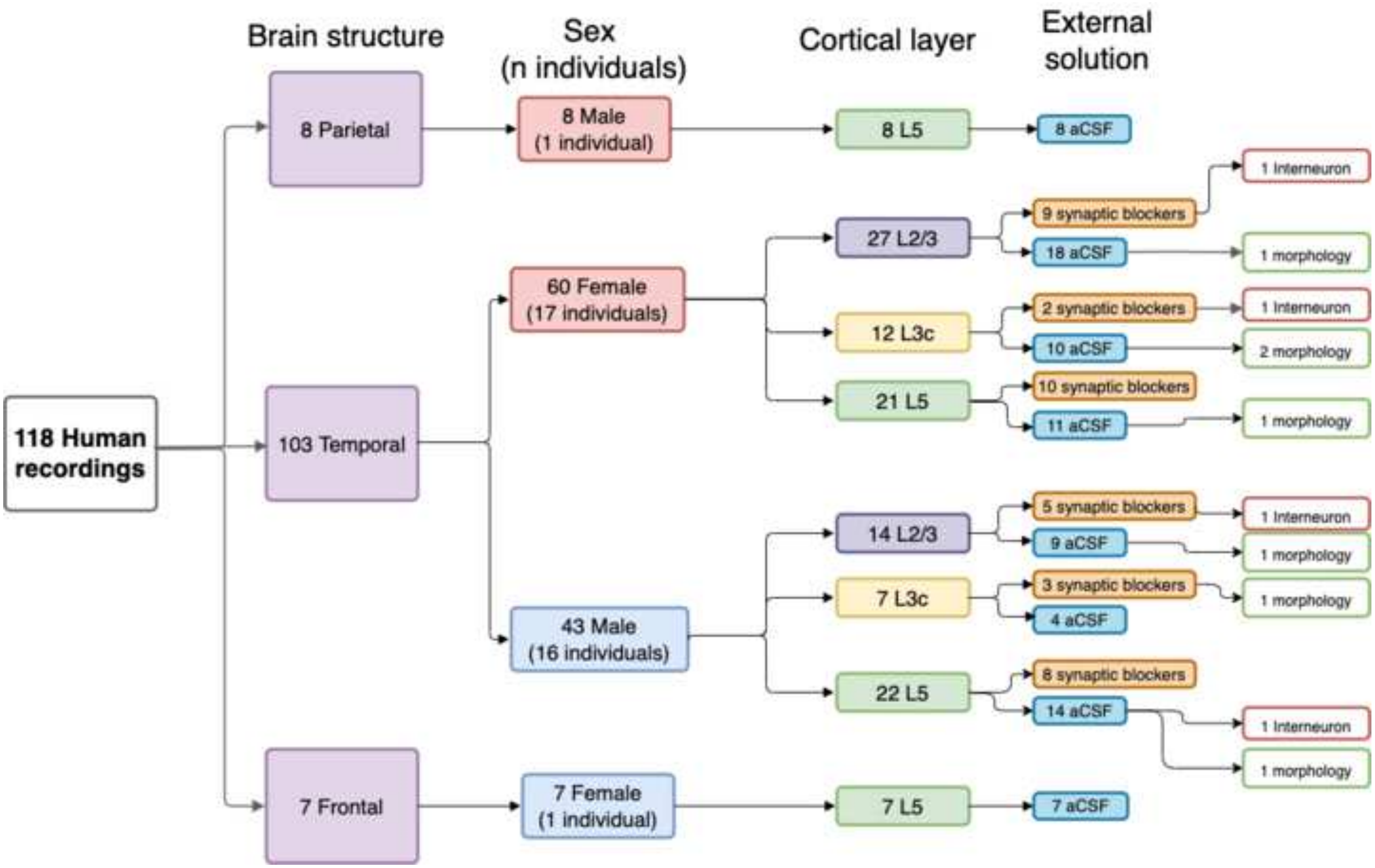

Figure 2

Click here to access/download;Figure;Figure2.png

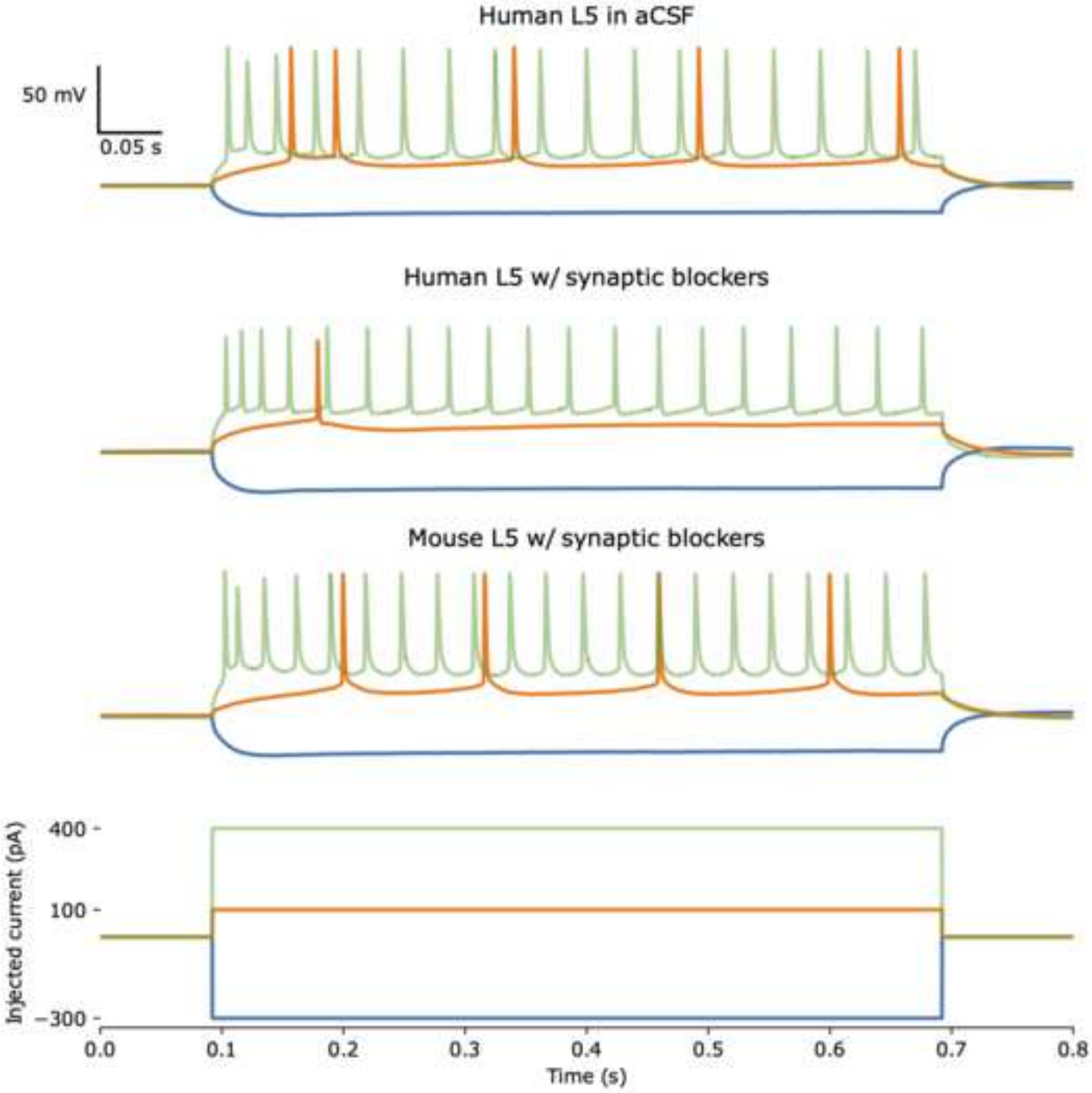

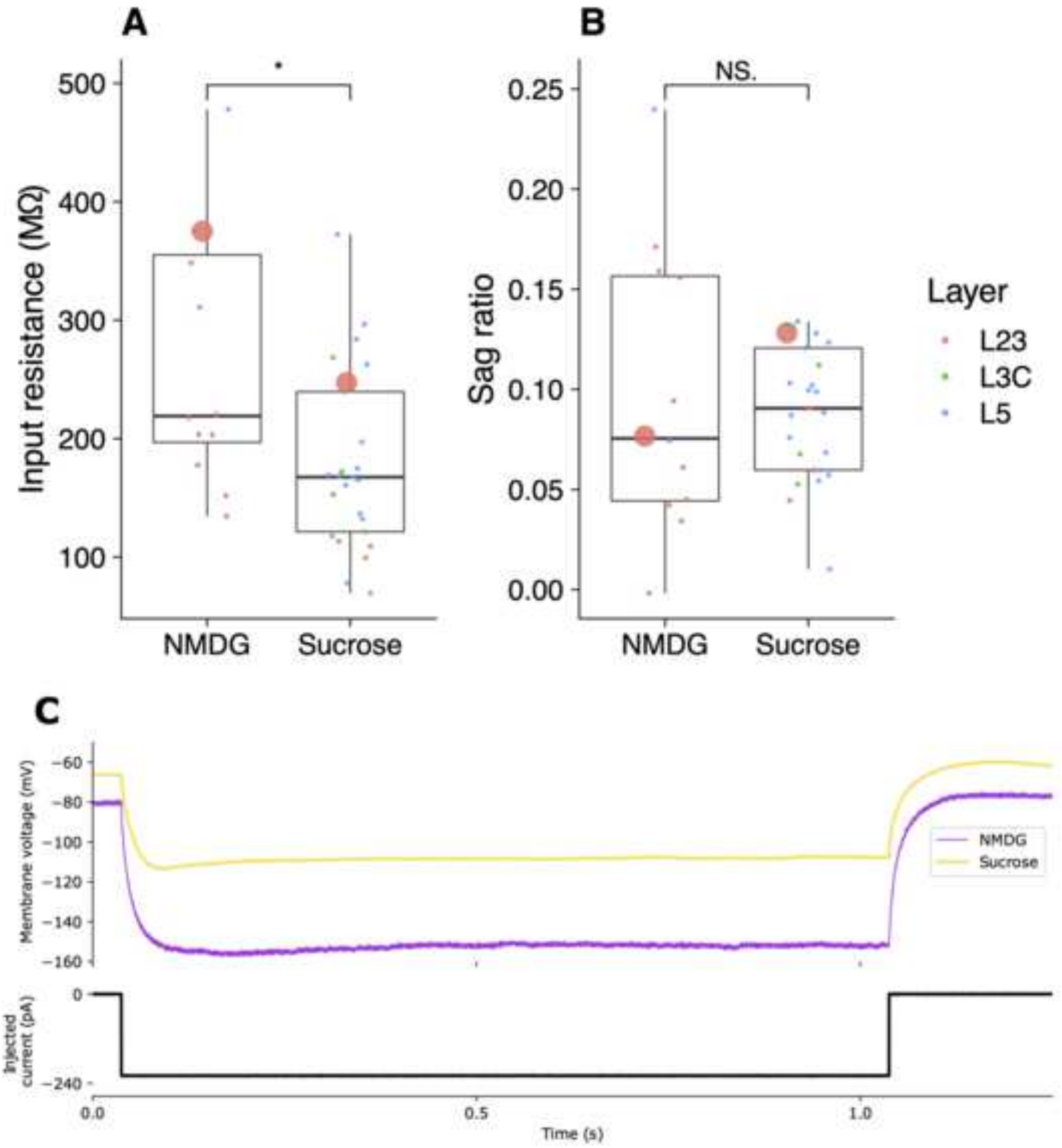
A
Input resistance (MΩ)
500
400
300
200
100
NMDG
Sucrose
B
NS.
Sag ratio
0.25
0.20
0.15
0.10
0.05
0.00
NMDG
Sucrose
Layer
L23
L3C
L5
C
Membrane voltage (mV)
-60
-80
-100
-120
-140
-160
NMDG
Sucrose
Injected current (pA)
0
-240
0.0
0.5
1.0
Time (s)

Figure 5

Click here to access/download;Figure;Figure5.png

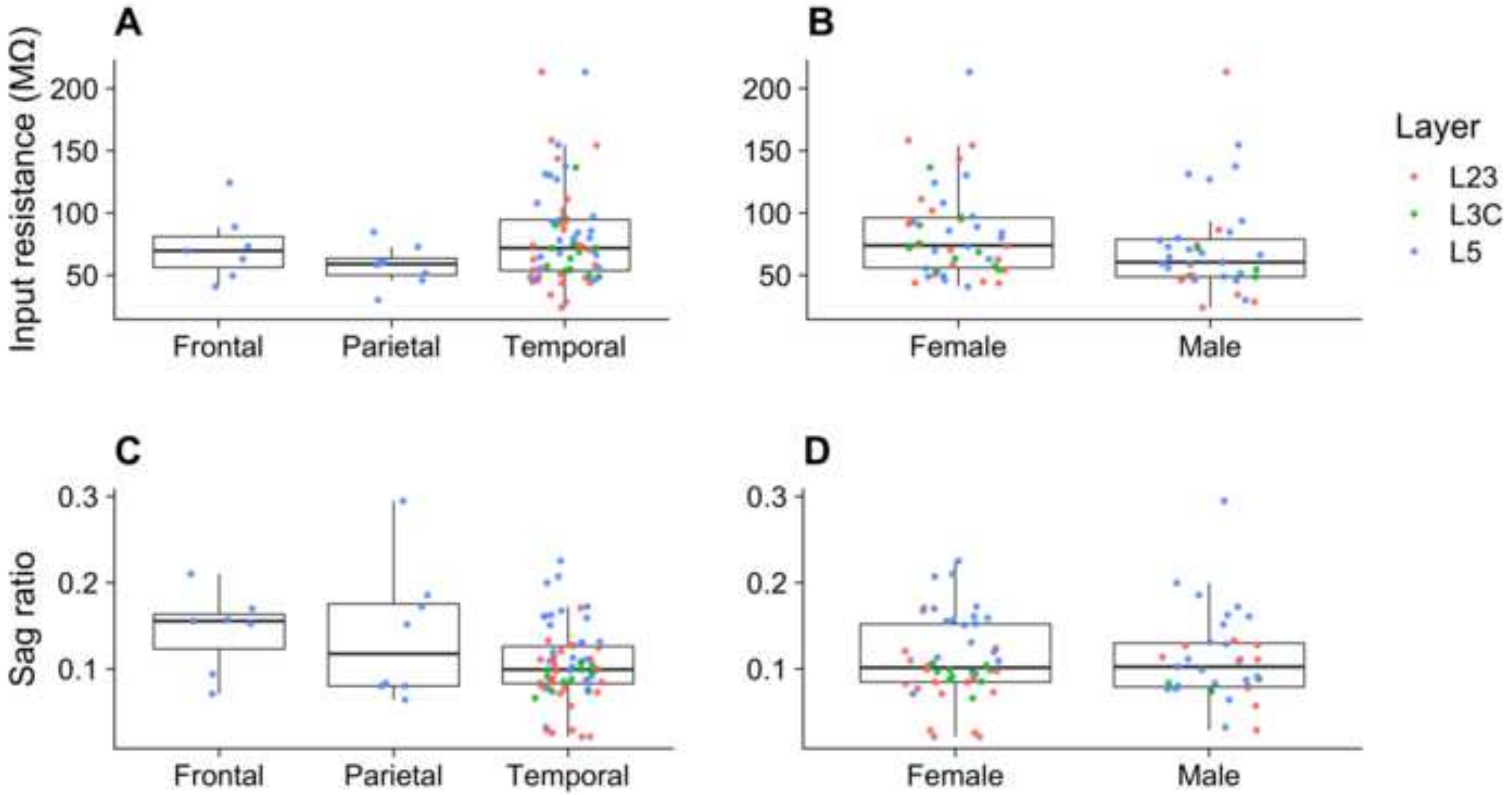

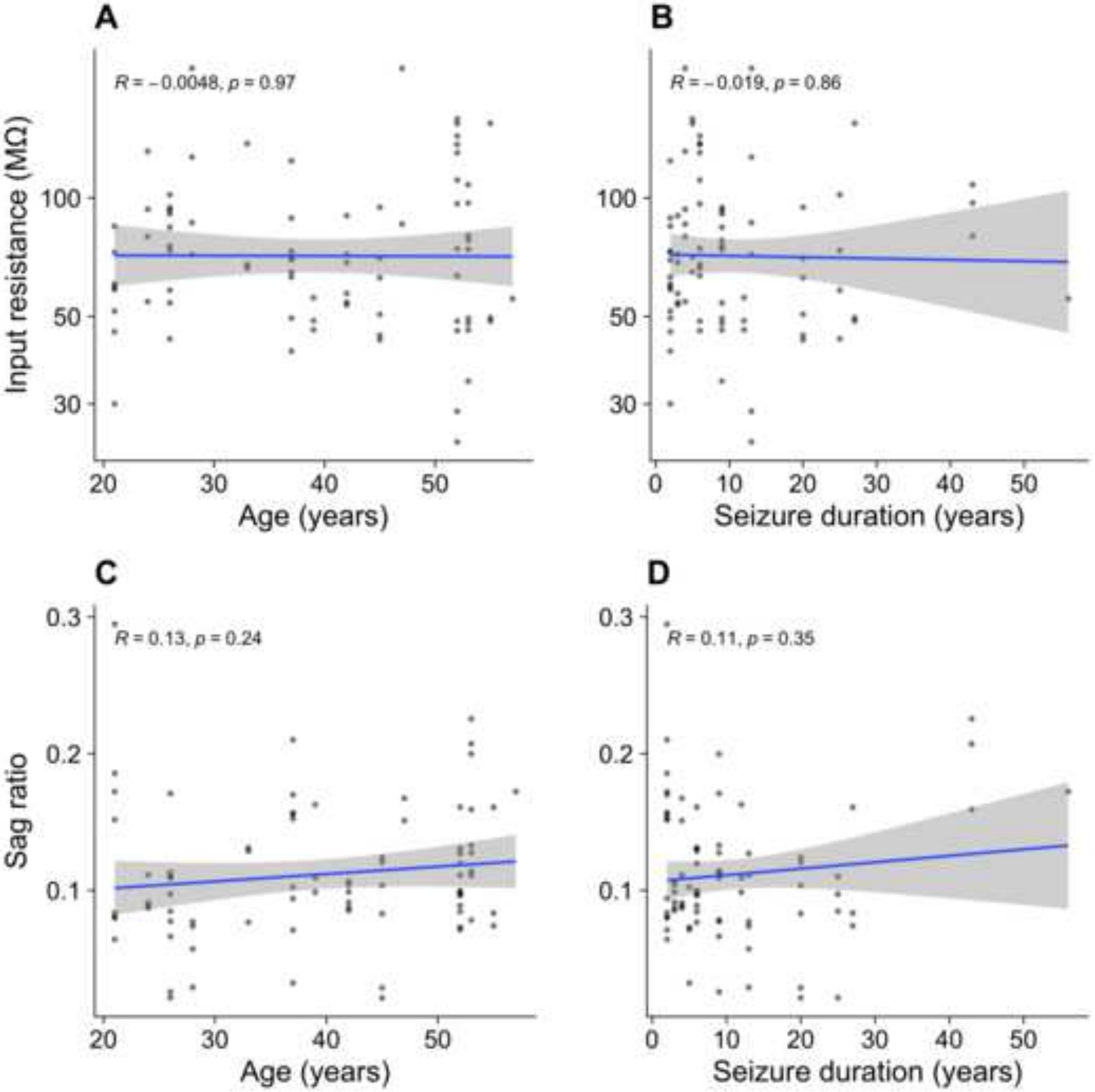
A
R = −0.0048, p = 0.97
Input resistance (MΩ)
100
50
30
20
30
40
50
Age (years)
B
R = −0.019, p = 0.86
100
50
30
0
10
20
30
40
50
Seizure duration (years)
C
R = 0.13, p = 0.24
Sag ratio
0.3
0.2
0.1
20
30
40
50
Age (years)
D
R = 0.11, p = 0.35
0.3
0.2
0.1
0
10
20
30
40
50
Seizure duration (years)

Figure 3

Click here to access/download;Figure;Figure3.png

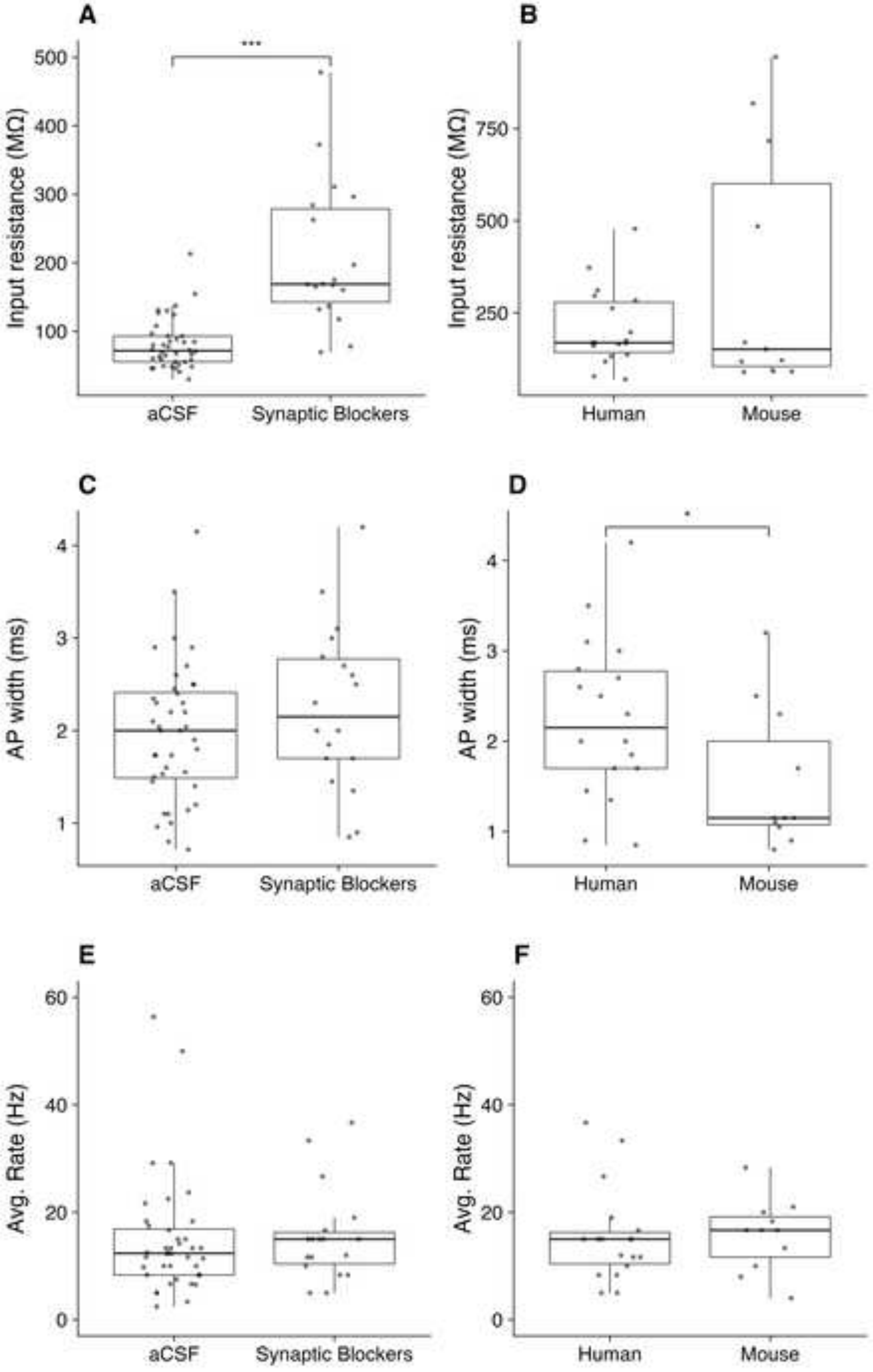

Supplement: giac108_GIGA-D-22-00068_Revision_2 [file giac108_giga-d-22-00068_revision_2.pdf]
