# Supplementary material for: An in vitro whole-cell electrophysiology dataset of human cortical neurons
Source: Gigascience. 2022 Nov 15;11:giac108. doi: 10.1093/gigascience/giac108 (PMC9664072; doi:10.1093/gigascience/giac108)
Supplement: giac108_Response_to_Reviewer_Comments_Original_Submission [file giac108_response_to_reviewer_comments_original_submission.pdf]

Author's Response To Reviewer Comments

Close

Reviewer #1:
In this work, Howard et al. present a dataset of electrophysiological recordings from human cortical tissue supplemented with recordings taken from mouse somatosensory cortex. Making such a dataset publicly available is a useful addition to the field and the data structure is clearly presented in Figure 1. The methods are appropriate and the manuscript overall is well-written. I am supportive of accepting this dataset as a Data Note, but also think there is room for improvement.

Major points:
1) I selected a few random recordings and most of the recordings seem to be of reasonable quality but there are some of which I wonder if they need to be included. For example:
2016_01_28_0012: shows spontaneous spikes and spike saturation towards later sweeps
2018_02_08_0001: the spikes in this recording appear a bit strange in shape and the trace appears noisier than other recordings. Was the access for this cell okay? Also the bridge balancing seems to be a bit off. Was bridge balancing applied for all recordings? Please describe this in the Methods section.
2016_02_04_0042: this cell has a resting membrane potential of -45 mV, which in my experience is not a sign of a healthy cell. I'm not familiar with human cells but I assume this is true across species.
2016_02_04_0015: There's a sudden jump in the resting membrane potential in this cell (sweep 11 - 12), also indicating a problem with the health of the cell/the recording

- I understand that human tissue is not trivial to come by and that the authors want to include as many of their recordings as possible, but I think the quality of the dataset could be improved by the application of more stringent selection criteria.

We thank the reviewer for their overall positive impressions of this work and attention to detail in reviewing the submitted dataset and traces from example cells.

Following careful consideration of the comments from both reviewers, we have taken steps to update and clarify the selection criteria for the cells and traces that we have made available. Most prominently, we have taken this reviewer's suggestion to use voltage deviation from baseline as a useful metric for excluding unhealthy cells or potentially problematic recordings (see details below). Now, we have excluded XXX cells, with YY human and ZZ mouse cell recordings remaining. In addition, we note that we did not explicitly correct for the bridge balance and have added this detail to the methods section.

In the updated Methods section, we have now included an additional section describing our Quality Control process that outlines our cell selection criteria (pasted below):

We performed both automated and manual quality control checks of converted recordings to ensure dataset quality and maximize reuse potential. Using IPFX, we checked whether the "v_baseline" feature of any current sweep in a recording deviated by more than 20% from the initial measure in the first depolarizing step. Any recordings that had any sweep deviate beyond the 20% threshold were not included in the dataset. Also, individual converted recordings were visualized at 3 injected current steps (the most hyperpolarizing pulse, the rheobase and the most depolarizing step), along with the recording's frequency/input curve to identify any abnormal responses and for identification of interneuron cell types. We note that in some instances, we observed spike saturation at higher steps of current injection as well as spontaneous spikes outside of the window of current step injection.
We performed both automated and manual quality control checks of converted recordings to ensure dataset quality and maximize reuse potential. Using features automatically extracted via IPFX, we checked whether the baseline voltage of a sweep (i.e., v_baseline) deviated by more than 10mV from the initial measure in the first current injection step. Any cell recordings that had any sweep deviate beyond the 10mV threshold were not included in the final contributed dataset. We also included the measures for maximum drift of baseline Vm in each recording's metadata under the field max_drift_Vm. Also, individual recordings were manually inspected at 3 injected current steps (the most hyperpolarizing pulse, the rheobase and the most depolarizing step). In addition, we further manually inspected each

neuron recording’s frequency/input curve to identify any abnormal responses, and also, to identify putative recordings from interneurons.
Following this manual inspection process, we note that in some instances, we observed some evidence for spike saturation at higher steps of current injection. We also noted some instances of cell’s spiking spontaneously (i.e., spiking outside of the window of injected current), however, we chose not to reject these sweeps or cells according to our quality control criteria.

2) Another parameter that would be useful to report, for instance in Figure 3, is the number or frequency of the action potentials, either as an input-output curve (frequency vs. current amplitude) or e.g. maximum firing frequency or frequency at a given current amplitude (see point 3).

- As requested, in Figure 3, we have now included the slope of the FI curve (“FI fit_slope”) and the average firing rate of the IPFX-defined “hero sweep” (“avg_rate”) as additional features that capture these specific aspects of neuron physiology.

3) A general remark, related to point 2: it appears that the block pulse amplitude sometimes goes up to e.g. 400 pA while in other recordings it only reaches e.g. 150 or 200 pA. This should be noted in the text and would benefit from an explanation why different maximum amplitudes were used as it could limit comparisons between recordings.

- We agree this information can limit potential comparisons across recordings and have included the following additional paragraph in the methods section to clarify the data collection process.
“The stimulus parameters for each recording were not identical across the recording conditions due to technical considerations by the experimentalist. The maximum stimulus may have been changed by the experimenter depending on the cell’s input resistance to prevent overload and losing the cell recording. For example, recordings originating from mouse neurons typically were not stimulated beyond 300 pA due to increased potential to overload and loss of the ongoing recording.”

4) The description of the statistical tests is missing from the Methods section, please add. Readability could be improved by writing e.g. "214±102 MΩ" rather than "M=214 MΩ, SD=102). Also please report not only the significantly different outcomes but also the non-significant ones, either in the main text or in the figure legend. Please report in each case the statistical test that was used, which is currently sometimes done but is often missing. Particularly for Figure 4A, the authors report "a consistent trend" without giving the statistical details.

- In the updated manuscript, we have implemented these changes as requested. Specifically, we have now updated the relevant sections for figures 3,4 and 5 for reporting statistical findings. We have updated Fig. 4 to compare recordings from the pooled layers L23, L3C and L5 and highlight the different layers with color. Additionally, we have added specific information in the methods regarding the statistical analyses.

5) Figure 5: Were statistical tests done for the data presented here? If yes, please report the outcomes; if not, please explain why not. For the comparison male vs. female: please clarify whether the authors pooled all recordings from all brain regions.
To be honest I'm not really seeing the added benefit of the comparison epilepsy vs. tumor especially given that, as the authors state, "most of these patients with tumors also have epilepsy" which makes this a bit of a messy comparison. In addition the number of datapoints is heavily skewed towards the 'epilepsy' group.

- We agree that the tumor vs epilepsy comparison added little benefit and have now removed this comparison from Figure 5. In the updated manuscript, we have pooled the recordings from all regions but now distinguish cells recorded from different layers using color. We have updated the text and associated figure legend to correspond to the updated figure based on your recommendations.

Minor points:
1) Given that the authors report differences in the electrophysiological properties that depend on the slice solution used (NMDG or sucrose-based), it would be useful to highlight in Figure 1 or at least in the data files (see minor point 2) which samples were prepared using which solution.

- We agree that enabling such a comparison is important for reuse of these datasets. As requested, we have now added a column to the metadata sheet, termed ‘dandi_id’, that will more easily facilitate the

matching of each recording uploaded to DANDI with its corresponding metadata as provided in our spreadsheet.

2) The authors made the electrophysiological recordings readily available. For the final submission it would be useful to structure (or name) the data in such a way that it is clear how the data files correspond to each subject. Currently it is unclear for me which recording comes from which subject/brain region/cell type etc.

- As requested, we have now added a column to the metadata sheet, termed 'dandi_id', that will facilitate the matching of each recording uploaded to DANDI with its corresponding metadata as provided in our spreadsheet.

3) Page 13 "we demonstrate that the inclusion … suprathreshold stimuli"
Is this in line with other research? Also, I suggest to rephrase 'noticeable' to e.g. 'significant' or 'detectable'

- We have added a sentence in the discussion with references to show how our findings are in line with other research:
However, we did not observe a concurrent change in excitability characteristics such as the AP width, in agreement with previous findings that did not find a significant effect of synaptic blockers on AP characteristics or neuronal passive properties [31], [32].

We thank the reviewer for their suggestions to improve the language and have changed any use of "noticeable" to be more clear.

4) Page 13 "Due to the confounding [factor?] … human cortex"
I think what the authors mean is that they want to reduce the variability due to different cortical layers? But I'm not sure, so I think this sentence would benefit from rewriting.

- We agree this section was confusing and have revisited Figure 4. We now pool the recordings across the L23/L3C/L5 layers for statistical comparison and color them to provide the readers more context about the different layer of origin of the neuronal recording.

5) Figure 4 could benefit from some raw traces to exemplify the differences

- We agree and have added example traces to figure 4 which highlight the selected recordings and the effect on input resistance in response to a hyperpolarizing pulse.

6) The data in Figure 6 could benefit from e.g. an R2 value to quantify the correlation

- We thank the reviewer for the suggestion and have included the values directly in an updated version of the plot.

Reviewer #2:
1) The properties of human cortical neurons are of high interest given their involvement in the particular cognitive abilities of humans; however, they cannot be obtained and studied as readily as in other organisms for obvious reasons. Therefore, publicly sharing the experimental results from human neurosurgical resections provides a considerable benefit to the community. The study of Howard et al. presents recordings from nearly 140 human neocortical neurons recorded from in vitro slices derived from neurosurgical tissues and makes them available via the DANDI archive. The manuscript is straightforward and primarily serves to describe the data set along its various dimensions (subject metadata, recording conditions, etc.), rather than describe novel findings derived from the data set. As such, it seems appropriately submitted here as a Data Note.

The data appear to be of high quality and are also of high utility, as mentioned above. However, the manuscript and data set would benefit from a few areas of improvement.

The box-and-whisker plots are informative, but in most cases they are comparing somewhat heterogenous groups of cells (i.e., cells from different layers, pyramidal cells and interneurons together).

It would be helpful to indicate these factors by coloring the individual data points, so that a reader might see if, for example, if there are any trends in the data that follow cortical layer.

- We thank the reviewer for this suggestion and have updated Figures 4 and 5 to more clearly color cells by relevant factors (layers, cell type, etc) to more clearly convey the differences across groups.

2) My main issues actually involved accessing the data rather than the manuscript itself. I am basing my comments on the metadata and feature files linked in the "Data Availability" section.

First, the metadata file contains more rows than there are cells described in the data set. For example, for subject 1914, there are seven rows with that identifier, but only two files in the DANDA archive with that identifier. Among those 7 entries, there are duplicate "Cell #" entries (C1 and C2 appear twice), and five of the entries have a "ZD status" of ZD (the other two have "n.a.") and those five also have empty values for "cutting_solution". Should those rows have been excluded from the spreadsheet? Do the multiple rows with C1 and C2 refer to the same cells? If so, what happened to cells C3, C4, and C5 in the DANDI data set? Also, the use of ZD is not discussed in the manuscript, so that field's presence in the file is a bit confusing as well.

In general, I'm not sure why there are a number of empty values in the metadata spreadsheet. "cutting_solution" is missing for a number of cells (including those mentioned above), and the mouse cells don't have an entry for layer. There are other inconsistencies in how the metadata are encoded; for example, the manuscript says there are four interneurons among the human cells (Table 1). If you filter the metadata file for the "putative_interneuron" field to be TRUE, only three human cells are present. But there is another cell that has an entry of "int" for the "aggregated_cell_layer" column - is this the fourth interneuron? And is its layer not known?

It also appears that the "Cell #" field is not always filled out correctly - for example, for the subject "X2019.11.28" all the cells have a value of "1" in this column. This makes it difficult to associate metadata with DANDI files, as discussed next.

- We appreciate this feedback and have re-organized the provided metadata sheets to be more clear and useful for potential re-use.

We now link to an updated metadata sheet that includes only the recordings and fields that are immediately relevant to the dataset published on DANDI (taking care to remove fields like whether cells received ZD - as this is not pertinent to any of the cells in this dataset). We have also fixed the highlighted inconsistency for the putative interneurons and their known layer of origin.

3) Overall, it is difficult to find the data associated with a particular cell. In DANDI, the data are organized by subject, but the individual files are named in way that seems to have no relation to other cell-specific identifiers. I resorted to downloading all the cells for a particular subject and then examining the `general/subject/description` field in each of the NWB files to find the corresponding file. It would be very helpful to have the DANDI filenames or URLs in the metadata file as well (or in an additional file). I know that the DANDI links are not permanent until the datasets are published (they appear to still be in Draft mode when I examined them), so perhaps the authors are waiting for that before putting that kind of file together. But the addition of that file is critical to easy access of the data.

- As requested, we have now added a column to the metadata sheet, termed ‘dandi_id’, that will more easily facilitate the matching of each recording uploaded to DANDI with its corresponding metadata as provided in our spreadsheet.

4) Finally, the authors mention that there are seven morphologies associated with the cells in the data set in the NeuroMorpho database, but again there are no direct links to these cells in the manuscript or metadata files.

- We have added a column for the neuromorpho_ID to link the recordings with the uploaded morphologies on Neuromorpho.org.

5) In a more general comment, I note the stimulus protocols presented to the cells in this data set are limited only to step current injections of ~0.5 s to 1 s. While this cannot be changed now, if the authors intend to add more cells to the data set, they might consider expanding the stimulus set to characterize the properties of their cells in other ways, which could enable more complex modeling efforts or

characterizations of human neuron types.

- We appreciate this comment and will take care to more systematically probe cells for stimuli of varied types in future studies.

Minor point:
In Application Scenarios, paragraph 2: The sentence "In particular, we highlight that in some instances, it may be more suitable to constrain biophysical models to human data in the presence of synaptic blockers, that is to say, when background synaptic activity is having a significant effect on input resistance measurements." This seems self-contradictory; did the authors perhaps mean "absence" instead of "presence"?

- Yes, we have corrected this sentence. Thank you for pointing out this error.

Close
