# Supplementary material for: An in vitro whole-cell electrophysiology dataset of human cortical neurons
Source: Gigascience. 2022 Nov 15;11:giac108. doi: 10.1093/gigascience/giac108 (PMC9664072; doi:10.1093/gigascience/giac108)
Supplement: giac108_Response_to_Reviewer_Comments_Revision_1 [file giac108_response_to_reviewer_comments_revision_1.pdf]

# Author's Response To Reviewer Comments

Close

We thank the reviewer for their helpful suggestions in improving the clarity of the manuscript. We have addressed the concerns with remarks below:

1) "As requested, in Figure 3, we have now included the slope of the FI curve ("FI fit_slope") and the average firing rate of the IPFX-defined "hero sweep" ("avg_rate") as additional features that capture these specific aspects of neuron physiology."
Thank you for incorporating this parameter. In Figure 3E and 3F I do see "Avg. Rate (Hz)" but note the slope. Perhaps a mislabeled axis?

- We mistakenly mentioned the FI fit_slope in the last response while including only the ‘avg_rate’ as a parameter to capture excitability characteristics. 3E and 3F now share a y-axis to better facilitate the comparisons across experimental conditions in human neurons (3E) and across species (3F).

2) "Additionally, we have added specific information in the methods regarding the statistical analyses."
More information on the statistics has indeed been added which has improved the manuscript. However either in the main text or legend I would still suggest that non-significant findings also have a p-value that should be reported which is not always done. Currently the text states e.g. "We performed statistical comparisons for each of these groups yet note that no significant differences were observed at the $p < 0.05$ threshold."

Also there are still some inconsistencies in the reporting. For example:
"(NMDG: 266±108 MΩ, n=12; sucrose: 179±75.4 MΩ, n=25; t.test, $p < 0.05$)" Here (and where it is appropriate) I would suggest to report the p-value more precisely (e.g. is it 0.013 or 0.049?)
"(NMDG: 0.0960±0.0706, n=12; sucrose: 0.0892±0.0333, n=25, p=0.756)" Here the p-value is reported precisely but the test is not reported. I assume this was also tested using a t-test? Please report throughout the manuscript

- We have revisited this section to more precisely report the statistics in the text and have also updated later sections (for Fig 4 and 5) to more clearly communicate statistical findings in the text. Pasted section for Fig3 below:

“In Fig. 3A, we highlight how the use of synaptic blockers in the external solution may affect recorded subthreshold neuronal properties. Specifically, among recorded human L5 neurons, there was a significant difference in the recorded input resistance between neurons recorded following application of synaptic blockers (208±106 MΩ, n=18) and regular aCSF (80.9±36.6 MΩ, n=40); $t(19)=4.94$, p=9.37e-05. However, in Fig. 3C there was no significant effect on the action potential width, $t(28)=1.14$, p=0.265, between neurons recorded following application of synaptics blockers and regular aCSF. Similarly, in Fig. 3E there was no detectable effect on the average firing rate of the cell at the IPFX-defined “hero” sweep, $t(40)=0.259$, p=0.797, between the same groups. To illustrate comparisons across species, in Fig. 3B, 3D and 3F, we show distributions of the input resistance, AP width and average firing rate of the “hero” sweep respectively recorded from neurons in both human and mouse cortical L5 neurons (in the presence of synaptic blockers). We did not detect a significant difference in the input resistance, $t(11)=1.32$, p=0.210, or average firing rate, $t(25)=0.0709$, p=0.944, observed between the recordings from the two species. However, when comparing the width of APs from recordings in human neurons (2.25±0.890 ms, n=18) and mouse neurons (1.55±0.783, n=11), there was a significant difference detected, $t(23)=2.23$, p=0.0355.‟

3) For Figure 5 statistical comparisons are still missing. Please report the outcomes. Or, if it is not useful to make statistical comparisons please explain in the main text.

- We now mention the statistical comparisons directly in the text. Pasted below:
“In Fig. 5A, 5C we compare distributions of these electrophysiological features across the three different

brain lobes from which neuronal tissue was resected. Kruskal-Wallis rank sum test was used to examine whether brain lobe resection location on input resistance or measured sag ratio. No significant differences in input resistance (chi-squared = 2.7968, df = 2, p-value = 0.247), or sag ratio (chi-squared = 3.50, df = 2, p-value = 0.174), were found across the 3 resected locations. In Fig. 5B, 5D we compare the electrophysiological feature distributions measured in male and female patients. We did not detect any differences between recordings from male or female patients in input resistance, t(70)=1.38, p=0.172, or sag ratio, t(70)=0.0644, p=0.949."

Close
