# Supplementary material for: An in vitro whole-cell electrophysiology dataset of human cortical neurons
Source: Gigascience. 2022 Nov 15;11:giac108. doi: 10.1093/gigascience/giac108 (PMC9664072; doi:10.1093/gigascience/giac108)
Supplement: giac108_Reviewer_1_Report_Original_Submission — Koen Kole -- 5/11/2022 Reviewed [file giac108_reviewer_1_report_original_submission.pdf]

Reviewer Report

Title: An in vitro whole-cell electrophysiology dataset of human cortical neurons

Version: Original Submission **Date:** 5/11/2022

Reviewer name: Koen Kole

Reviewer Comments to Author:

In this work, Howard et al. present a dataset of electrophysiological recordings from human cortical tissue supplemented with recordings taken from mouse somatosensory cortex. Making such a dataset publicly available is a useful addition to the field and the data structure is clearly presented in Figure 1. The methods are appropriate and the manuscript overall is well-written. I am supportive of accepting this dataset as a Data Note, but also think there is room for improvement.
Major points:
1) I selected a few random recordings and most of the recordings seem to be of reasonable quality but there are some of which I wonder if they need to be included. For example:
2016_01_28_0012: shows spontaneous spikes and spike saturation towards later sweeps
2018_02_08_0001: the spikes in this recording appear a bit strange in shape and the trace appears noisier than other recordings. Was the access for this cell okay? Also the bridge balancing seems to be a bit off. Was bridge balancing applied for all recordings? Please describe this in the Methods section.
2016_02_04_0042: this cell has a resting membrane potential of -45 mV, which in my experience is not a sign of a healthy cell. I'm not familiar with human cells but I assume this is true across species.
2016_02_04_0015: There's a sudden jump in the resting membrane potential in this cell (sweep 11 - 12), also indicating a problem with the health of the cell/the recording
I understand that human tissue is not trivial to come by and that the authors want to include as many of their recordings as possible, but I think the quality of the dataset could be improved by the application of more stringent selection criteria.
2) Another parameter that would be useful to report, for instance in Figure 3, is the number or frequency of the action potentials, either as an input-output curve (frequency vs. current amplitude) or e.g. maximum firing frequency or frequency at a given current amplitude (see point 3).
3) A general remark, related to point 2: it appears that the block pulse amplitude sometimes goes up to e.g. 400 pA while in other recordings it only reaches e.g. 150 or 200 pA. This should be noted in the text and would benefit from an explanation why different maximum amplitudes were used as it could limit comparisons between recordings.
4) The description of the statistical tests is missing from the Methods section, please add. Readability could be improved by writing e.g. "214Â±102 MÎ©" rather than "M=214 MÎ©, SD=102). Also please report not only the significantly different outcomes but also the non-significant ones, either in the main text or in the figure legend. Please report in each case the statistical test that was used, which is currently sometimes done but is often missing. Particularly for Figure 4A, the authors report "a consistent trend" without giving the statistical details.
5) Figure 5: Were statistical tests done for the data presented here? If yes, please report the outcomes;

if not, please explain why not. For the comparison male vs. female: please clarify whether the authors pooled all recordings from all brain regions.
To be honest I'm not really seeing the added benefit of the comparison epilepsy vs. tumor especially given that, as the authors state, "most of these patients with tumors also have epilepsy" which makes this a bit of a messy comparison. In addition the number of datapoints is heavily skewed towards the 'epilepsy' group.
Minor points:
1) Given that the authors report differences in the electrophysiological properties that depend on the slice solution used (NMDG or sucrose-based), it would be useful to highlight in Figure 1 or at least in the data files (see minor point 2) which samples were prepared using which solution.
2) The authors made the electrophysiological recordings readily available. For the final submission it would be useful to structure (or name) the data in such a way that it is clear how the data files correspond to each subject. Currently it is unclear for me which recording comes from which subject/brain region/cell type etc.
3) Page 13 "we demonstrate that the inclusion … suprathreshold stimuli"
Is this in line with other research? Also, I suggest to rephrase 'noticeable' to e.g. 'significant' or 'detectable'
4) Page 13 "Due to the confounding [factor?] … human cortex"
I think what the authors mean is that they want to reduce the variability due to different cortical layers? But I'm not sure, so I think this sentence would benefit from rewriting.
5) Figure 4 could benefit from some raw traces to exemplify the differences
6) The data in Figure 6 could benefit from e.g. an R2 value to quantify the correlation

Level of Interest

Please indicate how interesting you found the manuscript: Choose an item.

Quality of Written English

Please indicate the quality of language in the manuscript: Choose an item.

Declaration of Competing Interests

Please complete a declaration of competing interests, considering the following questions:

- Have you in the past five years received reimbursements, fees, funding, or salary from an organisation that may in any way gain or lose financially from the publication of this manuscript, either now or in the future?
- Do you hold any stocks or shares in an organisation that may in any way gain or lose financially from the publication of this manuscript, either now or in the future?

- Do you hold or are you currently applying for any patents relating to the content of the manuscript?
- Have you received reimbursements, fees, funding, or salary from an organization that holds or has applied for patents relating to the content of the manuscript?
- Do you have any other financial competing interests?
- Do you have any non-financial competing interests in relation to this paper?

If you can answer no to all of the above, write 'I declare that I have no competing interests' below. If your reply is yes to any, please give details below.

I declare that I have no competing interests

I agree to the open peer review policy of the journal. I understand that my name will be included on my report to the authors and, if the manuscript is accepted for publication, my named report including any attachments I upload will be posted on the website along with the authors' responses. I agree for my report to be made available under an Open Access Creative Commons CC-BY license (http://creativecommons.org/licenses/by/4.0/). I understand that any comments which I do not wish to be included in my named report can be included as confidential comments to the editors, which will not be published.

Choose an item.

To further support our reviewers, we have joined with Publons, where you can gain additional credit to further highlight your hard work (see: https://publons.com/journal/530/gigascience). On publication of this paper, your review will be automatically added to Publons, you can then choose whether or not to claim your Publons credit. I understand this statement.

Yes Choose an item.
