# Supplementary material for: An in vitro whole-cell electrophysiology dataset of human cortical neurons
Source: Gigascience. 2022 Nov 15;11:giac108. doi: 10.1093/gigascience/giac108 (PMC9664072; doi:10.1093/gigascience/giac108)
Supplement: giac108_Reviewer_2_Report_Original_Submission — Nathan Gouwens -- 5/13/2022 Reviewed [file giac108_reviewer_2_report_original_submission.pdf]

Reviewer Report

Title: An in vitro whole-cell electrophysiology dataset of human cortical neurons

Version: Original Submission **Date:** 5/13/2022

Reviewer name: Nathan Gouwens

Reviewer Comments to Author:

The properties of human cortical neurons are of high interest given their involvement in the particular cognitive abilities of humans; however, they cannot be obtained and studied as readily as in other organisms for obvious reasons. Therefore, publicly sharing the experimental results from human neurosurgical resections provides a considerable benefit to the community. The study of Howard et al. presents recordings from nearly 140 human neocortical neurons recorded from in vitro slices derived from neurosurgical tissues and makes them available via the DANDI archive. The manuscript is straightforward and primarily serves to describe the data set along its various dimensions (subject metadata, recording conditions, etc.), rather than describe novel findings derived from the data set. As such, it seems appropriately submitted here as a Data Note.

The data appear to be of high quality and are also of high utility, as mentioned above. However, the manuscript and data set would benefit from a few areas of improvement.

The box-and-whisker plots are informative, but in most cases they are comparing somewhat heterogenous groups of cells (i.e., cells from different layers, pyramidal cells and interneurons together). It would be helpful to indicate these factors by coloring the individual data points, so that a reader might see if, for example, if there are any trends in the data that follow cortical layer.

My main issues actually involved accessing the data rather than the manuscript itself. I am basing my comments on the metadata and feature files linked in the "Data Availability" section.

First, the metadata file contains more rows than there are cells described in the data set. For example, for subject 1914, there are seven rows with that identifier, but only two files in the DANDA archive with that identifier. Among those 7 entries, there are duplicate "Cell #" entries (C1 and C2 appear twice), and five of the entries have a "ZD status" of ZD (the other two have "n.a.") and those five also have empty values for "cutting_solution". Should those rows have been excluded from the spreadsheet? Do the multiple rows with C1 and C2 refer to the same cells? If so, what happened to cells C3, C4, and C5 in the DANDI data set? Also, the use of ZD is not discussed in the manuscript, so that field's presence in the file is a bit confusing as well.

In general, I'm not sure why there are a number of empty values in the metadata spreadsheet. "cutting_solution" is missing for a number of cells (including those mentioned above), and the mouse cells don't have an entry for layer. There are other inconsistencies in how the metadata are encoded; for example, the manuscript says there are four interneurons among the human cells (Table 1). If you filter the metadata file for the "putative_interneuron" field to be TRUE, only three human cells are present. But there is another cell that has an entry of "int" for the "aggregated_cell_layer" column - is this the fourth interneuron? And is its layer not known?

It also appears that the "Cell #" field is not always filled out correctly - for example, for the subject

"X2019.11.28" all the cells have a value of "1" in this column. This makes it difficult to associate metadata with DANDI files, as discussed next.
Overall, it is difficult to find the data associated with a particular cell. In DANDI, the data are organized by subject, but the individual files are named in way that seems to have no relation to other cell-specific identifiers. I resorted to downloading all the cells for a particular subject and then examining the `general/subject/description`field in each of the NWB files to find the corresponding file. It would be very helpful to have the DANDI filenames or URLs in the metadata file as well (or in an additional file). I know that the DANDI links are not permanent until the datasets are published (they appear to still be in Draft mode when I examined them), so perhaps the authors are waiting for that before putting that kind of file together. But the addition of that file is critical to easy access of the data.
Finally, the authors mention that there are seven morphologies associated with the cells in the data set in the NeuroMorpho database, but again there are no direct links to these cells in the manuscript or metadata files.
In a more general comment, I note the stimulus protocols presented to the cells in this data set are limited only to step current injections of ~0.5 s to 1 s. While this cannot be changed now, if the authors intend to add more cells to the data set, they might consider expanding the stimulus set to characterize the properties of their cells in other ways, which could enable more complex modeling efforts or characterizations of human neuron types.
Minor point:
- In Application Scenarios, paragraph 2: The sentence "In particular, we highlight that in some instances, it may be more suitable to constrain biophysical models to human data in the presence of synaptic blockers, that is to say, when background synaptic activity is having a significant effect on input resistance measurements." This seems self-contradictory; did the authors perhaps mean "absence" instead of "presence"?

Level of Interest

Please indicate how interesting you found the manuscript: Choose an item.

Quality of Written English

Please indicate the quality of language in the manuscript: Choose an item.

Declaration of Competing Interests

Please complete a declaration of competing interests, considering the following questions:

- Have you in the past five years received reimbursements, fees, funding, or salary from an organisation that may in any way gain or lose financially from the publication of this manuscript, either now or in the future?
- Do you hold any stocks or shares in an organisation that may in any way gain or lose financially from the publication of this manuscript, either now or in the future?

- Do you hold or are you currently applying for any patents relating to the content of the manuscript?
- Have you received reimbursements, fees, funding, or salary from an organization that holds or has applied for patents relating to the content of the manuscript?
- Do you have any other financial competing interests?
- Do you have any non-financial competing interests in relation to this paper?

If you can answer no to all of the above, write 'I declare that I have no competing interests' below. If your reply is yes to any, please give details below.

I declare that I have no competing interests

I agree to the open peer review policy of the journal. I understand that my name will be included on my report to the authors and, if the manuscript is accepted for publication, my named report including any attachments I upload will be posted on the website along with the authors' responses. I agree for my report to be made available under an Open Access Creative Commons CC-BY license (http://creativecommons.org/licenses/by/4.0/). I understand that any comments which I do not wish to be included in my named report can be included as confidential comments to the editors, which will not be published.

Choose an item.

To further support our reviewers, we have joined with Publons, where you can gain additional credit to further highlight your hard work (see: https://publons.com/journal/530/gigascience). On publication of this paper, your review will be automatically added to Publons, you can then choose whether or not to claim your Publons credit. I understand this statement.

Yes Choose an item.
