# Supplementary material for: An in vitro whole-cell electrophysiology dataset of human cortical neurons
Source: Gigascience. 2022 Nov 15;11:giac108. doi: 10.1093/gigascience/giac108 (PMC9664072; doi:10.1093/gigascience/giac108)
Supplement: giac108_Reviewer_2_Report_Revision_1 — Koen Kole -- 8/23/2022 Reviewed [file giac108_reviewer_2_report_revision_1.pdf]

Reviewer Report

Title: An in vitro whole-cell electrophysiology dataset of human cortical neurons

Version: Revision 1 **Date:** 8/23/2022

Reviewer name: Koen Kole

Reviewer Comments to Author:

The authors have made significant improvements to the manuscript. There are only a few minor points left that I would suggest to be addressed:
1) "As requested, in Figure 3, we have now included the slope of the FI curve ("FI fit_slope") and the average firing rate of the IPFX-defined "hero sweep" ("avg_rate") as additional features that capture these specific aspects of neuron physiology."
Thank you for incorporating this parameter. In Figure 3E and 3F I do see "Avg. Rate (Hz)" but note the slope. Perhaps a mislabeled axis?
2) "Additionally, we have added specific information in the methods regarding the statistical analyses."
More information on the statistics has indeed been added which has improved the manuscript. However either in the main text or legend I would still suggest that non-significant findings also have a p-value that should be reported which is not always done. Currently the text states e.g. "We performed statistical comparisons for each of these groups yet note that no significant differences were observed at the p < 0.05 threshold."
Also there are still some inconsistencies in the reporting. For example:
"(NMDG: 266Â±108 MÎ©, n=12; sucrose: 179Â±75.4 MÎ©, n=25; t.test, p < 0.05)" Here (and where it is appropriate) I would suggest to report the p-value more precisely (e.g. is it 0.013 or 0.049?)
"(NMDG: 0.0960Â±0.0706, n=12; sucrose: 0.0892Â±0.0333, n=25, p=0.756)" Here the p-value is reported precisely but the test is not reported. I assume this was also tested using a t-test? Please report throughout the manuscript
3) For Figure 5 statistical comparisons are still missing. Please report the outcomes. Or, if it is not useful to make statistical comparisons please explain in the main text.
Otherwise the authors adequately addressed my previous points.

Level of Interest

Please indicate how interesting you found the manuscript: Choose an item.

Quality of Written English

Please indicate the quality of language in the manuscript: Choose an item.

Declaration of Competing Interests

Please complete a declaration of competing interests, considering the following questions:

- Have you in the past five years received reimbursements, fees, funding, or salary from an organisation that may in any way gain or lose financially from the publication of this manuscript, either now or in the future?
- Do you hold any stocks or shares in an organisation that may in any way gain or lose financially from the publication of this manuscript, either now or in the future?
- Do you hold or are you currently applying for any patents relating to the content of the manuscript?
- Have you received reimbursements, fees, funding, or salary from an organization that holds or has applied for patents relating to the content of the manuscript?
- Do you have any other financial competing interests?
- Do you have any non-financial competing interests in relation to this paper?

If you can answer no to all of the above, write 'I declare that I have no competing interests' below. If your reply is yes to any, please give details below.

I declare that I have no competing interests

I agree to the open peer review policy of the journal. I understand that my name will be included on my report to the authors and, if the manuscript is accepted for publication, my named report including any attachments I upload will be posted on the website along with the authors' responses. I agree for my report to be made available under an Open Access Creative Commons CC-BY license (http://creativecommons.org/licenses/by/4.0/). I understand that any comments which I do not wish to be included in my named report can be included as confidential comments to the editors, which will not be published.

Choose an item.

To further support our reviewers, we have joined with Publons, where you can gain additional credit to further highlight your hard work (see: https://publons.com/journal/530/gigascience). On publication of this paper, your review will be automatically added to Publons, you can then choose whether or not to claim your Publons credit. I understand this statement.

Yes Choose an item.
